# Differential protein abundance in colostrum-derived extracellular vesicles from bovine leukemia virus-infected cattle are implicated in immune regulation and inflammation

**DOI:** 10.3389/fimmu.2026.1856022

**Published:** 2026-07-29

**Authors:** Cecilia Valeria Pérez, Hugo Adrián Carignano, Yamila Celeste Marchetti, Diego Daniel González, Vanesa Ruiz, Karina Gabriela Trono, Claudia Mongini

**Affiliations:** Laboratorio de Estudio de Vesículas Extracelulares y miRNA. Instituto de Virología e Innovaciones Tecnológicas (IVIT), Instituto Nacional de Tecnología Agropecuaria (INTA) - Consejo Nacional de Investigaciones Científicas y Técnicas (CONICET). Hurlingham, Buenos Aires, Argentina

**Keywords:** bovine leukemia virus, colostrum, extracellular vesicles, immune response, proteomics

## Abstract

**Introduction:**

Bovine leukemia virus (BLV) is an oncogenic retrovirus that naturally infects cattle. Once infected, the virus persists indefinitely throughout life at a transcriptionally silent stage. Soon after infection, humoral and cytotoxic activities efficiently abolish the viral replicative cycle, allowing only mitotic expansion of provirus-carrying cells. These anti-viral activities persist throughout the animal's life indicating that the immune system is permanently stimulated by BLV antigens. Although protein production is abolished during the chronic stage, viral transcription is not completely shut down. Extracellular vesicles (EVs) are nano-sized lipid bilayer vesicles released from most cells and play multiple roles in cell-to-cell communication, including immune modulation, angiogenesis, and transformation of cells by transferring genetic material and functional proteins. Colostrum is rich in EVs that carry bioactive proteins and RNAs reflecting maternal physiology and immune status. In this study, we aimed to characterize how BLV infection influences the protein cargo of colostrum-derived EVs.

**Methods:**

Colostral EVs were isolated using a protocol that combines acid pre-treatment with ultracentrifugation on sucrose gradients.

**Results:**

Transmission electron microscopy and nanoparticle tracking analysis revealed a heterogeneous population of vesicles ranging from 40 to 120 nm in diameter, exhibiting the characteristic morphology of EVs. Flow cytometry analysis confirmed the presence of the EV marker CD9. In addition, the presence of CD63, CD81, and TSG101 was also detected by Western blot. Quantitative proteomic analysis using a mass spectrometer coupled to liquid chromatography (LC/MS) revealed a total of 13 bovine differentially abundant proteins (FDR < 0.05 and FC ≥ |1.5|) from a total of 1,082 proteins identified. Among them, 9 (69.2%) showed decreased abundance (e,g., ANXA1, CLU, DMBT1, LRRC32), whereas 4 (30.8%) showed an increased abundance (e.g., IDO1, MARCKS, LCP1) in the group of BLV-infected animals. Enrichment analysis showed that differentially abundant proteins were primarily associated with immune-related processes, including complement activation and immunoglobulin-mediated immunity, as well as cellular metabolic processes, cytoskeletal organization, intracellular signaling and vesicle trafficking.

**Discussion:**

Overall, the results observed in colostrum-derived EVs from BLV-infected cows provide compelling evidence that BLV infection induced systemic molecular alterations that may modulate inflammation and the immune response in calves.

## 1 Introduction

Bovine leukemia virus (BLV), a deltaretrovirus that infects B cells, is the causative agent of the enzootic bovine leukosis (EBL), the most prevalent neoplastic disease in cattle worldwide. Infection is typically asymptomatic; however, approximately one-third of infected animals develop persistent lymphocytosis, and between 1% and 10% progress to leukemia or lymphosarcoma (1). Although most infected animals remain asymptomatic, they function as reservoirs for viral dissemination. While the transmission of BLV in adult cattle is primarily mediated by the transfer of infected lymphocytes via iatrogenic procedures, the role of colostrum and milk in natural transmission is also significant (2). Despite low levels of free virus in blood or milk, BLV is easily transmitted among cattle herds, suggesting additional routes of infection. While most infections show no symptoms, BLV causes significant economic and health impacts, particularly in countries such as Argentina, Brazil, Canada, and the United States, where prevalence is increasing (3, 4).

Extracellular vesicles (EVs) are nanosized particles secreted by most cell types, playing an essential role for intercellular communication, through the transfer of nucleic acids, proteins, and surface molecules (5–7). Because their cargo reflects the molecular composition of their cell of origin, EVs provide valuable insights into physiological and pathological processes. In recent years, their role in viral infections has garnered significant attention as EVs can encapsulate viral RNAs, proteins, and even whole virions, facilitating pathogen transmission while evading immune surveillance (8–10). The study of Szczotka et al. (11) provided evidence that EVs from plasma and serum of BLV-infected cattle express the viral proteins gp51 and p24 together with exosomal markers, including CD9, CD63, and flotillin-1. This finding confirms that EVs incorporate components of BLV and may serve as alternative mechanisms for viral dissemination, independent of the release of free virions.

Colostrum is essential for transferring passive immunity to neonatal calves. Compared to mature milk, colostrum-derived EVs are markedly enriched with immunomodulatory molecules exhibiting anti-inflammatory and barrier-protective properties within mammary tissues (12–17). Despite these benefits, colostrum can also facilitate the vertical transmission of BLV. Postnatal BLV transmission to calves occurs predominantly through the ingestion of infected colostrum and milk. While heat treatment and pasteurization of colostrum successfully reduce BLV infectivity and mitigate the risk of transmission in calves, the specific role of EVs as carriers of viral cargo remains insufficiently examined. The predominant focus of existing research has been on their application prospects as biomarkers for early diagnosis and on their therapeutic potential in mitigating bovine mastitis (18).

Furthermore, Rahman et al. (19) demonstrated a distinct proteome in milk EVs from BLV-infected cows, reinforcing the hypothesis that colostrum EVs may act as carriers of viral components (e.g., proteins, RNA species) or infection-associated host factors that can be internalized by neonatal target cells, thereby facilitating viral dissemination or priming a permissive cellular environment. Colostral EVs may also influence the development of the neonatal immune system by transferring immunomodulatory molecules, including miRNAs and proteins, that can shape immune maturation, tolerance, or susceptibility to infection. This dual role suggests that EVs are not only passive biomarkers but also active mediators of host–virus interactions, potentially affecting both BLV transmission dynamics and long-term physiological and immunological outcomes in offspring. Elucidating the role of BLV-derived EVs in colostrum in maternal-to-offspring transmission may reveal novel mechanisms that influence neonatal infection, immune imprinting, and herd-level dissemination patterns. In this context, the present study aimed to investigate the impact of BLV infection on the protein cargo composition of colostrum-derived EVs, with a particular focus on their potential role in host interactions during early life.

## 2 Materials and methods

### 2.1 Animals

Eight adult cows from the Centro de Investigaciones en Ciencias Veterinarias y Agronómicas (CICVyA), Instituto Nacional de Tecnología Agropecuaria (INTA) (Buenos Aires, Argentina) were included in this study. Their BLV status was assessed by detecting BLV genomic DNA (pol) by qPCR and BLV-specific antibodies by ELISA. All the animals used in this study were housed in a loose-housing yard with free stalls at an experimental facility belonging to the CICVyA-INTA. Animals were fed using an unmixed feeding system in which concentrates were given separately from forages. The veterinary staff periodically examined the animals. All experimental protocols, including procedures for animal handling and sampling, were approved by the Institutional Animal Care and Use Committee of the CNIA-INTA. The Institutional Manual’s guidelines were rigorously adhered to throughout the study.

Blood was collected from the jugular vein using EDTA solution (225 μM) as an anticoagulant. After centrifuging at 1, 500 x g for 25 minutes, the buffy coat was used for genomic DNA extraction, and plasma fractions were used to determine anti-BLV antibodies by ELISA.

### 2.2 Detection of BLV antibodies

Plasma BLV-specific antibodies were measured on the day of parturition and two months later using an in-house ELISA, as previously described (20). Briefly, Nunc Polysorp plates were coated with 0.5 µg/ml recombinant BLV p24 capsid protein, blocked with PBSTE (PBS supplemented with 10% equine serum and 0.2% Tween-20), and incubated with plasma samples diluted 1:25 in duplicate. Bound antibodies were detected using a peroxidase-conjugated goat anti-bovine IgG (KPL) and TMB substrate (Sigma). Each plate included strong-positive, weak-positive, and negative control sera. The assay was considered valid when the absorbance difference between the weak-positive and negative controls was at least 0.35. Results were normalized by calculating a sample-to-positive (S/P) ratio based on the mean optical density of replicate wells. The weak-positive control was assigned a value of 100%, and all sample readings were expressed relative to it. A 25% cut-off was used to define positivity (20).

### 2.3 Determination of BLV proviral load

Total genomic DNA was extracted from buffy coat samples using the High Pure PCR Template Preparation Kit (Roche). DNA concentration and purity were determined by spectrophotometry with a Nanophotometer (NanoDrop, Thermo Fisher Scientific). Proviral load was quantified by real-time SYBR Green qPCR assay developed in our laboratory targeting the pol gene (21, 22). For each reaction, a 25 µl mixture was prepared containing FastStart Universal SYBR Green Master Mix (Roche), 800 nM forward and reverse primers (BLVpol5f: 5’–CCTCAATTCCCTTTAAACTA-3’ and BLVpol3r: 5’–GTACCGGGAAGACTGGATTA–3’), and 300–500 ng of DNA template. The amplification protocol included an initial incubation at 50 °C for 2 minutes and 95 °C for 10 minutes, followed by 40 cycles at 95 °C for 15 seconds, 55 °C for 15 seconds, and 60 °C for 1 minute. Samples were run in duplicate on a StepOne Plus instrument (Applied Biosystems). Each plate contained positive and negative controls, as well as a no-template control. The specificity of each BLV-positive reaction was confirmed with the melting temperature dissociation curve (Tm) analysis. Quantification was carried out using the plasmid pBLV1 (NVRI, Pulawy, Poland), which contains a segment of the BLV pol gene. Serial tenfold dilutions ranging from 5 x 10^6^ to 5 copies/µl were employed to construct the standard curve and determine BLV copy numbers. Values > 1, 500 copies per μg of total DNA (copies/μg) were considered as high proviral load (PVL) (23).

### 2.4 EVs isolation

Bovine colostrum was collected from BLV-infected [BLV(+)] and uninfected [BLV (–)] cows within 24 hours post-calving, transported to the laboratory, and stored at −20 °C. IgG content was determined indirectly by a colostrometer (specific gravity) and a Brix refractometer (total solids). Colostrum small EVs were isolated and purified as previously described with several modifications (24, 25). Briefly, colostrum samples were first centrifuged at 5, 000 x g for 30 minutes at 4 °C to remove the fat layer, large debris, and mammary gland-derived cells. Casein micelles were eliminated by acid precipitation (acetic acid, pH 4.6), followed by centrifugation at 5, 000 x g. The supernatant was then diluted 1:3 in PBS and subjected to sequential centrifugations at 35, 000 x g and 70, 000 x g (1 hour each, 4 °C), followed by stepwise filtration (0.8, 0.65, 0.45, and 0.22 μm). EVs were pelleted by ultracentrifugation at 100, 000 g for 1 hour and resuspended in PBS. Further purification was performed by discontinuous sucrose density gradient ultracentrifugation (150, 000 x g, 16 hours, 4 °C, SW-32 Ti rotor, Beckman Coulter). Fractions with densities of 1.15–1.19 g/ml were pooled, washed at 100, 000 x g for 60 minutes in PBS, and resuspended in 0.22 μm-filtered PBS.

### 2.5 Transmission electron microscopy

A drop of 5-10 μl of EVs suspension was placed on Formvar-coated electron microscopic grids. Subsequently, the EVs were stained with 2% uranyl acetate. Their morphology was examined under an electron microscope, EM 109T (Zeiss), equipped with a digital camera (Gatan ES1000W).

### 2.6 Nanoparticle tracking analysis

The size distribution of EVs was assessed using the ZetaView^®^ system from Particle Metrix (ZetaView PMX-230 Twin). Videography of the particles’ dynamic movement was recorded and subsequently analyzed using ZetaView software (version 8.06.01 SP1).

### 2.7 Flow cytometry

EVs (25 μl) were incubated with 10 μl of aldehyde/sulfate latex beads (Invitrogen, 4% w/v 4 μm diameter). After saturation with 100 mM glycine, EVs were incubated with a monoclonal antibody against CD9 conjugated with biotin (BioRad, MCA469B), followed by streptavidin-phycoerythrin (PE) conjugate (eBioscience). Analysis was conducted on the singlet gate of a forward scatter versus side scatter dot plot (26, 27). Fluorescence was measured in a BD FACSCalibur flow cytometer (BD Biosciences). The resulting data were analyzed utilizing FlowJo X software.

### 2.8 Western blotting

A standard Bradford assay was used to determine the total protein concentration of EVs samples. For tetraspanin detection (CD9, CD63, CD81), EVs proteins (15 μg) were diluted in non-reducing SDS sample buffer at room temperature. For the detection of BLV proteins and TSG101, 15 μg of EVs proteins were diluted in SDS-reducing buffer and boiled before loading onto the gel. Proteins were resolved by 12% SDS-PAGE and transferred onto PVDF membranes (Amersham Hybond P, Cytiva). Membranes were blocked with 5% non-fat dry milk in TBS containing 0.1% Tween-20 and probed overnight with the following primary antibodies: biotinylated anti-CD9 (1:100, MCA469B, Bio-Rad), biotinylated anti-CD63 (1:100, 130-100-169, Miltenyi Biotec), biotinylated anti-CD81 (1:100, 104903, BioLegend), anti-TSG101 (1:100, sc-7964, Santa Cruz), or anti-BLV polyclonal antibody (1:100, Porta 2018) (28). Secondary detection was performed with avidin-HRP, anti-mouse HRP, or anti-chicken HRP (all 1:3000). Proteins were visualized by enhanced chemiluminescence and images were captured with GeneSys software (1.5.7.0 version) from SynGene (Synoptics Ltd).

### 2.9 Proteomic analysis

#### 2.9.1 Sample preparation

EVs isolated from colostrum samples obtained from BLV(+) and BLV(–) cows were incubated at 90 °C for 5 minutes in the presence of reducing loading buffer. Subsequently, subjected to SDS-PAGE under denaturing conditions (**Supplementary Figure 1**). A total of 30 µg of protein was loaded per sample, and electrophoresis was stopped when the entire sample had concentrated to approximately 1 cm^2^ on the gel.

#### 2.9.2 Liquid chromatography coupled to mass spectrometry analysis

Mass spectrometric analysis was conducted at the Mass Spectrometry Unit of the Institute of Molecular and Cellular Biology of Rosario (UEM-IBR), Argentina. The gel fragments were processed following the protocol described by Link and LaBaer (29). The protein-purified fractions were then processed and analyzed to determine their protein abundance profiles. 4 µl of peptides from each sample were injected into a Q-Exactive HF mass spectrometer (Thermo Fisher Scientific) operating in positive ion mode. Peptide separation was carried out on nanoHPLC Ultimate3000 (Thermo Scientific) using a nano column EASY- Spray ES903 (50 cm x 50 μm ID, PepMap RSLC C18). The mobile phase flow rate was 300 nL/minute using 0.1% formic acid in water (solvent A) and 0.1% formic acid in acetonitrile (solvent B). The chromatographic profile was as follows: 5–35% solvent B over 90 minutes, then 35–90% solvent B over 20 minutes, followed by 90% solvent B for 5 minutes. MS analysis was performed using a Q-Exactive HF mass spectrometer (Thermo Scientific). For ionization, a liquid junction voltage of 1, 9 kV and a capillary temperature of 250 °C were used. The full scan method employed a m/z 375–2, 000 mass selection, an Orbitrap resolution of 120, 000 (at m/z 200), a target automatic gain control (AGC) value of 1e6, and a maximum injection time of 100 ms. After the survey scan, the 8 most intense precursor ions were selected for MS/MS fragmentation. Fragmentation was performed with a normalized collision energy of 27 eV, and MS/MS scans were acquired with a dynamic first mass. The AGC target was 5e5, resolution of 30, 000 (at m/z 200), an intensity threshold of 1.5e5, an isolation window of 1.4 m/z, and a maximum injection time of 55 ms. Charge-state screening was enabled to reject unassigned, singly charged, and protonated ions with charge states of 6 or higher. A dynamic exclusion time of 30 s was used to discriminate against previously selected ions.

#### 2.9.3 Mass spectrometry data analysis

Mass Spectrometry (MS) data were analyzed using Proteome Discoverer (version 2.4.1.15) (30) with standardized workflows. Raw mass spectra data files were searched against UniProt proteome databases (https://www.uniprot.org/) for Bos taurus (acc number UP000009136; 59, 264 proteins associated with 26, 942 genes) and Bovine Leukemia Virus (BLV) (acc number UP000202838; 11 poly/proteins sequences associated with 7 genes), respectively. Precursor and fragment mass tolerances were set to 10 ppm and 0.02 Da, respectively, with a maximum of 2 missed cleavages. The following modifications were set: i) Dynamic modifications: maximum equal modifications per peptide: 3 maximum dynamic modifications per peptide: 4, oxidation: +15.995 Da (M); ii) Dynamic modifications (protein terminus): N-terminal modification: Acetyl +42.011 Da (N-Terminus), Met-loss: -131.040 Da (M), Met-loss+Acetyl: -89.030 Da (M); static modifications: Carbamidomethyl: +57.021 Da (C). Protein quantification was based on label-free quantification (LFQ). To ensure an accurate comparison of protein abundance across samples, LFQ intensities were normalized to correct for technical variability, including differences in sample loading, ionization efficiency, and instrument performance. Quality control (QC) filtering excluded proteins that were not detected in at least three out of the eight total samples, or were identified by fewer than two unique peptides. Any missing abundance values were imputed as zeros across all samples.

#### 2.9.4 Differential protein abundance analysis

An exploratory analysis of protein abundance variation patterns between BLV(+) and BLV(–) cows was conducted using principal component analysis (PCA). To ensure homoscedasticity of the data, the raw protein abundances per sample were normalized employing the trimmed mean of M-values (TMM) method, implemented in edgeR v3.32.1 (31). Pairwise distances among samples were calculated based on the root-mean-square deviation of the top protein exhibiting the largest variances in log2-fold-change (Log2FC) between each pair of samples.

Differentially abundant proteins (DAPs) between the BLV(+) and BLV(–) groups were identified using the edgeR package (32, 33). Protein-wise dispersion of normalized protein abundance was estimated using a negative binomial generalized linear model (GLM) (34). Statistical significance was evaluated using a likelihood ratio test (LRT). Multiple comparisons correction for the p-values was done by the Benjamini-Hochberg false discovery rate (FDR) method (35). Proteins with adjusted p-values (q-values) < 0.05 and Log2FC > |1.5| were considered statistically significant. The PCA and Volcano plots were generated using the R graphics environment (R Core Team, 2024).

#### 2.9.5 Protein set enrichment analysis

The functional annotation of the identified proteins was carried out using the PANTHER classification system v19.0 (http://www.pantherdb.org/) (36, 37) with Gene Ontology (GO) terms (Biological Process (BP), Molecular Function (MF) and Cellular Component (CC)), Phanter Protein Class (PPC) and Reactome pathways. Protein set enrichment analysis was performed using the ranked list of proteins by log2FC as input to the PANTHER classification system v19.0 (37). Enrichment of GO categories, PPC, and Reactome pathways was evaluated using a non-parametric Mann–Whitney U test, which determines whether functional categories are enriched among the most up- or downregulated proteins in the ranked list. A GO BP category–protein graph was generated to visualize the links between significantly enriched BP GO terms and their associated proteins using the R graphical environment (R Core Team, 2024).

### 2.10 Statistical analysis

Results of NTA and flow cytometry analyses are expressed as mean ± SEM. Comparisons between groups were assessed by the nonparametric Mann–Whitney U test. P-values <0.05 were considered significant.

## 3 Results

### 3.1 Characterization of cattle according to BLV infection status

A total of eight cows were included in this study based on their BLV infection status, determined by anti-BLV antibodies and BLV proviral load. Four animals were classified as BLV-infected [BLV(+)], showing detectable anti-BLV antibodies and proviral load, whereas four animals were confirmed as uninfected [BLV(−)]. Among BLV(+) animals, three exhibited a high proviral load (>1, 500 copies/µg of total DNA), while one animal presented a low proviral load. Anti-BLV antibodies were consistently detected in BLV(+) animals at both evaluated time points (calving and two months postpartum), whereas BLV(−) animals remained seronegative throughout the study. All colostrum samples met the criteria for high quality, with Brix values exceeding 22% and density values above 1.050 g/mL, indicating comparable sample quality across groups. The evaluated parameters are summarized in **Table 1**.

**Table 1:** Assessment of BLV infection.

Animal ID	Age^1^ (month)	ELISA^2^	PVL^3^	Density	% brix
332	96	+	<5	1.065	22
379	71	+	40, 979	1.067	NDet
383	60	+	39, 146	>1.075	NDet
393	72	+	34, 358	>1.075	28
423	60	–	ND	>1.075	31
475	36	–	ND	1.050	17
363	71	–	ND	>1.075	NDet
386	60	–	ND	1.057	22

+, positive; -, negative; ND, not detected, NDet, not determined; ^1^Age at calving; ^2^ELISA, anti-BLV antibody enzyme-linked immunosorbent assay; ^3^PVL, BLV proviral DNA measured by qPCR (copies per μg of DNA).

### 3.2 Characterization of colostral EVs

Colostral EVs isolated from BLV(+) and BLV(–) cows were biophysically characterized through NTA, TEM, flow cytometry, and western blot in accordance with the guidelines established by the International Society for Extracellular Vesicles (ISEV) in *The Minimal Information for Studies of Extracellular Vesicles* (MISEV2023) (38).

**Figure 1:**
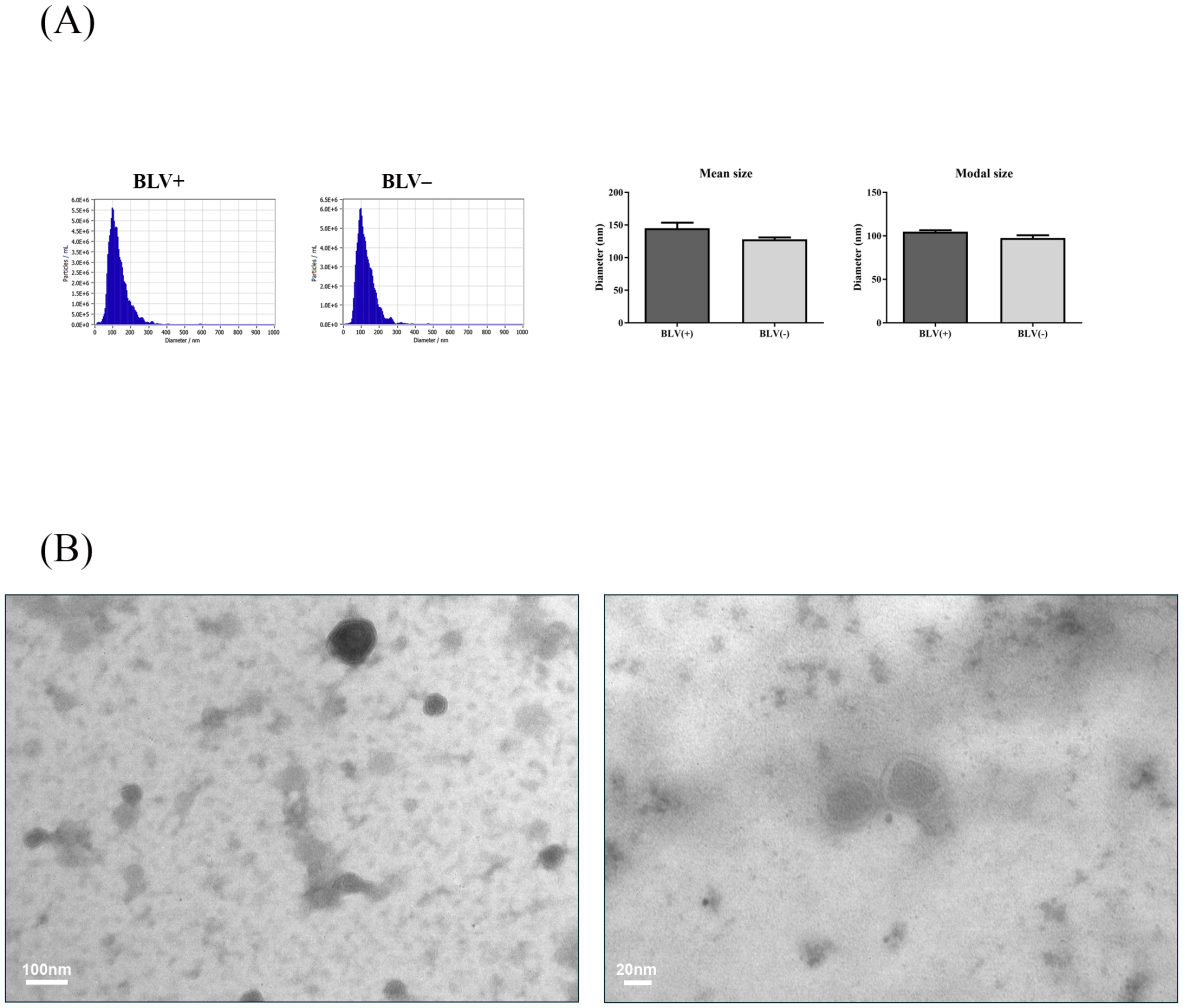
Biophysical characterization of colostral EVs from BLV(+) and BLV(–) cows. **(A)** Representative NTA profiles of colostral EVs isolated from BLV(+) and BLV(–) cows. Column bar graphs represent the modal or mean values ± SEM. The nonparametric Mann-Whitney U test was used for statistical analysis. **(B)** Representative TEM images of colostral EVs displaying their size and spherical bilayer morphology.

**Figure 2:**
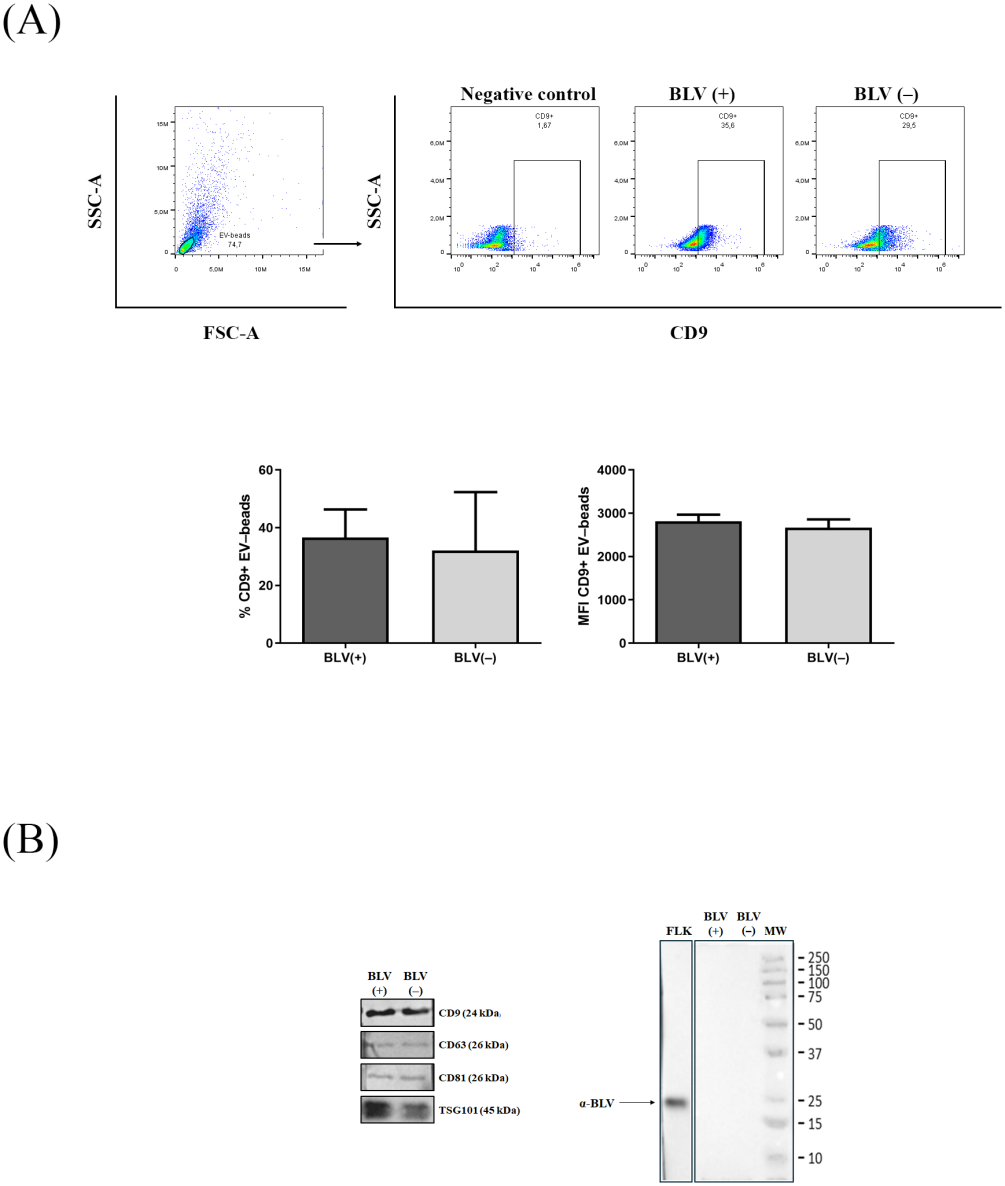
EV markers expression on colostral EVs. **(A)** FACS analysis of colostral EVs from BLV(+) and BLV(–) cows. EVs were coupled with aldehyde/sulfate latex beads of 4 μm diameter and incubated with anti-CD9 antibody, followed by PE-conjugated streptavidin. Representative dot plots showing the percentage of CD9+ EVs. Negative control: omission of primary antibody. The analysis was performed in the singlet region determined from the SSC vs FSC light scatter plot. Quantitative assessment of the percentage (%) of CD9+ EVs from BLV(+) and BLV(–) cows. The mean value ± SEM of the Median Fluorescence Intensity (MFI) is also shown. The nonparametric Mann-Whitney U test was used for statistical analysis. **(B)** Western blot analysis of colostral EVs from BLV(+) and BLV(–) cows. Western blot analysis revalidating the expression of CD9 on colostral EVs. CD63, CD81 and TSG101 are also expressed. No viral proteins were detected within colostral EVs. FLK: EVs concentrated from the supernatant of permanently BLV–infected foetal lamb kidney cell culture; MW: molecular weight marker.

NTA analysis showed a right-skewed size distribution in both BLV(+) and BLV(–) groups. The modal particle diameter was 104.8 nm for BLV(+) cows and 97.5 nm for BLV(–) cows, while the mean particle sizes were 144.9 ± 8.7 nm and 128.0 ± 2.9 nm, respectively. Most particles were smaller than 200 nm, consistent with a population enriched in small EVs. No significant differences in particle size were observed between the two groups (**Figure 1A**). TEM analysis revealed that EVs derived from colostrum of both BLV(+) and BLV(–) cows are heterogeneous and displayed a similar spherical bilayer morphology (**Figure 1B**). Flow cytometry analyses demonstrated that EVs derived from BLV(+) and BLV(–) cows expressed the tetraspanin marker CD9 at similar percentages and mean fluorescence intensities, with no statistically significant differences between groups (**Figure 2A**). Western blotting analysis confirmed the presence of the EV marker CD9 in colostrum-derived EVs from both groups. In addition, the expression of CD63, CD81, and TSG101 was also detected by western blotting in colostral EVs derived from BLV(+) and BLV(–) cows (**Figure 2B**). In contrast, BLV proteins were not detected in colostral EV preparations from either BLV(+) or BLV(−) animals (**Figure 2B**), suggesting that viral components are not associated with colostral EVs under the conditions analyzed.

### 3.3 Proteomic profiling of colostral EVs

**Figure 3:**
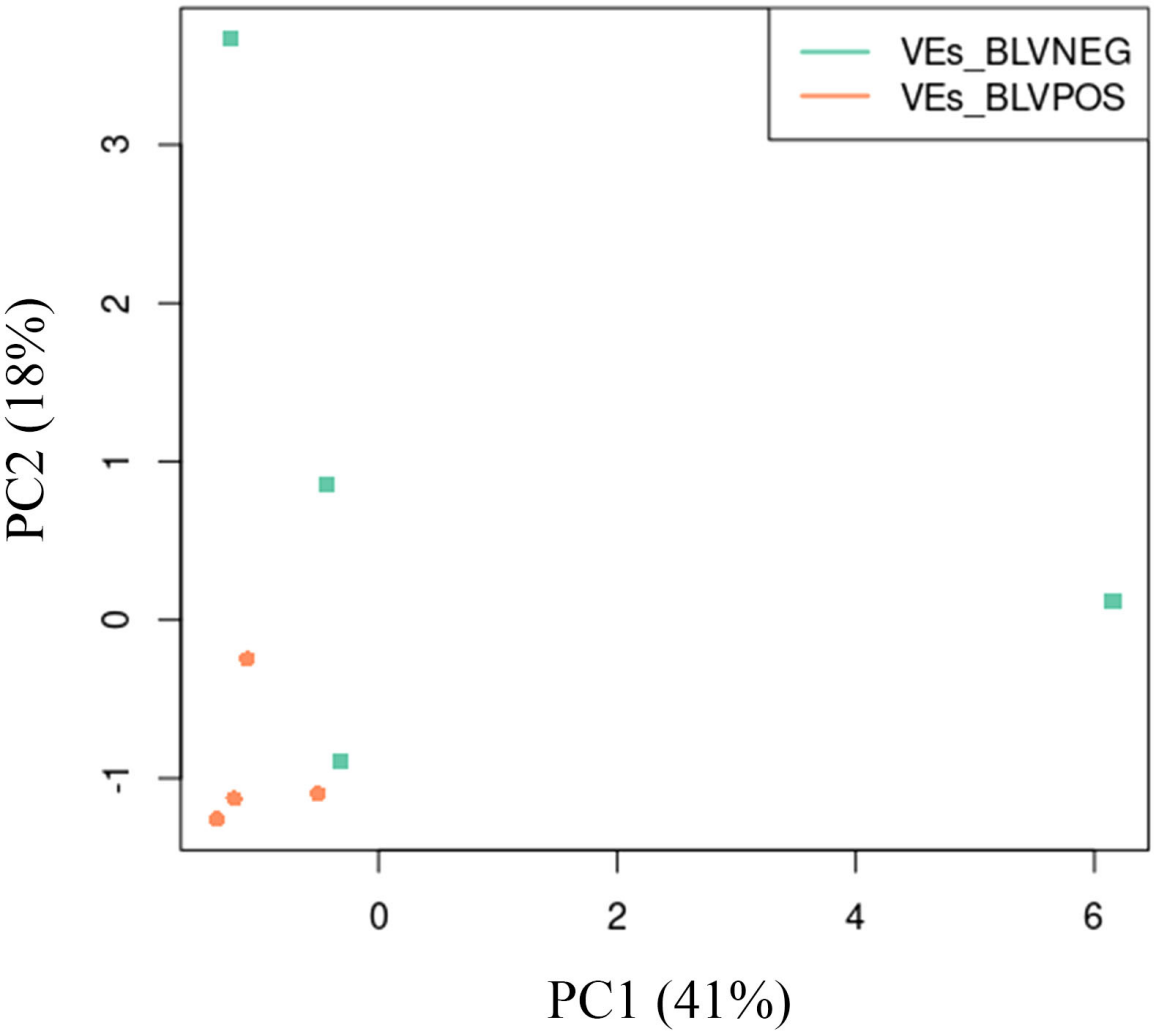
Principal component (PC) analysis of the protein abundance matrix. Red circles and green squares represent BLV(+) and BLV(−) samples, respectively. PC, principal component. PC1 and PC2 account for 41% and 18% of the variance in the abundance data, respectively.

Isolated EVs were analyzed using a nano LC–MS/MS method to compare the proteins of BLV(+) EVs with those of BLV(–) EVs. A similar number of proteins was identified and quantified across all samples (ranging from 1, 222 to 1, 393). After QC, the protein abundance matrix included a total of 1, 082 proteins per sample (**Supplementary Table 1**). Among these, several EVs-associated proteins were detected at comparable expression levels, including the tetraspanins CD9, CD63, and CD81, as well as TSG101 and ALIX. In addition, proteins linked to vesicle biogenesis and trafficking were identified, such as ESCRT-related components (e.g., members of the VPS37 family) and Rab GTPases. Moreover, molecular chaperones frequently found in EVs were detected, including representatives of the HSP70 and HSP90 families, together with other exosome-associated markers such as LAMP2. As shown in **Figure 3**, PC1 accounted for 41% of the total variance, while PC2 explained 18%. The individuals from the BLV(+) group clustered together (red circles), whereas the BLV(–) group showed greater dispersion (green squares).

**Figure 4:**
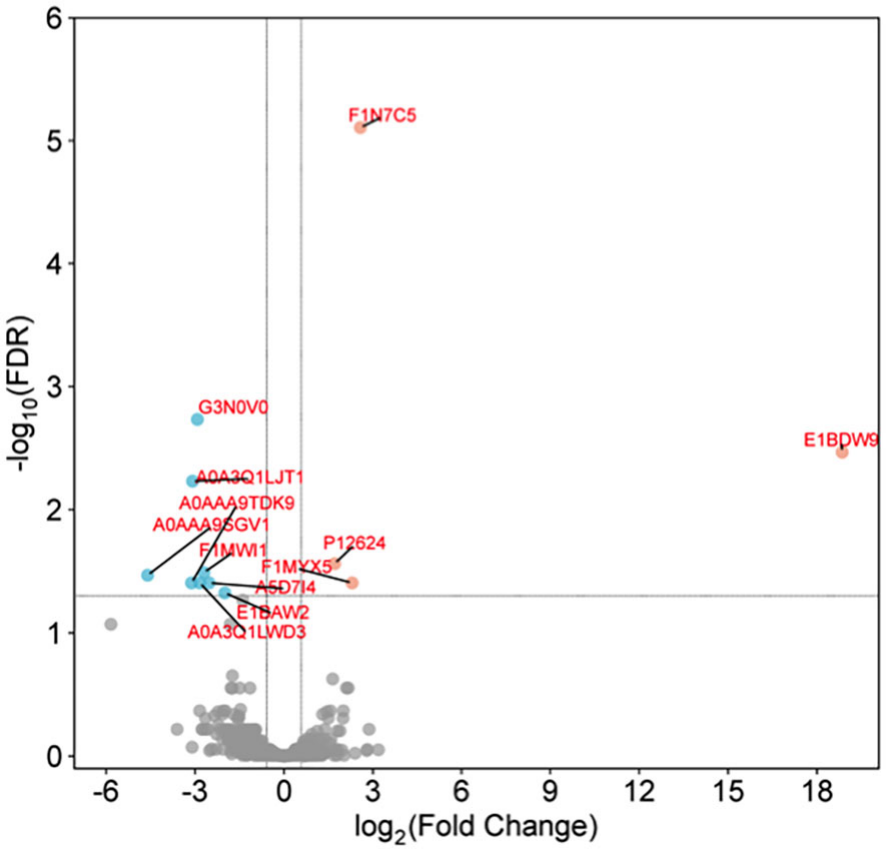
Volcano plot. Differentially abundant proteins between BLV(+) and BLV(–). Vertical lines represent log2(FC) thresholds of −1.5 and 1.5. Light blue circles indicate DAPs, while gray circles represent non-significant proteins and/or those with fold change values below |1.5|. UniProt accession label proteins with FDR < 0.05.

Differential abundance analysis was performed using a generalized linear model (GLM). A total of 13 significant differentially abundant proteins (DAPs) were identified (**Supplementary Table 2**) between BLV(+) and BLV(–) groups: ANXA1 (acc. A0AAA9THP8); IDO1 (acc. F1N7C5); EXOC2 (acc. E1BDW9); Ig-like (acc. A0A3Q1LJT1); Ig-like (acc. G3N0V0); MARCKS (acc. P12624); CLU (acc. F1MWI1); NID1 (acc. A0AAA9SGV1); LCP1 (acc. F1MYX5); EXT1 (acc. A5D7I4); ARSA (acc. A0AAA9TDK9); DMBT1 (acc. A0A3Q1LWD3); and LRRC32 (acc. E1BAW2). These DAPs were identified from a total of 1, 082 proteins evaluated (FDR < 0.05 and FC ≥ |1.5|) (**Figure 4**). Among them, 69.2% (9 proteins) were downregulated (DProt), and 30.8% (4 proteins) were upregulated (UProt) in the BLV(+) group.

### 3.4 Functional enrichment analysis of colostral EV proteins

**Figure 5:**
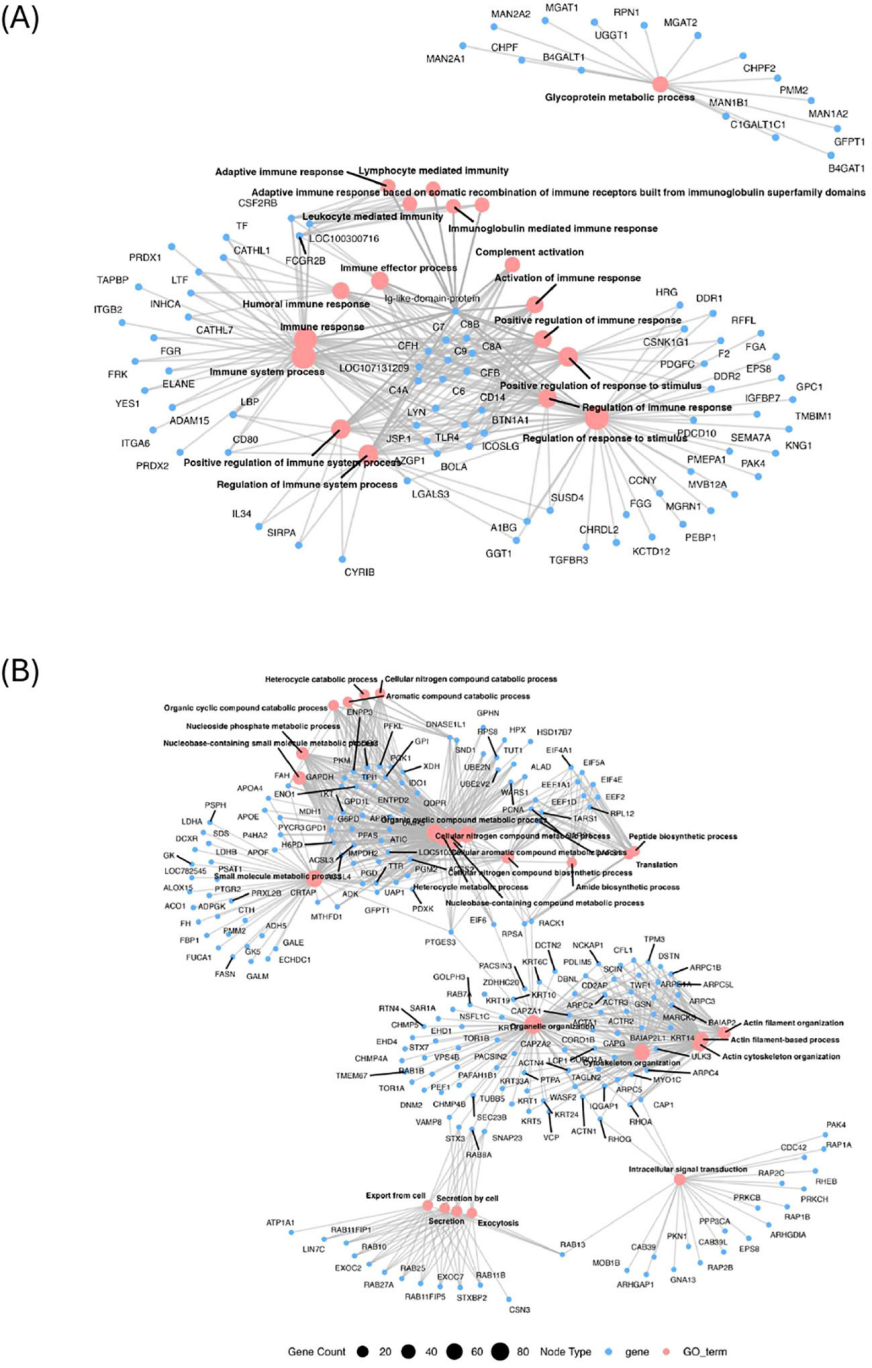
Gene set enrichment graph plot for GO BP terms. Each graph shows the relationship between the protein set enriched for BP GO terms (light pink node) and its associated proteins (light blue nodes). Node size reflects the number of proteins associated with each GO term (Count), and edges indicate protein-GO term associations. Proteins in each graph include downregulated proteins **(A)** and upregulated proteins **(B)**.

To gain biological insight into the phenotypes studied, a protein-set enrichment analysis was performed. Identified proteins were ranked according to their log2FC values between conditions. GO terms were used to identify functional categories enriched at the extremes of the ranked list, corresponding to the most UProt and most DProt proteins (**Supplementary Table 3**). Several GO-slim BP terms significantly enriched (q-value < 0.05) in DProt were related to immune response activation and regulation, lymphocyte- and immunoglobulin-mediated immunity, and complement activation (for example, “Immune response” (GO:0006955), “Humoral immune response” (GO:0006959), and “Complement activation” (GO:0006956)) (**Figure 5A**). In contrast, the UProt enriched terms were mainly associated with signal transduction, cytoskeleton organization, cellular metabolic processes (nitrogen- and aromatic- compound metabolism) and exocytosis (for example, “Cellular nitrogen compound metabolic process” (GO:0034641), “Nucleobase-containing compound metabolic process” (GO:0006139), “Actin cytoskeleton organization” (GO:0030036), “Actin filament-based process” (GO:0030029) “Exocytosis” (GO:0006887), “Intracellular signal transduction” (GO:0035556) and others) (**Figure 5B**). Additionally, Protein Class (PC) terms enriched in Uprot included “Actin or Actin-binding cytoskeletal protein” (PC00041) and “Heterotrimeric G-protein” (PC00117), whereas PC terms related to DProt were “Complement component” (PC00078), “Defense/immunity protein” (PC00090), “Glycosyltransferase” (PC00111), “Protease inhibitor” (PC00191), “Immunoglobulin” (PC00123), among others. Also, Reactome biological pathways were enriched in DProt and UProt. Notably, pathways related to complement cascade activation and modulation, indicating inhibition of innate immune defense in BLV(+) cows. Besides, Reactome functional enrichment in UProt suggests an active cellular state characterized by RNA metabolism and protein synthesis (e.g., “Metabolism of RNA” (R-BTA-8953854), “Translation” (R-BTA-72766). Simultaneously, the involvement of Rho GTPase signaling -including Miro GTPases and RHOBTB3- (R-BTA-72766) implies tight regulation of cytoskeletal organization, vesicular trafficking, and cellular dynamics, essential for coordinating mitosis and maintaining intracellular architecture.

## 4 Discussion

This study characterizes EVs isolated from the colostrum of both BLV-infected and uninfected cows and analyzes their protein cargo using high-throughput proteomic methods. While the biophysical properties of colostral EVs were comparable across groups, BLV infection was associated with changes in vesicular protein composition. These findings imply that viral infection does not substantially impact EV production or morphology in colostrum but may affect the molecular signals contained within EVs.

Colostrum is essential for neonatal calf immunity, providing antibodies and a wide range of bioactive molecules that promote early immune development. In addition to soluble components, EVs have emerged as important mediators of intercellular communication and may participate in the transfer of immunomodulatory signals from the dam to the neonate. For instance, Hata et al. (39) demonstrated that EVs isolated from bovine colostrum contain miRNAs associated with immune regulation, highlighting their potential to modulate immune responses in newborns. In addition, Samuel et al. (12) have shown that bovine colostrum-derived exosomes contain a rich repertoire of proteins involved not only in immune regulation but also in growth, repair, and development of the infant. Therefore, alterations in the protein cargo of colostral EVs associated with BLV infection may reflect changes in the maternal immune environment and potentially influence the biological information delivered to the newborn calf.

The aim of this study was to investigate the influence of BLV infection on the protein cargo of colostral EVs. Small EVs were isolated using a protocol that combines acid pre-treatment with ultracentrifugation on sucrose gradients. TEM and NTA revealed a heterogeneous population of vesicles ranging from 40 to 120 nm in diameter, exhibiting the characteristic morphology of EVs. The observed differences in vesicle size between NTA and TEM may result from methodological variation inherent to these techniques. Methods based on particle mobility, such as NTA, measure the hydrodynamic diameter of particles, which may lead to an overestimation of vesicle size compared to imaging-based techniques like TEM (38). The presence of canonical EV markers was initially confirmed through flow cytometry and/or western blotting, demonstrating the expression of CD9, CD63, CD81, and TSG101. Proteomic analysis further corroborates these findings by confirming the presence of these markers and identifying additional EV-associated proteins, including ALIX, as well as proteins involved in vesicle biogenesis and trafficking, such as ESCRT-related proteins (e.g., VPS37 family members) and Rab family proteins. Moreover, several molecular chaperones commonly enriched in EVs were identified, including members of the HSP70 and HSP90 families, as well as other EV-associated proteins such as LAMP2. Additionally, the absence of cellular contamination markers such as ACTN4, Histone H3, and Cytochrome c (**Supplementary Table 1**) further supports the purity of the EV preparations. These proteins are typically associated with cytoskeletal, nuclear, and mitochondrial compartments, respectively, and their absence indicates minimal contamination with cellular debris or intracellular components. Collectively, the detection of EV-associated markers, coupled with morphological and biophysical characterization, supports the conclusion that the isolated particles are *bona fide* exosomes.

Quantitative proteomic analysis revealed that no proteins were uniquely detected between the BLV(+) and BLV (–) groups; however, differences were evident in relative abundance. Furthermore, no viral proteins were identified in any sample. Although the BLV-positive group included three cows with high PVL and one cow with low PVL, PCA revealed a clear separation between infected and non-infected animals. Moreover, the use of four biological replicates per group is consistent with current recommendations for quantitative proteomic studies (40). Future studies including more animals and stratification according to PVL levels will be necessary to further investigate the relationship between viral burden and proteomic alterations.

Functional protein-set enrichment analysis indicated that Dprot proteins in colostral EVs from BLV-infected cows are primarily associated with immune-related processes, including complement activation and immunoglobulin-mediated immunity. These findings indicate a decrease in immune-related components within the EV fraction of colostrum from infected animals. Since complement proteins and immunoglobulins are central components of innate and adaptive immunity, their reduced presence in EVs may reflect virus-associated modulation of immune pathways in the mammary gland, as well as in immune cells within the mammary tissue and in milk secretions. Conversely, proteins enriched in EVs from BLV-infected cows are mainly associated with cellular metabolic processes, cytoskeletal organization, intracellular signaling, and exocytosis. These pathways are critically connected to cellular activation and vesicular trafficking. In particular, the enrichment of proteins involved in actin cytoskeletal dynamics and Rho GTPase signaling suggests active regulation of intracellular architecture and vesicle transport mechanisms, which are essential for coordinating membrane trafficking and secretion (41). Notably, BLV proteins were not detected in colostral EVs by either Western blotting or untargeted proteomics. Although we cannot completely rule out that some viral proteins may be present below the detection limits of our analytical workflow, this observation is consistent with findings reported by Rahman et al. (19), who similarly found no viral proteins or genomic RNA in milk-derived EVs from BLV-infected cattle. Importantly, the presence of viral proteins in EVs appears to be compartment-dependent; while Szczotka et al. (11) detected gp51 and p24 in EVs derived from sera and plasma, mammary secretion-derived EVs may not package viral proteins to detectable levels under natural infection conditions. Thus, the alterations observed in the EV proteome may represent indirect effects of BLV infection on immune and epithelial cells that contribute to the colostrum vesicular fraction.

Among DAPs identified in colostral EVs, Annexin A1 (ANXA1) exhibited the largest reduction in abundance in EVs from BLV-infected cows compared with uninfected cows. ANXA1 is a membrane-associated protein involved in phospholipid binding, membrane trafficking, and cytoskeletal organization, and it has been reported in several EV populations (42, 43). In addition to its structural roles in membrane dynamics, ANXA1 regulates inflammatory responses and immune signaling. It contributes to the resolution of inflammation by limiting neutrophil recruitment, promoting neutrophil apoptosis, enhancing macrophage-mediated clearance of apoptotic cells, and supporting macrophage reprogramming toward a pro-resolving phenotype (44). In contrast, Indoleamine 2, 3-dioxygenase 1 (IDO1), an enzyme involved in the catabolism of tryptophan along the kynurenine pathway, showed the highest increase in abundance in EVs from BLV-infected cows. IDO1 is a key immunoregulatory molecule that modulates T cell responses and promotes immune tolerance through local depletion of tryptophan and the production of immunosuppressive metabolites (45). The presence of IDO1 within EVs suggests that these vesicles may contribute to the transfer of immunomodulatory signals between cells. These findings collectively indicate that infection with BLV is associated with a shift in the protein cargo of colostral EVs, favoring a more immunoregulatory profile. This profile is characterized by decreased abundance of pro-resolving mediators, such as ANXA1, and increased levels of immunosuppressive molecules, such as IDO1. Considering the role of colostral EVs in maternal–neonatal communication, these modifications may potentially influence the immune signals transferred to the calf during early life.

Beyond ANXA1 and IDO1, other DAPs identified in this study may also contribute to the immunological alterations observed in colostral EVs from BLV-infected cows. The upregulation of EXOC2, a core component of the exocyst complex involved in vesicular trafficking and exocytosis, suggests that BLV infection may exploit host secretory machinery to enhance viral budding and cell-to-cell transmission, potentially contributing to increased viral dissemination in infected dams (46, 47). Among the downregulated proteins, DMBT1, a pattern recognition receptor involved in pathogen neutralization and mucosal defense, may play a particularly relevant role in neonatal protection. Its reduced abundance in colostral EVs from BLV-infected cows could compromise the transfer of innate mucosal immunity to the calf, increasing susceptibility to enteric pathogens during the early postnatal period (48, 49). Similarly, the downregulation of LRRC32, a membrane protein that activates latent TGF-β and supports regulatory T cell function, suggests potential disruption of immune tolerance mechanisms in the neonate (50). Finally, the upregulation of MARCKS, a substrate of protein kinase C involved in neutrophil function and inflammatory signaling, may reflect dysregulated inflammatory responses in the mammary gland of BLV-infected cows (51, 52). Collectively, these findings suggest that BLV infection induces a broad remodeling of the colostral EV proteome that extends beyond individual proteins, affecting multiple interconnected immune pathways with potential consequences for neonatal immune development.

Several studies have demonstrated that EVs present in milk and colostrum actively participate in host–virus interactions and immune defense mechanism. EVs isolated from human colostrum have been demonstrated to exert antiviral effects against human cytomegalovirus (HCMV), by blocking viral attachment to cells and reducing infectivity *in vitro* (53). Similarly, human colostrum-derived EVs have been reported to inhibit infections caused by human rotavirus and respiratory syncytial virus *in vitro*, by interfering with the initial stages of the viral replicative cycle (54). Likewise, Näslund et al. (55) reported that exosomes isolated from human breast milk inhibit HIV-1 infection of dendritic cells and subsequent viral transfer to CD4+ T cells. In animal models, milk-derived EVs demonstrated antiviral effects, as exemplified by their capacity to inhibit Porcine Epidemic Diarrhea Virus (PEDV) infection and replication *in vitro* via vesicle-associated miRNAs (56). Moreover, the *in vivo* administration of milk-derived EVs led to a 50% reduction in PEDV-induced diarrhea and a decrease in mortality rates in piglets. Regarding BLV infection, previous studies have documented changes in the molecular cargo of milk-derived EVs, including variations in mRNA, miRNA, and protein profiles in infected cattle (19, 57–60). These findings support the hypothesis that milk- and colostrum-derived EVs are not only carriers of bioactive molecules but also active players in antiviral defense and immune regulation. In this context, the observed changes in the protein cargo of colostral EVs in the present study further suggest that BLV infection may influence vesicle-mediated immune signaling pathways, potentially affecting the repertoire of immunological signals transferred from mother to neonate during the early postnatal life.

The functional consequences of the observed proteomic shifts in colostral EVs from BLV-infected cows remain to be experimentally addressed. The enrichment of IDO1 in EVs from BLV-infected cows suggests that these vesicles may transfer immunosuppressive signals to neonatal immune cells, potentially suppressing T lymphocyte proliferation and promoting regulatory T cell differentiation through tryptophan catabolism along the kynurenine pathway (45). Complementarily, the reduced abundance of ANXA1 suggests additional immune dysregulation, as this protein promotes inflammation resolution and macrophage reprogramming toward a pro-resolving phenotype (44). Future experiments evaluating the effect of colostral EVs on macrophage polarization would provide further insight into the broader immunomodulatory potential of these vesicles in the neonatal context.

In conclusion, this study provides the first proteomic characterization of EVs derived from bovine colostrum in the context of BLV infection. Although the overall biophysical properties of EVs were comparable between infected and uninfected animals, BLV infection was associated with modifications in abundance patterns in vesicular protein cargo, predominantly impacting proteins involved in immune regulation and cellular signaling. Consistent with previous studies on milk-derived EVs from BLV-infected cattle (19), viral proteins were not detected in colostral EVs, suggesting that the observed proteome remodeling reflects host-derived responses to infection rather than direct viral cargo packaging. These findings emphasize the potential of colostral EVs as markers of the maternal immune status and imply that viral infection may influence the molecular information transferred from dam to calf via colostrum. Further research is necessary to understand the biological significance of these modifications and their potential effects on neonatal immunity.

## Data Availability

The data presented in the study are deposited in the Figshare repository. https://doi.org/10.6084/m9.figshare.32997560.
