# Supplementary material for: Differential protein abundance in colostrum-derived extracellular vesicles from bovine leukemia virus-infected cattle are implicated in immune regulation and inflammation
Source: Front Immunol. 2026 Jul 29;17:1856022. doi: 10.3389/fimmu.2026.1856022 (PMC13461735; doi:10.3389/fimmu.2026.1856022)
Supplement: Supplementary file 1 [file Supplementaryfile1.docx]

***Supplementary Material***

(A)

(B)

**Supplementary Figure 1**: SDS-PAGE gels of total protein extracts (30 µg) from BLV(–) (A) and BLV(+) (B) samples. Dual Color molecular weight marker was used as a reference marker.

**Supplementary Table 1:** Protein ab undance matrix. ^1^UniProtKB accession Identification

| **Phenotype** | **BLV(-)** | | | | **BLV(+)** | | | |
| --- | --- | --- | --- | --- | --- | --- | --- | --- |
| **Protein_ID^1^** | **363_VEs** | **386_VEs** | **423_VEs** | **475_VEs** | **379_VEs-I** | **283_VEs-I** | **393_VEs-I** | **332_VEs-I** |
| A0A140T894 | 2659520 | 7067260 | 10531100 | 8243820 | 12274100 | 11803700 | 11975600 | 10443300 |
| A0A140T8A5 | 22916800 | 54707300 | 81589400 | 78634400 | 65321400 | 83279600 | 163325000 | 67365600 |
| A0A140T8A9 | 6958850000 | 2684660000 | 4295990000 | 1597120000 | 3953160000 | 2375410000 | 3384470000 | 3780750000 |
| A0A3Q1LFK0 | 3176730 | 15023700 | 10820900 | 15181400 | 8951440 | 5059250 | 2425490 | 4679350 |
| A0A3Q1LGL7 | 214485 | 63529.8 | 29820.2 | 494748 | 40996.9 | 0 | 25471.2 | 114639 |
| A0A3Q1LI44 | 14250200 | 8047310 | 5254120 | 10977700 | 11036000 | 4203800 | 4441970 | 1771190 |
| A0A3Q1LI72 | 3871790 | 776737 | 1534430 | 1182180 | 1055290 | 1064020 | 1213030 | 1851830 |
| A0A3Q1LIB4 | 1042020 | 1584970 | 2653520 | 3586240 | 1721750 | 2693310 | 2463750 | 1619830 |
| A0A3Q1LIW3 | 517213 | 1113850 | 1012510 | 432539 | 666930 | 676584 | 588548 | 2651760 |
| A0A3Q1LJ64 | 273408 | 482267 | 728813 | 715972 | 571785 | 1409740 | 698192 | 508136 |
| A0A3Q1LJG7 | 752886 | 550082 | 41897.7 | 674337 | 759911 | 755756 | 306101 | 309456 |
| A0A3Q1LJT1 | 1038380 | 891144 | 824912 | 1786280 | 153147 | 53621.6 | 152163 | 329925 |
| A0A3Q1LJY8 | 188849 | 273491 | 346938 | 953934 | 228211 | 360800 | 855902 | 842641 |
| A0A3Q1LK49 | 6288560 | 716443 | 2639240 | 786705 | 879116 | 622051 | 1309300 | 1077030 |
| A0A3Q1LKM6 | 0 | 16594.1 | 1335470 | 1199450 | 0 | 495557 | 608340 | 0 |
| A0A3Q1LLH4 | 0 | 146957 | 214660 | 176157 | 114057 | 193452 | 244892 | 197306 |
| A0A3Q1LMW6 | 349102 | 118774 | 1102530 | 0 | 373108 | 1874640 | 3685040 | 837728 |
| A0A3Q1LND7 | 153517 | 277480 | 717942 | 1386430 | 1283300 | 1194120 | 1631690 | 719801 |
| A0A3Q1LNG3 | 0 | 33509.1 | 73200.1 | 169431 | 163909 | 144739 | 214231 | 51201.7 |
| A0A3Q1LPG0 | 170740000 | 90413100 | 41950000 | 18585600 | 51395500 | 59109300 | 91273200 | 15534400 |
| A0A3Q1LPI6 | 67745000 | 65318800 | 20009600 | 55354700 | 29484300 | 30894500 | 19297900 | 25831900 |
| A0A3Q1LPQ0 | 1771770 | 622962 | 1341320 | 429411 | 1078100 | 1431600 | 2854170 | 854163 |
| A0A3Q1LQ02 | 2393640 | 872925 | 3956270 | 1318350 | 1079450 | 2189390 | 2936560 | 955827 |
| A0A3Q1LQB6 | 548998 | 363000 | 558822 | 665722 | 367813 | 905978 | 1988890 | 1099010 |
| A0A3Q1LQE4 | 1424540 | 540272 | 4714150 | 624521 | 2895150 | 5680700 | 4055390 | 2984570 |
| A0A3Q1LQK2 | 260475 | 328445 | 281079 | 645535 | 129737 | 217690 | 252085 | 132324 |
| A0A3Q1LQU2 | 268202 | 239313 | 528294 | 683121 | 433476 | 547246 | 1685750 | 853522 |
| A0A3Q1LQY1 | 616025 | 1722580 | 822024 | 12951300 | 2466110 | 1986060 | 2230040 | 2835350 |
| A0A3Q1LR40 | 5098750 | 906358 | 2334560 | 1669860 | 1355720 | 1419440 | 1872150 | 1277900 |
| A0A3Q1LRM2 | 0 | 61684.2 | 77998.2 | 363285 | 62778.6 | 42905.4 | 132990 | 163951 |
| A0A3Q1LRP5 | 4964800 | 609821 | 1155830 | 344788 | 1080320 | 1033930 | 924282 | 685272 |
| A0A3Q1LRW6 | 212202 | 295923 | 2748430 | 2610750 | 449545 | 4255670 | 240219 | 416738 |
| A0A3Q1LRX3 | 0 | 518672 | 91998.3 | 2024480 | 0 | 603691 | 768562 | 554058 |
| A0A3Q1LSP7 | 125146 | 868765 | 613525 | 1607260 | 1122990 | 897147 | 1099850 | 1697650 |
| A0A3Q1LSV1 | 4819840 | 6714270 | 19519600 | 3029530 | 15097300 | 9455820 | 9000370 | 7080010 |
| A0A3Q1LT19 | 10313100 | 14734100 | 11179500 | 4321390 | 10543200 | 8393250 | 28987800 | 9468420 |
| A0A3Q1LUB1 | 0 | 23671.3 | 31038 | 0 | 47679 | 20973.2 | 369539 | 85250 |
| A0A3Q1LUE9 | 25933500 | 348292000 | 205672000 | 2412390 | 172310000 | 207589000 | 77287000 | 18406600 |
| A0A3Q1LUG9 | 2109430 | 802337 | 1738130 | 288347 | 1744940 | 1473910 | 1831270 | 1256460 |
| A0A3Q1LUP7 | 278000 | 1046340 | 1769940 | 254783 | 1402440 | 815940 | 186826 | 308579 |
| A0A3Q1LUZ7 | 142765 | 0 | 290159 | 187832 | 126119 | 92937.1 | 427030 | 63292.1 |
| A0A3Q1LVC7 | 13524000 | 28903700 | 59013400 | 81847200 | 33184800 | 38921800 | 85257500 | 48453000 |
| A0A3Q1LVV7 | 30344500 | 16804400 | 39398500 | 3099560 | 39676700 | 33826400 | 18066000 | 9212420 |
| A0A3Q1LW01 | 523476 | 856139 | 2120460 | 1560220 | 1197710 | 1813150 | 3472830 | 1594540 |
| A0A3Q1LWD3 | 1107650 | 1221450 | 243509 | 3957380 | 281868 | 158508 | 270245 | 391732 |
| A0A3Q1LWR9 | 654678 | 906634 | 1490750 | 1475020 | 1566420 | 1266900 | 1512760 | 1023160 |
| A0A3Q1LWU8 | 161984 | 258716 | 373301 | 618926 | 332889 | 390495 | 600533 | 604832 |
| A0A3Q1LX93 | 55220 | 108400 | 323281 | 178150 | 248152 | 348220 | 460456 | 349521 |
| A0A3Q1LXC8 | 397309 | 46441.1 | 211739 | 185318 | 244257 | 93094.3 | 296346 | 214343 |
| A0A3Q1LXF3 | 333041 | 1045820 | 768873 | 1466470 | 659527 | 1633390 | 5767020 | 4142290 |
| A0A3Q1LXR2 | 11357100 | 22530900 | 24189400 | 81244600 | 31338600 | 39652500 | 52697500 | 45858500 |
| A0A3Q1LY00 | 759544 | 2946300 | 5083550 | 5656410 | 2067420 | 2940330 | 8367650 | 4788650 |
| A0A3Q1LZ01 | 6847680 | 3418860 | 4648900 | 7835940 | 3249620 | 2248510 | 6611720 | 2858940 |
| A0A3Q1LZ40 | 668470 | 0 | 0 | 2265800 | 940245 | 782358 | 823946 | 107290 |
| A0A3Q1LZC9 | 447755 | 1134930 | 4660750 | 3319790 | 2736760 | 1236810 | 1697790 | 711841 |
| A0A3Q1LZZ0 | 4145700 | 875562 | 2553980 | 1035110 | 1715120 | 1098500 | 1512980 | 1475780 |
| A0A3Q1M0D7 | 2741520 | 6775050 | 4961610 | 21201700 | 9491000 | 10105200 | 9515210 | 6256710 |
| A0A3Q1M0I1 | 112405 | 194822 | 139819 | 275082 | 103014 | 192416 | 868846 | 176284 |
| A0A3Q1M130 | 240421 | 1054370 | 2395710 | 1378980 | 1419010 | 1682860 | 1406210 | 713092 |
| A0A3Q1M1A5 | 822543 | 221453 | 494522 | 124362 | 384589 | 191752 | 380245 | 537399 |
| A0A3Q1M1B6 | 201120 | 122793 | 1575610 | 151649 | 279326 | 1494900 | 2280290 | 533506 |
| A0A3Q1M2P8 | 174864 | 68875.4 | 158496 | 1327770 | 390887 | 199311 | 519510 | 347206 |
| A0A3Q1M2V6 | 2164310 | 313596 | 1356190 | 490617 | 1698030 | 542447 | 305860 | 1411230 |
| A0A3Q1M3A8 | 820207 | 1253400 | 401433 | 1390630 | 252913 | 584874 | 440321 | 324609 |
| A0A3Q1M3I6 | 26194.2 | 31655.3 | 57616.4 | 0 | 37005.2 | 55600 | 118468 | 33986.1 |
| A0A3Q1M3K7 | 9142140 | 18874600 | 25189500 | 40835000 | 22007200 | 28513900 | 38752200 | 30190900 |
| A0A3Q1M3Z5 | 6753870 | 1791100 | 3500610 | 2265070 | 2609610 | 1274280 | 3761440 | 2983330 |
| A0A3Q1M458 | 23492300 | 5198390 | 7862540 | 9063750 | 13487600 | 23419300 | 7723990 | 3741910 |
| A0A3Q1M481 | 100291 | 197223 | 345566 | 215712 | 275354 | 309083 | 549206 | 117260 |
| A0A3Q1M4F5 | 3028730 | 5231720 | 16035100 | 18002500 | 11156700 | 12859400 | 10890000 | 8966140 |
| A0A3Q1M4H2 | 251878 | 425111 | 871935 | 1833890 | 866337 | 925418 | 989018 | 1189490 |
| A0A3Q1M5A2 | 1174850 | 2268160 | 9266980 | 5173550 | 5490160 | 7687840 | 5051450 | 3237270 |
| A0A3Q1M5R4 | 10621500 | 16900200 | 36843400 | 16609700 | 30883600 | 26473100 | 20093000 | 13814400 |
| A0A3Q1M5V0 | 293215 | 621089 | 1160130 | 1443400 | 964684 | 1655230 | 2629810 | 1085000 |
| A0A3Q1M6J2 | 67381.9 | 92514 | 341616 | 670484 | 195589 | 601923 | 224757 | 202753 |
| A0A3Q1M6K1 | 2132790 | 4562600 | 5522340 | 17888700 | 6465550 | 4236950 | 6828370 | 8133210 |
| A0A3Q1M762 | 55931000 | 73216600 | 88919700 | 186512000 | 86628800 | 116515000 | 104986000 | 124616000 |
| A0A3Q1M7Q5 | 4795550 | 4698430 | 7324140 | 3043580 | 968267 | 3533480 | 5699070 | 6448410 |
| A0A3Q1M8J9 | 5547540 | 11752500 | 19978100 | 11916000 | 15021700 | 20302000 | 28224100 | 16627800 |
| A0A3Q1M8N6 | 1855730 | 307287 | 1072830 | 516602 | 984018 | 493002 | 799580 | 581056 |
| A0A3Q1M903 | 67391.5 | 0 | 1014110 | 425613 | 214390 | 558303 | 1146580 | 138826 |
| A0A3Q1M909 | 25618.4 | 143956 | 154960 | 255843 | 142946 | 255584 | 515623 | 207042 |
| A0A3Q1M913 | 109634 | 114388 | 205949 | 293682 | 213027 | 240530 | 505572 | 365892 |
| A0A3Q1M9W0 | 720596 | 1959530 | 2742820 | 1472170 | 3061730 | 2859410 | 3817450 | 1633190 |
| A0A3Q1MA07 | 365002 | 959350 | 1152530 | 1132930 | 603695 | 1041100 | 2369630 | 977020 |
| A0A3Q1MA33 | 526270 | 722166 | 1237490 | 311083 | 999881 | 1288940 | 241668 | 399198 |
| A0A3Q1MAQ8 | 3116270 | 5125140 | 8559790 | 11751600 | 9082810 | 11309900 | 6153890 | 8997180 |
| A0A3Q1MAZ8 | 0 | 139456 | 175832 | 470864 | 498767 | 523712 | 412697 | 135229 |
| A0A3Q1MB37 | 1007440 | 1111910 | 1050880 | 2648130 | 1077440 | 1996530 | 2159640 | 1214710 |
| A0A3Q1MBS7 | 610073 | 1044610 | 2318540 | 3376600 | 1641610 | 2216940 | 2263030 | 2077530 |
| A0A3Q1MC95 | 1378290 | 2290680 | 2163900 | 1689010 | 2723750 | 1032290 | 2646270 | 1246340 |
| A0A3Q1MD47 | 608452 | 17957.3 | 46325.6 | 66600.2 | 225349 | 129158 | 199756 | 139363 |
| A0A3Q1MDA4 | 55543000 | 2095830 | 5482640 | 1267260 | 19563400 | 10952200 | 4167840 | 1769210 |
| A0A3Q1MDC6 | 1738040 | 3679800 | 5055530 | 8115440 | 4288450 | 6590250 | 5657570 | 4562560 |
| A0A3Q1MDG3 | 0 | 47061.9 | 101660 | 0 | 27632.7 | 57466.9 | 284083 | 88911.1 |
| A0A3Q1MDT7 | 1808990 | 1205510 | 4026620 | 1212160 | 1789470 | 844965 | 4068350 | 4833600 |
| A0A3Q1ME19 | 1384610 | 2835180 | 2951830 | 7323230 | 5691880 | 5608250 | 4215890 | 4581860 |
| A0A3Q1MEI7 | 517572 | 745299 | 1126750 | 1645660 | 864064 | 1290140 | 1320110 | 941940 |
| A0A3Q1MEL2 | 230109 | 434104 | 534531 | 434559 | 414310 | 611297 | 804305 | 523657 |
| A0A3Q1MEM9 | 4040840 | 25327600 | 37609400 | 82234100 | 30244700 | 15856500 | 47526200 | 76335600 |
| A0A3Q1MFR4 | 0 | 180006 | 60631.8 | 239819 | 179557 | 157827 | 123657 | 117954 |
| A0A3Q1MFV1 | 75919600 | 213608000 | 184732000 | 255560000 | 196224000 | 185948000 | 159235000 | 339590000 |
| A0A3Q1MG73 | 612664 | 355899 | 293477 | 56341.6 | 193561 | 54387.5 | 201434 | 0 |
| A0A3Q1MG97 | 0 | 92792.6 | 69077.3 | 193802 | 252078 | 200214 | 161968 | 73992.1 |
| A0A3Q1MGB8 | 1442910 | 157440 | 585533 | 123999 | 325705 | 255517 | 237783 | 475914 |
| A0A3Q1MGP0 | 34950.1 | 73926.1 | 71249.5 | 125680 | 60880.8 | 164496 | 286814 | 255884 |
| A0A3Q1MGP1 | 4156450 | 3774370 | 6784170 | 3856890 | 420952 | 2939670 | 6850080 | 6542820 |
| A0A3Q1MHX8 | 4577790 | 7740860 | 10894500 | 17440100 | 8662170 | 11720300 | 19589200 | 13966500 |
| A0A3Q1MHZ1 | 157724 | 117461 | 456230 | 167234 | 243065 | 309710 | 366797 | 438613 |
| A0A3Q1MI34 | 36036.9 | 91955.5 | 235285 | 365674 | 185408 | 154722 | 467820 | 355820 |
| A0A3Q1MIB0 | 61519.5 | 85103 | 175602 | 144934 | 46598.4 | 376821 | 823274 | 312449 |
| A0A3Q1MIL5 | 5167370 | 7576430 | 4649940 | 4376980 | 3119160 | 3153910 | 2043260 | 2281900 |
| A0A3Q1MJ73 | 141975000 | 37622400 | 57192300 | 141637000 | 78366200 | 130025000 | 58113500 | 50085200 |
| A0A3Q1MJK1 | 214098 | 269614 | 437560 | 720888 | 518854 | 637383 | 882188 | 519322 |
| A0A3Q1MJT2 | 25405100 | 296697 | 512770 | 139055 | 300661 | 282142 | 374631 | 42491.6 |
| A0A3Q1MJU0 | 1232320 | 2223770 | 3339580 | 2803410 | 2634560 | 3225120 | 4364620 | 3782690 |
| A0A3Q1MK61 | 3972770 | 8337470 | 6215710 | 20418600 | 19819500 | 15148900 | 11717500 | 6557130 |
| A0A3Q1MKI2 | 4605370 | 33382100 | 5584200 | 1720080 | 15196300 | 16310800 | 1440610 | 1032490 |
| A0A3Q1ML89 | 518519 | 2714470 | 2676510 | 5750430 | 1184730 | 5019580 | 2555980 | 3532550 |
| A0A3Q1MM35 | 604171 | 251347 | 304130 | 0 | 334006 | 401382 | 339914 | 272845 |
| A0A3Q1MM55 | 147597 | 617621 | 1852980 | 946936 | 992431 | 1119720 | 2213040 | 893627 |
| A0A3Q1MMI3 | 0 | 33116 | 176382 | 984427 | 50976.8 | 41944.2 | 511169 | 158994 |
| A0A3Q1MMV0 | 47385.6 | 173651 | 214228 | 236414 | 307658 | 337221 | 322091 | 64746.2 |
| A0A3Q1MNN2 | 230038 | 293577 | 371295 | 359263 | 451496 | 560254 | 879794 | 490584 |
| A0A3Q1MNQ1 | 93281.8 | 145516 | 155942 | 130679 | 105510 | 233395 | 383181 | 145659 |
| A0A3Q1MPD6 | 221226 | 637634 | 1050180 | 406242 | 906939 | 1727610 | 1188000 | 491786 |
| A0A3Q1MPS2 | 170870 | 577918 | 618749 | 1321410 | 525319 | 697603 | 1151760 | 1304400 |
| A0A3Q1MPS4 | 113602 | 58730.1 | 385069 | 0 | 32331.2 | 395744 | 212900 | 123656 |
| A0A3Q1MQM0 | 594539 | 129479 | 349700 | 160009 | 194249 | 161922 | 476740 | 337093 |
| A0A3Q1MQM3 | 3932930 | 7173330 | 7469950 | 16933800 | 13444300 | 13923300 | 13760600 | 11655100 |
| A0A3Q1MR39 | 993332 | 1572450 | 2293000 | 4078050 | 1870690 | 2818100 | 2705790 | 2197780 |
| A0A3Q1MR41 | 1120920 | 88737 | 594737 | 260715 | 240350 | 264646 | 835133 | 265905 |
| A0A3Q1MRJ9 | 1607750 | 764851 | 1282360 | 1442530 | 1032860 | 1251630 | 1347080 | 1113130 |
| A0A3Q1MRQ2 | 1589230 | 221530 | 648222 | 117867 | 131653 | 249776 | 61562 | 858640 |
| A0A3Q1MRU9 | 131653 | 303363 | 328313 | 1013790 | 319533 | 397923 | 911231 | 652680 |
| A0A3Q1MS33 | 1028420 | 3384220 | 7851200 | 6345410 | 4198580 | 5895280 | 12835800 | 2973930 |
| A0A3Q1MT05 | 3171890 | 3661200 | 6755850 | 4644180 | 3208460 | 4690630 | 12428700 | 4981540 |
| A0A3Q1MT60 | 982573 | 1718130 | 3135410 | 2188140 | 2156880 | 1722970 | 2686490 | 2674710 |
| A0A3Q1MTH2 | 46780 | 130470 | 233571 | 259430 | 159978 | 245194 | 349290 | 179835 |
| A0A3Q1MU35 | 0 | 99297.1 | 107474 | 155510 | 51965.8 | 115665 | 246181 | 156081 |
| A0A3Q1MUJ8 | 735964 | 514815 | 797867 | 1759680 | 700396 | 1050490 | 1032970 | 707674 |
| A0A3Q1MUN0 | 279280 | 182611 | 489301 | 246476 | 893977 | 1040030 | 515503 | 505746 |
| A0A3Q1MUN1 | 235112 | 750150 | 951580 | 2234240 | 781210 | 1300700 | 1968530 | 879958 |
| A0A3Q1MUU9 | 29696.3 | 89061.2 | 177830 | 161773 | 144973 | 51870.9 | 290216 | 61588.2 |
| A0A3Q1MUV9 | 17619800 | 2370020 | 6488090 | 2357180 | 7001880 | 3045650 | 5981410 | 2863170 |
| A0A3Q1MZW0 | 3803240 | 5934320 | 10850000 | 13110400 | 6029580 | 6404250 | 4395720 | 2879210 |
| A0A3Q1N2F2 | 0 | 52669.5 | 253956 | 564912 | 0 | 180188 | 38005.1 | 419671 |
| A0A3Q1N2S6 | 128198 | 359283 | 603318 | 399621 | 301399 | 503342 | 374122 | 399570 |
| A0A3Q1N4F3 | 51048.8 | 295063 | 359616 | 273615 | 226937 | 377897 | 381489 | 168939 |
| A0A3Q1N4Y8 | 1751530 | 335175 | 1296330 | 247020 | 1626720 | 662307 | 849843 | 860209 |
| A0A3Q1N552 | 45181.7 | 108447 | 194251 | 256607 | 155151 | 224222 | 386819 | 22569.7 |
| A0A3Q1N570 | 4441290 | 11359100 | 12099700 | 40584700 | 10299500 | 10789100 | 13386800 | 16257100 |
| A0A3Q1N5X6 | 6310080 | 1274980 | 3895330 | 1990790 | 2873540 | 2073660 | 4472150 | 2791210 |
| A0A3Q1N6S6 | 48722.5 | 442450 | 629674 | 717181 | 368210 | 501727 | 774317 | 432481 |
| A0A3Q1N802 | 890131 | 1295760 | 1961920 | 1673670 | 1683410 | 1451860 | 3020420 | 1907930 |
| A0A3Q1N9V7 | 186138 | 485437 | 600014 | 693985 | 422614 | 1225190 | 628106 | 347831 |
| A0A3Q1NA28 | 223010 | 342532 | 675189 | 1091990 | 371261 | 429036 | 587824 | 503923 |
| A0A3Q1NA59 | 9593430 | 14549800 | 23919300 | 24825400 | 18146600 | 22796100 | 24573200 | 22894100 |
| A0A3Q1NBQ9 | 1965590 | 8169960 | 5984020 | 36787400 | 7938810 | 10403300 | 5700000 | 13648800 |
| A0A3Q1NEI6 | 0 | 72794.6 | 163493 | 158300 | 89156.7 | 126820 | 254926 | 143145 |
| A0A3Q1NHX4 | 228798 | 0 | 119505 | 38828.3 | 104657 | 224830 | 620185 | 354583 |
| A0A3Q1NIZ0 | 47753.1 | 26937.5 | 77046.9 | 873223 | 31369.2 | 22391.2 | 277658 | 103584 |
| A0A3Q1NJ45 | 291054 | 463970 | 503972 | 1175690 | 649195 | 622489 | 783832 | 940065 |
| A0A3Q1NLW9 | 5407530 | 10642400 | 10408200 | 49758800 | 13131600 | 13628900 | 11457500 | 19000900 |
| A0A3Q1NM87 | 0 | 38678.3 | 69015.1 | 0 | 0 | 71476.8 | 228680 | 41045.6 |
| A0A3Q1NMD6 | 63853.7 | 122949 | 241968 | 658474 | 152771 | 60859.3 | 467373 | 317236 |
| A0A3S5ZPA0 | 55296.5 | 0 | 30021.5 | 0 | 15389.5 | 0 | 54301.9 | 34301.2 |
| A0A3S5ZPB0 | 5139560 | 15569800 | 33097100 | 20483200 | 18187800 | 26359400 | 29457500 | 17262500 |
| A0A3S5ZPC1 | 269842 | 392756 | 776938 | 1101350 | 719028 | 760060 | 876691 | 717656 |
| A0A3S5ZPG9 | 1325890 | 4758100 | 4453140 | 45813900 | 3348360 | 5588380 | 63281400 | 54253200 |
| A0A3S5ZPJ7 | 750652 | 2817050 | 3418230 | 2494010 | 3697390 | 3893000 | 5597880 | 3525720 |
| A0A3S5ZPM3 | 6359650 | 6692310 | 6941010 | 5300480 | 5228730 | 6050830 | 10745900 | 5634460 |
| A0A452DHW7 | 735801000 | 155898000 | 276254000 | 42011300 | 158336000 | 59714300 | 95764300 | 89521800 |
| A0A452DHZ3 | 7137030 | 561146 | 1404470 | 608913 | 1792210 | 720607 | 1772340 | 808775 |
| A0A452DHZ5 | 1650160 | 746515 | 1256840 | 350953 | 515526 | 688675 | 1364380 | 627760 |
| A0A452DI02 | 115865 | 304212 | 519551 | 609078 | 338458 | 452852 | 705899 | 458955 |
| A0A452DI45 | 1404390 | 2994860 | 3630590 | 5119420 | 3785840 | 5491450 | 6382140 | 3785410 |
| A0A452DI66 | 17699800 | 3336150 | 7383130 | 4086800 | 10466500 | 6295580 | 5529840 | 3809420 |
| A0A452DID6 | 959187 | 2147420 | 1477610 | 1340490 | 1205470 | 1443110 | 1571760 | 2082670 |
| A0A452DIU9 | 0 | 52668.1 | 144530 | 469733 | 104060 | 202499 | 618114 | 117325 |
| A0A452DIX8 | 1295680 | 960910 | 1831270 | 1420810 | 907958 | 1896980 | 2666000 | 1438350 |
| A0A452DJ03 | 13249300 | 1539000 | 5424210 | 2128750 | 3317020 | 2003460 | 4813110 | 2840740 |
| A0A452DJ12 | 397177 | 1764110 | 1564410 | 2469260 | 1400470 | 5734960 | 1131530 | 407664 |
| A0A452DJ96 | 1344710 | 2209470 | 3212790 | 3739040 | 2575020 | 4063240 | 6561350 | 3562680 |
| A0A452DJA9 | 26394400 | 2302200 | 5097320 | 1283440 | 7478290 | 3303290 | 3359260 | 3045930 |
| A0A452DJD0 | 6462720 | 3264770 | 3513440 | 2090210 | 1733150 | 1652820 | 1580340 | 2968730 |
| A0A452DJE7 | 201910 | 474899 | 622021 | 775549 | 335765 | 617593 | 1197260 | 628056 |
| A0A452DJG2 | 2430240 | 3669380 | 4389930 | 6188430 | 3840330 | 6037840 | 7328860 | 6466060 |
| A0A452DK44 | 115327 | 174370 | 113236 | 0 | 84434.9 | 75535.9 | 208210 | 61391.6 |
| A0A452DKH0 | 321993 | 837113 | 1633760 | 1402670 | 1249510 | 1607730 | 2795950 | 1241310 |
| A0AAA9RRE8 | 296157 | 531554 | 1187280 | 597759 | 772504 | 1148190 | 1030360 | 622715 |
| A0AAA9RRF4 | 144851 | 262548 | 310060 | 540530 | 153208 | 262651 | 202545 | 231055 |
| A0AAA9RSR9 | 3474840 | 5321220 | 2872360 | 15412600 | 2924940 | 1096000 | 1189280 | 4138710 |
| A0AAA9RTB6 | 23393900 | 44670100 | 71976600 | 57689200 | 64143400 | 80706100 | 85244700 | 49909800 |
| A0AAA9RTG5 | 799015 | 123430 | 744779 | 51158 | 489575 | 266060 | 139608 | 152140 |
| A0AAA9RTT5 | 14472400 | 3220710 | 8056140 | 3715780 | 4645090 | 3787640 | 5723400 | 5214630 |
| A0AAA9RU21 | 9051980 | 16090200 | 29728700 | 40841200 | 14880800 | 18824800 | 22136300 | 32524100 |
| A0AAA9RU48 | 0 | 0 | 662566 | 218369 | 407176 | 490330 | 276662 | 1159350 |
| A0AAA9RU90 | 1704390 | 1511150 | 4318270 | 335402 | 968746 | 2851840 | 6084680 | 1166740 |
| A0AAA9RUL8 | 2577290000 | 4835850000 | 5353730000 | 5393720000 | 6055490000 | 6425960000 | 3628270000 | 4967960000 |
| A0AAA9RUQ7 | 541250 | 1482240 | 2085300 | 1788130 | 1188390 | 1753890 | 3939780 | 1922300 |
| A0AAA9RVD6 | 846109 | 1522370 | 725494 | 2844560 | 1017530 | 1286130 | 1959250 | 2076580 |
| A0AAA9RVE1 | 54369.4 | 186332 | 295258 | 328931 | 208218 | 264930 | 275769 | 173035 |
| A0AAA9RVP4 | 124625 | 385894 | 1462050 | 2092140 | 1322710 | 506425 | 615624 | 279817 |
| A0AAA9RW27 | 147406 | 450092 | 828146 | 724357 | 476544 | 1030600 | 1696700 | 1165600 |
| A0AAA9RWM7 | 150155 | 243955 | 462068 | 274207 | 290457 | 307157 | 1182210 | 482675 |
| A0AAA9RWM9 | 315808 | 495777 | 309319 | 131645 | 200527 | 580228 | 487400 | 646593 |
| A0AAA9RWN7 | 180278 | 507131 | 704808 | 583536 | 428089 | 472786 | 999386 | 1651180 |
| A0AAA9RWP8 | 778091 | 453364 | 1151670 | 1755720 | 479828 | 1078970 | 982162 | 1428760 |
| A0AAA9RWU4 | 101730 | 140526 | 137458 | 106877 | 119375 | 235620 | 427637 | 41490.4 |
| A0AAA9RY95 | 1817260 | 382679 | 1283170 | 720014 | 606406 | 496960 | 1023770 | 617547 |
| A0AAA9RYS2 | 709135 | 694109 | 1991060 | 1202910 | 1603070 | 2783400 | 1694230 | 1551660 |
| A0AAA9RYW7 | 65909.2 | 536758 | 423379 | 2384090 | 1041230 | 994788 | 1775170 | 600758 |
| A0AAA9RZ54 | 377136 | 721748 | 1013970 | 2116790 | 1037850 | 772488 | 1532000 | 1243040 |
| A0AAA9RZ65 | 493261 | 1150630 | 1769660 | 5257000 | 1180520 | 3007700 | 2199410 | 14203400 |
| A0AAA9RZL3 | 369433 | 1106720 | 694153 | 3576790 | 1193950 | 1286630 | 2416860 | 1577500 |
| A0AAA9RZL9 | 586837 | 1597870 | 1486400 | 3047000 | 1618870 | 2449850 | 3190610 | 1059910 |
| A0AAA9S0J0 | 273365 | 501904 | 829701 | 544293 | 1309400 | 700374 | 3624140 | 2912660 |
| A0AAA9S0T2 | 13719000 | 3438610 | 8994510 | 3502550 | 8031250 | 5920450 | 4936940 | 2853530 |
| A0AAA9S490 | 612611000 | 244454000 | 595032000 | 220197000 | 267721000 | 229226000 | 431172000 | 307130000 |
| A0AAA9S4E1 | 0 | 32764.1 | 99078.9 | 161686 | 65967.3 | 106135 | 280737 | 0 |
| A0AAA9S7F8 | 213013 | 258271 | 377209 | 878210 | 801995 | 535488 | 1245690 | 917703 |
| A0AAA9S7I0 | 0 | 89274.1 | 189386 | 180567 | 63861.5 | 61757.1 | 227577 | 98341.3 |
| A0AAA9S835 | 24326300 | 1927600 | 9081100 | 4734750 | 8782500 | 2651740 | 16070600 | 14743100 |
| A0AAA9S8E2 | 912454 | 569341 | 379873 | 292158 | 353599 | 580324 | 805442 | 1059400 |
| A0AAA9S8V6 | 1093120 | 3028660 | 3275370 | 3746420 | 4033530 | 3863650 | 2110210 | 2147380 |
| A0AAA9S9Q3 | 0 | 0 | 291592 | 0 | 214107 | 356400 | 279955 | 325292 |
| A0AAA9SA40 | 123632 | 95101.8 | 212789 | 455235 | 245577 | 310529 | 309278 | 288893 |
| A0AAA9SAG2 | 29781.9 | 0 | 66239.1 | 431228 | 124695 | 34844.4 | 194367 | 216444 |
| A0AAA9SBL4 | 1458070 | 682629 | 218394 | 0 | 1208160 | 508339 | 793345 | 176955 |
| A0AAA9SBV4 | 427293 | 996872 | 1221480 | 2159480 | 1070010 | 1608810 | 2396690 | 2013210 |
| A0AAA9SCA4 | 407604 | 0 | 193596 | 135404 | 110971 | 52877.5 | 106810 | 122577 |
| A0AAA9SD79 | 114302 | 919280 | 545780 | 6600410 | 271904 | 665171 | 2457290 | 1903480 |
| A0AAA9SDB6 | 10278800 | 7976610 | 15551900 | 23965400 | 10325300 | 12633400 | 14931000 | 13139500 |
| A0AAA9SDR6 | 2514630 | 3737520 | 4793520 | 8450190 | 5098200 | 5961740 | 5763150 | 6725020 |
| A0AAA9SE43 | 70867.9 | 157344 | 36875.6 | 9432230 | 64364.3 | 16242.3 | 101054 | 532965 |
| A0AAA9SEK8 | 90542.2 | 253871 | 74343.8 | 3826010 | 63391.9 | 52953.1 | 312526 | 301985 |
| A0AAA9SEV1 | 378564 | 842450 | 996450 | 2073220 | 1743110 | 1680680 | 1727990 | 1095000 |
| A0AAA9SF14 | 58994.7 | 371044 | 368123 | 149868 | 155301 | 373522 | 907119 | 153468 |
| A0AAA9SF39 | 1581540000 | 1671860000 | 1814770000 | 989135000 | 1307750000 | 1722650000 | 1459240000 | 1725020000 |
| A0AAA9SG26 | 2377270 | 8319310 | 9895880 | 12335600 | 10108300 | 12142600 | 19926700 | 17184800 |
| A0AAA9SG32 | 18064.4 | 45057.8 | 90502.2 | 25582.6 | 76609.7 | 111048 | 298313 | 73801.6 |
| A0AAA9SGV1 | 676756 | 178160 | 263626 | 385950 | 0 | 0 | 0 | 91454.5 |
| A0AAA9SGY6 | 1756740 | 659126 | 1864430 | 69154.6 | 1364350 | 287962 | 910202 | 500563 |
| A0AAA9SH17 | 7134690 | 1321660 | 4225650 | 1599290 | 2945960 | 2010780 | 3351140 | 2955680 |
| A0AAA9SHI3 | 523284 | 834698 | 696794 | 793240 | 34160.4 | 621645 | 303464 | 466607 |
| A0AAA9SIE7 | 404597 | 379142 | 1145870 | 272443 | 483129 | 1180780 | 1897650 | 457179 |
| A0AAA9SIZ7 | 405627 | 796393 | 665489 | 3544460 | 1477160 | 955027 | 1885070 | 1033290 |
| A0AAA9SJC5 | 44130.1 | 56157.5 | 171260 | 119078 | 244235 | 226527 | 298750 | 119178 |
| A0AAA9SK87 | 385410 | 217792 | 389843 | 458533 | 262145 | 263496 | 402860 | 118892 |
| A0AAA9SKJ1 | 131051000 | 20526300 | 24873000 | 10311800 | 22606100 | 15987500 | 19644400 | 15288000 |
| A0AAA9SKJ4 | 1072840 | 258826 | 900046 | 533907 | 658448 | 319845 | 554809 | 323330 |
| A0AAA9SKR8 | 5541280 | 1689000 | 2326400 | 6606800 | 6496850 | 4758870 | 2828650 | 1679590 |
| A0AAA9SL89 | 260980 | 331038 | 375244 | 1306070 | 544610 | 439957 | 1010330 | 958811 |
| A0AAA9SLZ6 | 463660 | 162287 | 701727 | 2306130 | 408298 | 694980 | 708046 | 478882 |
| A0AAA9SM26 | 3636000 | 695225 | 1725150 | 854003 | 1727670 | 938659 | 1269770 | 1265160 |
| A0AAA9SME9 | 489102 | 31850.2 | 339491 | 177017 | 77508.1 | 48695.5 | 305906 | 208767 |
| A0AAA9SMR2 | 4985210 | 6096720 | 10881100 | 13000100 | 8910760 | 9498580 | 15932200 | 9174610 |
| A0AAA9SNC1 | 8114730 | 17030700 | 12990600 | 91003900 | 20666800 | 20908600 | 17477500 | 23427200 |
| A0AAA9SNN4 | 3097780 | 3439970 | 5834870 | 4148240 | 3633390 | 4969400 | 7384490 | 5378010 |
| A0AAA9SNQ5 | 63004.4 | 201580 | 318456 | 256340 | 161011 | 414738 | 544295 | 428341 |
| A0AAA9SNV2 | 35601.2 | 170763 | 255625 | 356470 | 242397 | 321403 | 639683 | 142049 |
| A0AAA9SNY2 | 509469 | 1130060 | 1173050 | 2019590 | 1278680 | 1798000 | 2843140 | 2160960 |
| A0AAA9SNZ0 | 66856.7 | 445902 | 443380 | 2783670 | 517055 | 660142 | 598992 | 839443 |
| A0AAA9SP21 | 129451000 | 211278000 | 201055000 | 921186000 | 218734000 | 234509000 | 184887000 | 478223000 |
| A0AAA9SPQ2 | 152729 | 356785 | 363792 | 934706 | 562845 | 412202 | 317687 | 584871 |
| A0AAA9SPT2 | 2348280 | 703256 | 2090170 | 1200430 | 3257310 | 1364940 | 4022790 | 1701930 |
| A0AAA9SQ31 | 331917 | 801874 | 1436870 | 1384760 | 939154 | 1530700 | 2078280 | 1336990 |
| A0AAA9SQE0 | 61338.3 | 132256 | 215233 | 254306 | 142868 | 239072 | 744821 | 250234 |
| A0AAA9SQI1 | 121222 | 492724 | 557334 | 659155 | 320501 | 472079 | 1381270 | 509902 |
| A0AAA9SQM8 | 2182380 | 4749000 | 7361290 | 7314470 | 4601970 | 7382530 | 12657800 | 7166040 |
| A0AAA9SRD3 | 31081.2 | 246034 | 906011 | 618246 | 411060 | 1000340 | 985032 | 476159 |
| A0AAA9SRF6 | 123413 | 112233 | 77946.7 | 254157 | 37302.2 | 53978 | 115114 | 144375 |
| A0AAA9SRZ8 | 1436010 | 271965 | 775498 | 612958 | 601228 | 270385 | 535124 | 409433 |
| A0AAA9SS47 | 328621 | 86354.9 | 197244 | 130391 | 162106 | 137750 | 209379 | 197569 |
| A0AAA9SSI8 | 403841 | 819430 | 692965 | 2221690 | 501788 | 916659 | 2039440 | 1923540 |
| A0AAA9SSQ7 | 35098 | 119752 | 213996 | 242888 | 173145 | 376368 | 457520 | 321683 |
| A0AAA9SST4 | 1113480 | 1669140 | 982307 | 647277 | 337030 | 559010 | 1804070 | 466510 |
| A0AAA9SSZ3 | 283662 | 59876 | 112665 | 91680.3 | 125545 | 76418.2 | 227061 | 149279 |
| A0AAA9ST74 | 30029000 | 42133200 | 47011600 | 224550000 | 47031200 | 49165100 | 40382700 | 97983700 |
| A0AAA9STL0 | 530491 | 58512.5 | 276729 | 85720.4 | 183620 | 108910 | 423176 | 202954 |
| A0AAA9STV6 | 64945.6 | 89101.3 | 282523 | 194187 | 201166 | 553643 | 352248 | 446260 |
| A0AAA9SUB4 | 253863 | 622113 | 777913 | 805044 | 647617 | 1580340 | 981227 | 389500 |
| A0AAA9SUD5 | 479329000 | 147313000 | 97656900 | 189914000 | 50042700 | 205716000 | 1116780000 | 1011570000 |
| A0AAA9SUS5 | 6070070 | 17610700 | 24182900 | 17930800 | 20682600 | 12291500 | 14577400 | 15516100 |
| A0AAA9SVD1 | 2190740 | 532239 | 2141980 | 614400 | 2261460 | 1706060 | 3800320 | 1238430 |
| A0AAA9SVV9 | 0 | 161250 | 317467 | 235957 | 160431 | 300350 | 487723 | 216130 |
| A0AAA9SWE4 | 6479960 | 9230620 | 31464500 | 20107400 | 13758600 | 19974700 | 21493200 | 14756700 |
| A0AAA9SWX5 | 524856 | 945315 | 2206080 | 62300.9 | 1720290 | 2357720 | 1423480 | 309228 |
| A0AAA9SX90 | 26122 | 164905 | 0 | 771098 | 127994 | 29306 | 49002.2 | 309177 |
| A0AAA9SXD6 | 3009520 | 429470 | 816983 | 669349 | 782919 | 594144 | 1045560 | 792209 |
| A0AAA9SXF2 | 5959660 | 1318000 | 3062330 | 3581760 | 2275780 | 1491780 | 3030780 | 2276380 |
| A0AAA9SY97 | 599037 | 1241980 | 1352650 | 3174130 | 1260550 | 1203320 | 1494790 | 1719640 |
| A0AAA9SYC7 | 81518.3 | 320463 | 802127 | 565264 | 251971 | 443062 | 846575 | 387304 |
| A0AAA9SYV6 | 2346000 | 710466 | 1330340 | 842520 | 1945290 | 1384910 | 3751900 | 1114830 |
| A0AAA9SYX9 | 769260 | 1488290 | 1761670 | 2170390 | 3364890 | 1366760 | 1731910 | 1506720 |
| A0AAA9T1E6 | 171770000 | 160311000 | 110904000 | 193931000 | 83196500 | 158010000 | 118149000 | 118008000 |
| A0AAA9T1Q1 | 39797.6 | 618215 | 1068220 | 279039 | 668912 | 881378 | 924202 | 688046 |
| A0AAA9T211 | 115553 | 460821 | 421091 | 1129240 | 462892 | 1084970 | 944793 | 768692 |
| A0AAA9T2J7 | 476168 | 1351300 | 1449190 | 2038820 | 1415350 | 1887770 | 2733110 | 1623600 |
| A0AAA9T2W5 | 904657 | 1373310 | 1695530 | 3188090 | 3384990 | 3400590 | 1974370 | 2956310 |
| A0AAA9T2W8 | 198368 | 44790.6 | 1499320 | 55351.7 | 681809 | 1370600 | 1589980 | 379745 |
| A0AAA9T2Y5 | 1591990 | 1079580 | 1891560 | 251717 | 1337180 | 1537070 | 1771370 | 3107000 |
| A0AAA9T3I7 | 2432660 | 275814 | 1200250 | 349667 | 332381 | 338525 | 1491580 | 827257 |
| A0AAA9T4B1 | 202174 | 357499 | 373327 | 544078 | 304858 | 534668 | 1022150 | 500849 |
| A0AAA9T4C8 | 4903370 | 1040720 | 1818040 | 1068980 | 1674310 | 1172680 | 2189300 | 1768200 |
| A0AAA9T4C9 | 82444.2 | 209306 | 475174 | 417622 | 313679 | 497916 | 382809 | 362482 |
| A0AAA9T4F5 | 1969100 | 8403530 | 12231300 | 25511300 | 7801830 | 7292570 | 4612060 | 8476390 |
| A0AAA9T4S7 | 35462.5 | 312286 | 318638 | 302314 | 235650 | 189136 | 600746 | 325660 |
| A0AAA9T4S8 | 725780 | 1580860 | 3104330 | 10346600 | 1458050 | 3587770 | 2027360 | 3111680 |
| A0AAA9T5S8 | 305648 | 1146630 | 3084300 | 3591830 | 2076460 | 5805340 | 1371180 | 1748560 |
| A0AAA9T5W3 | 180488 | 784823 | 638962 | 1062880 | 1138290 | 3801000 | 1018050 | 1127100 |
| A0AAA9T5Z8 | 3084800 | 3455890 | 5788300 | 5669530 | 4420450 | 5434190 | 10155700 | 6166360 |
| A0AAA9T600 | 74461.6 | 83389 | 609209 | 951214 | 492899 | 21975.7 | 582809 | 219537 |
| A0AAA9T640 | 0 | 176483 | 437150 | 365905 | 102909 | 437652 | 603781 | 175305 |
| A0AAA9T6F1 | 0 | 120752 | 196374 | 258391 | 229437 | 421889 | 593077 | 229971 |
| A0AAA9T6M9 | 13082400 | 2701490 | 6189540 | 1540950 | 3897510 | 2354550 | 5312020 | 3721590 |
| A0AAA9T6U4 | 1347600 | 274775 | 656381 | 352654 | 471702 | 409466 | 1079930 | 296568 |
| A0AAA9T9G7 | 70627.3 | 182408 | 298711 | 303400 | 177487 | 345777 | 498179 | 318708 |
| A0AAA9T9N5 | 264239 | 189472 | 171033 | 406099 | 0 | 19069.1 | 3908410 | 2216210 |
| A0AAA9T9T5 | 404485 | 1865810 | 1205840 | 1105130 | 1298920 | 2299980 | 1254460 | 2134820 |
| A0AAA9TAD8 | 104070 | 406254 | 641515 | 550254 | 545712 | 707184 | 1675000 | 942594 |
| A0AAA9TAG5 | 5653250 | 12858600 | 22432900 | 14716200 | 15754400 | 18171100 | 20554000 | 12719700 |
| A0AAA9TBM6 | 1379550 | 55574.7 | 944801 | 278460 | 498508 | 441245 | 648716 | 550691 |
| A0AAA9TBW3 | 3812060 | 777653 | 3004570 | 891842 | 1247940 | 1353020 | 2172910 | 992498 |
| A0AAA9TCB8 | 162140 | 180441 | 279867 | 664268 | 212887 | 353508 | 1261310 | 55980.4 |
| A0AAA9TCP0 | 92236900 | 422273000 | 159142000 | 313999000 | 135780000 | 491453000 | 116089000 | 276212000 |
| A0AAA9TCS0 | 5199110 | 13007900 | 23899700 | 11635300 | 15340800 | 26177800 | 29151300 | 16369900 |
| A0AAA9TDC9 | 1376930 | 420259 | 1074110 | 158852 | 770121 | 932561 | 309222 | 541102 |
| A0AAA9TDK9 | 1754670 | 351978 | 181917 | 92474.9 | 262402 | 0 | 176137 | 71334 |
| A0AAA9TDL4 | 460611 | 202427 | 378483 | 105757 | 266020 | 201270 | 389395 | 474570 |
| A0AAA9TDP3 | 1211560 | 1392430 | 1578430 | 3179650 | 1816540 | 2697620 | 2192980 | 2775390 |
| A0AAA9TDX2 | 0 | 127756 | 261337 | 716241 | 111571 | 167134 | 322354 | 146850 |
| A0AAA9TDX3 | 5430860 | 666073 | 2121820 | 622115 | 1317900 | 1172370 | 2206220 | 771279 |
| A0AAA9TEG9 | 155557 | 45328.3 | 127552 | 284671 | 95163 | 233906 | 269144 | 165158 |
| A0AAA9TES1 | 5950860 | 11983200 | 16905700 | 22049400 | 17297900 | 20847900 | 21237100 | 16147900 |
| A0AAA9TEV2 | 17979.1 | 82897.8 | 101413 | 109034 | 83836.9 | 70057.6 | 390318 | 240402 |
| A0AAA9TG85 | 1015400 | 4243200 | 3018410 | 16941600 | 5440470 | 4947420 | 15743600 | 14154100 |
| A0AAA9TGA8 | 91923.6 | 0 | 297712 | 0 | 301993 | 256538 | 654406 | 47681.8 |
| A0AAA9TGC0 | 1548800 | 216503 | 664785 | 41304.4 | 499629 | 265327 | 228772 | 390252 |
| A0AAA9TGJ5 | 384933 | 333955 | 665709 | 553182 | 403294 | 562876 | 456892 | 627826 |
| A0AAA9THP8 | 4578920 | 9667930 | 20539300 | 9577040 | 0 | 0 | 0 | 0 |
| A0AAA9THQ0 | 22833.6 | 0 | 15734.1 | 0 | 0 | 29459.2 | 199536 | 36855.8 |
| A0AAA9TID3 | 116785 | 272668 | 656593 | 155100 | 181836 | 308025 | 526376 | 444169 |
| A0AAA9TIR2 | 3210040 | 452812 | 1107580 | 1194850 | 628359 | 367000 | 1383970 | 689014 |
| A0AAA9TIS4 | 15461800 | 32796400 | 60949300 | 67590200 | 38598400 | 49787600 | 81057600 | 50143800 |
| A0AAA9TJ11 | 81763.9 | 228635 | 385523 | 250855 | 250971 | 354500 | 586995 | 426431 |
| A0AAA9TJ27 | 131153 | 419308 | 389191 | 764820 | 358308 | 480628 | 345745 | 103328 |
| A0AAA9TJ69 | 137027000 | 84368700 | 122687000 | 84657600 | 63405200 | 70412400 | 117932000 | 211363000 |
| A0AAA9TJK9 | 1945230 | 4977490 | 6448210 | 5939080 | 5702000 | 10228800 | 11480400 | 5377840 |
| A0AAA9TK47 | 221874 | 734943 | 822254 | 1189610 | 796059 | 1050230 | 2080330 | 1158210 |
| A0AAA9TKA2 | 1437470 | 3457080 | 6359900 | 2112090 | 3125210 | 5544670 | 8324430 | 3793600 |
| A0AAA9TKS4 | 177731 | 253528 | 423231 | 381363 | 341845 | 439949 | 518048 | 420435 |
| A0AAA9TKS9 | 0 | 0 | 0 | 132778 | 109667 | 0 | 241197 | 159924 |
| A0AAA9TL27 | 62421 | 131097 | 243605 | 964450 | 247404 | 129327 | 92932.3 | 91820.7 |
| A0AAA9TLU8 | 35589.7 | 155611 | 487468 | 476203 | 125627 | 176049 | 310145 | 201892 |
| A0AAA9TM29 | 296198 | 330283 | 821185 | 810636 | 631396 | 1026670 | 1067620 | 613926 |
| A0AAA9TM63 | 1206280 | 3422740 | 1453320 | 2062910 | 2264840 | 1174390 | 1092090 | 606277 |
| A0AAA9TMK4 | 6016200 | 1210530 | 2694660 | 1148780 | 2801960 | 1544390 | 2834040 | 1839120 |
| A0AAA9TN00 | 80316.1 | 164699 | 246627 | 237274 | 163642 | 313140 | 330497 | 274955 |
| A0AAA9TNC9 | 445125 | 597474 | 468862 | 7256360 | 444275 | 675166 | 923270 | 1524050 |
| A0AAA9TNL8 | 0 | 206533 | 489320 | 218034 | 245857 | 210329 | 0 | 95749.6 |
| A0AAA9TNM4 | 193021 | 205725 | 249982 | 856855 | 100958 | 221492 | 329182 | 721814 |
| A0AAA9TNM9 | 126526 | 273396 | 492994 | 1682550 | 614768 | 513076 | 394346 | 499961 |
| A0AAA9TNQ2 | 48757.5 | 226760 | 298702 | 369884 | 227728 | 463847 | 642670 | 580912 |
| A0AAA9TPT4 | 0 | 262272 | 953142 | 684234 | 400663 | 809990 | 416879 | 341889 |
| A0AAA9TQH8 | 2632620 | 4968920 | 5573140 | 31159900 | 3669310 | 1822050 | 5410290 | 11279400 |
| A0AAA9TQL1 | 496084 | 398050 | 721707 | 1607570 | 316727 | 573556 | 262008 | 381422 |
| A0AAA9TQM2 | 0 | 412992 | 462841 | 1540150 | 850495 | 544566 | 817907 | 844721 |
| A0AAA9TQX8 | 389336 | 410755 | 424078 | 852064 | 571436 | 864518 | 1679550 | 196438 |
| A0AAA9TR33 | 23353900 | 4805480 | 14021500 | 4331070 | 8673640 | 6306690 | 12678500 | 8263560 |
| A0AAA9TR35 | 135507 | 577293 | 337699 | 2408970 | 866382 | 980005 | 1220610 | 955837 |
| A0AAA9TRE9 | 41152800 | 6139440 | 15758700 | 4795240 | 14784800 | 10671400 | 17082600 | 13387600 |
| A0AAA9TRV4 | 0 | 543273 | 0 | 9613950 | 293004 | 227015 | 572226 | 1678950 |
| A0AAA9TS64 | 34354.3 | 0 | 522428 | 33872.8 | 204334 | 395852 | 666029 | 247794 |
| A0AAA9TSW5 | 137420 | 257866 | 433794 | 539858 | 367323 | 242633 | 347206 | 281938 |
| A0AAA9TT41 | 1263210 | 3160470 | 3601470 | 3226880 | 2590190 | 3746990 | 3684910 | 3966950 |
| A0AAA9TU07 | 4564240 | 1087460 | 5202180 | 2980070 | 1405580 | 1136490 | 1955050 | 1280460 |
| A0AAA9TU41 | 0 | 157901 | 163192 | 137584 | 64594.8 | 381490 | 813824 | 208977 |
| A0AAA9TUV1 | 0 | 297400 | 358921 | 1962760 | 236153 | 253698 | 764946 | 621346 |
| A0AAA9TVF8 | 38266000 | 13426000 | 26190500 | 45291300 | 24451700 | 18466000 | 23968900 | 24528200 |
| A0AAA9TVX5 | 467421 | 40550.6 | 297778 | 139008 | 52655.3 | 123795 | 285266 | 32783.9 |
| A0AAA9TW15 | 35880000 | 14947000 | 48637800 | 3254310 | 34650300 | 36858100 | 20492000 | 10173300 |
| A0AAA9TWN0 | 2244040 | 256048 | 1260470 | 1060620 | 1048680 | 1012750 | 1216630 | 1009040 |
| A0AAA9TXC4 | 320981 | 64834 | 259584 | 140094 | 182966 | 149399 | 262540 | 232477 |
| A0AAA9TXF5 | 0 | 105062 | 97847.4 | 147060 | 170458 | 137675 | 300351 | 177592 |
| A0AAA9TXX7 | 4915440 | 9738230 | 11191100 | 12261900 | 8201220 | 15765100 | 19033500 | 13588800 |
| A0AAA9TZ12 | 0 | 102841 | 225695 | 163098 | 141590 | 269183 | 424865 | 104266 |
| A0AAA9TZQ7 | 385244 | 791699 | 1024760 | 1028540 | 813232 | 931641 | 1788980 | 1633040 |
| A0AAA9TZS0 | 14221800 | 4874000 | 6039510 | 1911740 | 5479640 | 2992900 | 7023690 | 9024640 |
| A0AAA9U0A3 | 182377 | 204716 | 197365 | 552685 | 226696 | 113270 | 267295 | 401127 |
| A0AAA9U0A7 | 72492.5 | 59798.3 | 401666 | 226409 | 184035 | 300161 | 510798 | 381377 |
| A0AAA9U0F6 | 263117 | 1314480 | 940703 | 2684720 | 2546370 | 1748110 | 1843660 | 1017020 |
| A0AAA9U0R1 | 1496490 | 1048520 | 3020290 | 2465780 | 1527280 | 1284400 | 1439670 | 638786 |
| A0AAA9U0S2 | 422275 | 78653.2 | 271382 | 210455 | 187024 | 134311 | 255999 | 93030.7 |
| A0AAA9U0Z7 | 0 | 35104 | 217586 | 271334 | 90210.6 | 142081 | 242507 | 311361 |
| A0AAA9U1I4 | 146199 | 605978 | 589583 | 468768 | 410896 | 1125060 | 620607 | 341173 |
| A0AAA9U1Y7 | 2243210 | 4240110 | 4668640 | 9724040 | 4239970 | 5770170 | 5187740 | 6080640 |
| A0AAA9U299 | 399690 | 1238560 | 1591850 | 1506160 | 1647440 | 2122230 | 2154980 | 1502660 |
| A0AAF6DLS8 | 964966 | 2070230 | 2159520 | 6498160 | 2066760 | 2609240 | 5087460 | 3672150 |
| A0AAF6DLT6 | 5633530 | 1041980 | 3541300 | 330423 | 2922900 | 1998210 | 997446 | 1750890 |
| A0AAF6DLV1 | 370051 | 1317030 | 3174720 | 2662690 | 1712130 | 1457180 | 2208020 | 1347990 |
| A0AAF6DLY7 | 0 | 0 | 111046 | 268677 | 110831 | 216511 | 277387 | 181969 |
| A0AAF6DM03 | 1011640 | 2086360 | 2292560 | 4454710 | 2091130 | 3317400 | 4933870 | 3910650 |
| A0AAF6DM44 | 0 | 89974.9 | 230178 | 253075 | 183279 | 365405 | 342066 | 162080 |
| A0AAF6DM78 | 1559920 | 2642650 | 3663940 | 4285890 | 3927640 | 3382230 | 3496470 | 5016720 |
| A0AAF6DMB5 | 48272.5 | 52787.6 | 43688.9 | 0 | 65548.5 | 150982 | 272507 | 67559.9 |
| A0AAF6DMC2 | 627039 | 2109430 | 2962440 | 4881710 | 2565330 | 2417120 | 3251950 | 4264730 |
| A0AAF6DMH4 | 502585 | 102202 | 92427.9 | 179948 | 77313.3 | 101017 | 484880 | 0 |
| A0AAF6YH77 | 4490600 | 7998780 | 8160810 | 15737000 | 9718240 | 11952800 | 23693800 | 12863300 |
| A0AAF6YHR8 | 5577630 | 10191700 | 27052900 | 26214300 | 17825500 | 22132700 | 16176900 | 16786800 |
| A0AAF6YJV2 | 0 | 280492 | 269493 | 240296 | 128934 | 601795 | 981909 | 669043 |
| A0AAF6YKW8 | 266318 | 340051 | 604077 | 300682 | 453977 | 540190 | 663773 | 550527 |
| A0AAF6YM87 | 205334 | 225634 | 512521 | 120521 | 467759 | 409897 | 29021.8 | 0 |
| A0AAF6YPV7 | 985123 | 3502140 | 2640580 | 8312550 | 5363620 | 4691930 | 4512070 | 2572260 |
| A0AAF6YQA7 | 302432 | 711950 | 1219570 | 1074460 | 1260210 | 1451650 | 1248980 | 532782 |
| A0AAF6YQI1 | 90699.8 | 127351 | 249366 | 136994 | 133848 | 163636 | 456773 | 153781 |
| A0AAF6YRV5 | 0 | 61277.2 | 125773 | 347986 | 131668 | 280425 | 67892.8 | 113810 |
| A0AAF6YTE2 | 256998 | 728211 | 683847 | 822924 | 609369 | 992984 | 1994380 | 1520710 |
| A0AAF6YU11 | 532110 | 930318 | 1705390 | 1401490 | 1036990 | 1500260 | 1435330 | 797665 |
| A0AAF6YUU7 | 0 | 82843.5 | 38298.2 | 42272.7 | 36321.2 | 108879 | 297347 | 32014.3 |
| A0AAF6YW37 | 1789150 | 2592350 | 3943580 | 2892360 | 2171250 | 3549460 | 5825450 | 2654120 |
| A0AAF6YW73 | 52860.8 | 340185 | 634283 | 285036 | 386562 | 474940 | 658622 | 387727 |
| A0AAF6YWZ4 | 1519390 | 1949870 | 2630640 | 2526310 | 2290380 | 2717650 | 4875310 | 2947640 |
| A0AAF6YY22 | 238949 | 512316 | 585806 | 2860540 | 373415 | 747207 | 1095260 | 1593690 |
| A0AAF6YZK4 | 6547870 | 1285330 | 3591090 | 678055 | 2791880 | 1699090 | 1772110 | 1662750 |
| A0AAF6Z059 | 10367400 | 19829600 | 28024400 | 33525700 | 20698700 | 26445800 | 37602300 | 14803500 |
| A0AAF6Z0U5 | 79915.5 | 277409 | 419114 | 241664 | 309780 | 552657 | 361554 | 259076 |
| A0AAF6Z0W1 | 168554000 | 213023000 | 293553000 | 934537000 | 327888000 | 368234000 | 279967000 | 226851000 |
| A0AAF6Z333 | 154794 | 354288 | 329388 | 1181980 | 342272 | 557134 | 1184020 | 879017 |
| A0AAF6Z3H7 | 3936700 | 833848 | 837826 | 934730 | 1002530 | 579913 | 2136170 | 1021700 |
| A0AAF6Z3Q2 | 341292 | 378867 | 417035 | 2405480 | 162780 | 319169 | 931339 | 591422 |
| A0AAF6Z5S1 | 2677400 | 229383 | 321237 | 0 | 635248 | 200538 | 213194 | 0 |
| A0AAF6Z676 | 0 | 213396 | 181758 | 249206 | 169403 | 229278 | 487857 | 236264 |
| A0AAF6Z6R8 | 870515 | 1108590 | 1865390 | 966287 | 1095890 | 2081550 | 2913140 | 624408 |
| A0AAF6Z6Y5 | 120406 | 949069 | 1426570 | 1905310 | 697956 | 1943050 | 427153 | 880302 |
| A0AAF6Z8P4 | 73053 | 689983 | 759059 | 1449760 | 916742 | 1650490 | 2258980 | 1110910 |
| A0AAF6ZE69 | 373769 | 418688 | 1012250 | 3118480 | 465662 | 626430 | 679243 | 556678 |
| A0AAF6ZEE7 | 39147.9 | 250990 | 643442 | 1175970 | 280311 | 287070 | 0 | 171196 |
| A0AAF6ZGY7 | 2853400 | 2531860 | 166670 | 273950 | 339692 | 101298 | 2903180 | 1341750 |
| A0AAF6ZJE9 | 155538 | 566265 | 139328 | 967715 | 647630 | 561563 | 834047 | 315432 |
| A0AAF6ZJM9 | 173727 | 480489 | 710332 | 960372 | 439618 | 505306 | 1171910 | 621923 |
| A0AAF7A4E2 | 1125690 | 65596.6 | 521629 | 247706 | 359778 | 235884 | 484064 | 229676 |
| A0AAF7A715 | 260304 | 496636 | 1426380 | 6193280 | 570825 | 1661590 | 729752 | 1235590 |
| A0AAF7A7H1 | 1361560 | 231466 | 425750 | 415838 | 482965 | 327487 | 656644 | 584982 |
| A0AAF7A8X1 | 270614 | 359904 | 704390 | 476070 | 551814 | 961746 | 901832 | 401936 |
| A0AAF7A9C9 | 3633120 | 9494520 | 13768200 | 12167500 | 14589600 | 19023600 | 9213090 | 9282420 |
| A0AAF7AAG5 | 765868 | 833351 | 2289060 | 1596970 | 2201150 | 2999360 | 1731620 | 386576 |
| A0AAF7ABE8 | 27404.9 | 119599 | 57846.5 | 424612 | 35138.9 | 89946.5 | 449632 | 257387 |
| A0AAF7AEM3 | 0 | 72829 | 163123 | 422219 | 285075 | 237750 | 519532 | 234006 |
| A0AAF7AF61 | 709338 | 1669130 | 860019 | 1423840 | 873364 | 3078080 | 4620160 | 1685220 |
| A0AAF7AFD7 | 1566010 | 5116620 | 4354940 | 6510920 | 4350010 | 12245600 | 7438050 | 3956840 |
| A0AAF7AGM7 | 289615 | 463764 | 939125 | 921206 | 553442 | 772014 | 1182460 | 691902 |
| A0AAF7AJ81 | 572365 | 568994 | 2616620 | 0 | 492673 | 601355 | 1322190 | 322886 |
| A0AAF7ALE0 | 0 | 38276 | 89929.4 | 0 | 54178.9 | 113599 | 149386 | 0 |
| A0AAF7AP21 | 3182130 | 8014440 | 7948970 | 9640550 | 7669910 | 10795600 | 19307200 | 16210300 |
| A0AAF7AP66 | 145652 | 290934 | 271908 | 654712 | 396307 | 395739 | 554035 | 315545 |
| A1A4I4 | 30473.2 | 63169.1 | 215099 | 272486 | 140850 | 200061 | 316917 | 94260.5 |
| A1A4J1 | 319995 | 1087120 | 1319270 | 1701760 | 672745 | 1265240 | 2196960 | 957547 |
| A1A4R1 | 899425 | 882906 | 1585180 | 360249 | 1240450 | 889044 | 1871980 | 1453060 |
| A1L528 | 3569650 | 10982600 | 14998600 | 14750800 | 10665700 | 14960400 | 16873800 | 16552400 |
| A1L5C6 | 14046400 | 8058500 | 17422300 | 29059500 | 13030900 | 12144100 | 10460000 | 12898800 |
| A2I7N3 | 176921000 | 22363000 | 48448600 | 13579300 | 70166600 | 42202600 | 23344700 | 13931400 |
| A2VDX2 | 0 | 231947 | 1673620 | 94019.4 | 369566 | 1825860 | 699756 | 0 |
| A2VE47 | 2600480 | 709558 | 1524110 | 917538 | 1131250 | 1086030 | 2185870 | 1715100 |
| A4FUA8 | 803372 | 2917050 | 4705150 | 5224660 | 4998210 | 7202880 | 5901840 | 3351920 |
| A4FV32 | 390974 | 728314 | 1160730 | 2709680 | 876763 | 798728 | 557768 | 2138300 |
| A4FV54 | 1628680 | 5760840 | 5544460 | 6642390 | 9702420 | 11463500 | 8657710 | 7232730 |
| A4IFM4 | 1642380 | 3723880 | 4848740 | 6395130 | 4586950 | 4560180 | 3474510 | 5142290 |
| A4IFN6 | 417066 | 768074 | 1033900 | 5183630 | 903885 | 1294000 | 1803500 | 1773760 |
| A4IFU2 | 686134 | 879676 | 1375700 | 1290990 | 818403 | 1215730 | 1121840 | 1370630 |
| A5D795 | 53422.2 | 49777.4 | 0 | 409061 | 0 | 31536.6 | 53220.5 | 131636 |
| A5D7A2 | 426910 | 403082 | 472381 | 404265 | 388112 | 215743 | 788541 | 336848 |
| A5D7D1 | 2148360 | 2703800 | 5122520 | 2759480 | 2485180 | 5565040 | 6075750 | 2754400 |
| A5D7H9 | 9631920 | 18051200 | 29041500 | 38044800 | 21576300 | 21035200 | 29286600 | 28991100 |
| A5D7I4 | 1106850 | 317936 | 334694 | 92730.9 | 113930 | 270773 | 204769 | 0 |
| A5D7L5 | 0 | 84866.2 | 261719 | 913504 | 103016 | 589278 | 75685.7 | 92519.1 |
| A5D7R9 | 1286970 | 1941540 | 2203520 | 3455010 | 1845290 | 2546770 | 2678130 | 2717650 |
| A5D984 | 2687320 | 2517830 | 6406040 | 3155860 | 3804970 | 7017560 | 9923770 | 7905090 |
| A5D989 | 0 | 78533.4 | 171520 | 154443 | 74889 | 353349 | 324538 | 58968.6 |
| A5PJL8 | 1760310 | 582531 | 938375 | 644672 | 787910 | 569700 | 1857390 | 627966 |
| A5PJM4 | 539615 | 1610030 | 3168370 | 3077540 | 2879930 | 4213270 | 2638030 | 2497470 |
| A5PJY9 | 699371 | 1538550 | 3487650 | 2543150 | 2282370 | 2714270 | 3646700 | 1957680 |
| A5PK45 | 4972780 | 1037750 | 1683670 | 864161 | 2181400 | 1734400 | 3352940 | 1440800 |
| A5PK70 | 14124800 | 7422830 | 7198930 | 16354000 | 2540060 | 6974550 | 6923600 | 7035030 |
| A5PKG9 | 83932.5 | 155543 | 295137 | 187172 | 251787 | 264617 | 2435820 | 938514 |
| A5PKL2 | 103822 | 686510 | 446305 | 695535 | 1344180 | 1297250 | 813692 | 183650 |
| A5PKM1 | 103109 | 282299 | 430203 | 1069650 | 466283 | 583606 | 982658 | 808339 |
| A6H6Y1 | 1302900 | 967580 | 6689680 | 1229070 | 2561060 | 484851 | 2200130 | 10667300 |
| A6H7F6 | 49718 | 139312 | 132281 | 224103 | 102860 | 154070 | 402181 | 234432 |
| A6H7G2 | 61319.6 | 456193 | 605194 | 470467 | 396413 | 869946 | 860479 | 251717 |
| A6H7I5 | 50059.3 | 292117 | 307989 | 934331 | 351363 | 376904 | 360147 | 375185 |
| A6QL48 | 1046970 | 491035 | 360096 | 402860 | 165118 | 201529 | 371016 | 236759 |
| A6QLB1 | 0 | 96089.2 | 193411 | 220137 | 121743 | 258298 | 394870 | 283876 |
| A6QLB7 | 619178 | 1560270 | 5011140 | 2188090 | 3465360 | 5966760 | 7513460 | 2095320 |
| A6QLS9 | 18730700 | 34323300 | 45215900 | 63892000 | 44001200 | 55437900 | 80451400 | 71793000 |
| A6QLY4 | 388427 | 646902 | 1460750 | 731001 | 504477 | 674357 | 950384 | 680466 |
| A6QLY9 | 650462 | 2309470 | 1776470 | 11535800 | 1275860 | 631804 | 1382170 | 4695670 |
| A6QNW7 | 95418700 | 104566000 | 56980200 | 144702000 | 68262700 | 103780000 | 60917200 | 30853400 |
| A6QP36 | 426917 | 116324 | 240086 | 160228 | 162680 | 201455 | 422743 | 259828 |
| A6QPQ2 | 23669100 | 2310600 | 3434350 | 1078690 | 3796340 | 2103990 | 1665140 | 1349900 |
| A6QQN6 | 0 | 0 | 0 | 150418 | 198474 | 201585 | 298196 | 300613 |
| A6QQQ3 | 203152 | 396155 | 483335 | 35761.4 | 257507 | 615228 | 555282 | 128472 |
| A6QR60 | 15600500 | 4564560 | 11271200 | 3929850 | 6197700 | 5000830 | 7351900 | 5118880 |
| A7E3Q2 | 157232 | 356159 | 388818 | 1580080 | 1042020 | 655990 | 947458 | 573138 |
| A7E3T1 | 57784 | 828689 | 844537 | 1248030 | 1157440 | 1997700 | 878822 | 875096 |
| A7E3W2 | 10054500 | 625626 | 918948 | 1078110 | 449375 | 776168 | 879905 | 1349500 |
| A7E3W4 | 5409770 | 8939480 | 13905300 | 7728830 | 12182600 | 16319100 | 17815200 | 10492300 |
| A7MB21 | 78493 | 190813 | 179737 | 275749 | 157568 | 270296 | 401391 | 258194 |
| A7MB62 | 849640 | 1532140 | 2403350 | 2151130 | 1539280 | 3846240 | 3648920 | 1801880 |
| A7MBI9 | 40666 | 1604970 | 1325590 | 4218600 | 585363 | 1619590 | 4488790 | 2970090 |
| A7MBJ4 | 515300 | 233751 | 385481 | 869371 | 359345 | 278239 | 543576 | 480995 |
| A7YY28 | 4398090 | 6556380 | 9750920 | 9609320 | 6615600 | 9867950 | 12973900 | 10273600 |
| A8E4P3 | 483147 | 1287950 | 5725290 | 25209.3 | 3087590 | 6998610 | 9346530 | 1754540 |
| A8YXY3 | 2676010 | 940811 | 2190720 | 960665 | 1615960 | 596842 | 1154540 | 1510320 |
| B5TYW4 | 70429.7 | 66073.4 | 164966 | 495412 | 81858.3 | 111758 | 210046 | 260790 |
| C4T7Z1 | 6772080 | 2917880 | 4908930 | 3600680 | 3947210 | 3352670 | 4525170 | 3523980 |
| E1B6Z6 | 35077700 | 11123500 | 7050880 | 7355760 | 4256560 | 6310430 | 7290200 | 7996480 |
| E1B725 | 4047270 | 623957 | 1489510 | 746580 | 1633220 | 844079 | 2062270 | 1816960 |
| E1B726 | 20696100 | 3912750 | 14497600 | 9350350 | 5182490 | 6721100 | 11912100 | 7687960 |
| E1B7G3 | 102671 | 125967 | 75188.9 | 347258 | 0 | 195621 | 178230 | 0 |
| E1B805 | 7951840 | 270902 | 830409 | 268139 | 1237310 | 303782 | 943049 | 197227 |
| E1B9H5 | 3524890 | 1593320 | 2842810 | 1397280 | 2651450 | 1785910 | 2509760 | 5732770 |
| E1B9L0 | 152004000 | 196063000 | 391196000 | 265101000 | 346659000 | 435576000 | 140484000 | 115642000 |
| E1B9R3 | 4534110 | 613349 | 2121560 | 946061 | 1593040 | 860672 | 2292590 | 1531380 |
| E1BA17 | 40015000 | 7072470 | 10844300 | 12894000 | 17673900 | 6577480 | 13043200 | 16732700 |
| E1BA29 | 9448090 | 14327500 | 17939100 | 25108400 | 15333900 | 22653100 | 29242800 | 26408800 |
| E1BAW2 | 146664 | 106463 | 211101 | 1335300 | 109414 | 0 | 0 | 322993 |
| E1BB36 | 2833330 | 394877 | 988106 | 440044 | 885343 | 538057 | 1528500 | 1125200 |
| E1BBP4 | 911113 | 173360 | 351531 | 135973 | 56220.8 | 219051 | 281780 | 190749 |
| E1BBY7 | 24726.5 | 122844 | 229929 | 325234 | 190549 | 265036 | 447611 | 188065 |
| E1BCI3 | 305476 | 3283340 | 2820530 | 16036200 | 2531870 | 2876860 | 8983310 | 9736600 |
| E1BCU8 | 596227 | 968779 | 830174 | 3035480 | 1029910 | 1241890 | 1427980 | 1637180 |
| E1BDE3 | 55651.4 | 251747 | 375711 | 764906 | 235017 | 379778 | 751453 | 409741 |
| E1BDW9 | 0 | 0 | 0 | 0 | 30080.5 | 39435 | 232411 | 0 |
| E1BEL6 | 451669 | 467957 | 1426350 | 2181280 | 1490870 | 2073760 | 1507260 | 2379160 |
| E1BEN2 | 39604.9 | 0 | 206047 | 110863 | 116652 | 30019.1 | 250476 | 60176.4 |
| E1BEN4 | 108990 | 533001 | 961583 | 865056 | 490507 | 845527 | 1575510 | 501789 |
| E1BEV7 | 813616 | 238332 | 533766 | 120544 | 437748 | 152429 | 489687 | 536718 |
| E1BFG0 | 167440 | 502628 | 1152590 | 1126280 | 681951 | 802414 | 1149430 | 559540 |
| E1BG25 | 88404.6 | 1947580 | 2322280 | 12369200 | 2869340 | 1097300 | 3015730 | 6801840 |
| E1BG26 | 19317.6 | 212393 | 368742 | 1043810 | 249142 | 328288 | 697047 | 703746 |
| E1BGA2 | 137490 | 331685 | 400195 | 2721630 | 413941 | 700541 | 900408 | 404377 |
| E1BGB1 | 0 | 50668.1 | 0 | 421150 | 135231 | 113648 | 141793 | 84931 |
| E1BGX8 | 93449800 | 27880200 | 36183000 | 20225900 | 63668000 | 30646500 | 53030600 | 31229600 |
| E1BH06 | 18627200 | 5570650 | 7671790 | 5717090 | 8524220 | 6278290 | 3470900 | 4282560 |
| E1BI14 | 44570.5 | 227242 | 182721 | 481847 | 0 | 121651 | 264812 | 602056 |
| E1BIX3 | 2199520 | 5311910 | 3748220 | 20066400 | 7232480 | 7688870 | 6650260 | 5782340 |
| E1BJ18 | 5333960 | 4628740 | 15148600 | 54587600 | 9672060 | 17650000 | 15294500 | 15594300 |
| E1BJ75 | 2879190 | 728739 | 1702540 | 549580 | 1607610 | 1085240 | 2255690 | 2186130 |
| E1BJQ7 | 615552 | 1649170 | 2170330 | 2690640 | 1232640 | 2052670 | 3006290 | 2254520 |
| E1BJV0 | 2863840 | 8545300 | 7853850 | 11505300 | 7476750 | 18053300 | 15489600 | 10241100 |
| E1BJW2 | 735664 | 1532260 | 3383850 | 2258010 | 1731890 | 1657200 | 3951200 | 3944180 |
| E1BK52 | 173496 | 209684 | 560118 | 506623 | 280382 | 543992 | 802957 | 273128 |
| E1BK71 | 334806 | 976807 | 1557520 | 2592650 | 804568 | 1734300 | 2822540 | 2484930 |
| E1BKM4 | 58231500 | 144562000 | 93285500 | 351105000 | 180749000 | 177847000 | 154969000 | 242665000 |
| E1BKQ2 | 37526 | 60932.1 | 0 | 99461.9 | 50395.7 | 117986 | 420174 | 0 |
| E1BLF1 | 329010 | 965201 | 1979220 | 1954600 | 500619 | 1057790 | 3629010 | 2436500 |
| E1BM92 | 287786 | 298981 | 215393 | 891806 | 96025.1 | 91753.1 | 491006 | 419520 |
| E1BMM8 | 1263470 | 185922 | 481268 | 225796 | 404702 | 306941 | 395472 | 353343 |
| E1BNC4 | 159076 | 39034 | 87258.7 | 0 | 47332.4 | 55015.2 | 150079 | 245846 |
| E1BNE5 | 0 | 38233.4 | 0 | 124999 | 0 | 136044 | 758685 | 161440 |
| E1BNH2 | 485968 | 770200 | 2660620 | 1178560 | 1152090 | 1419630 | 1287600 | 945646 |
| E1BNW4 | 286762 | 1446830 | 1410410 | 6396260 | 532128 | 1124370 | 2781080 | 1873980 |
| E1BPI3 | 87538.3 | 326412 | 634018 | 1618900 | 327442 | 540339 | 1109940 | 600634 |
| E1BQ28 | 78733.4 | 184581 | 242639 | 2005740 | 131361 | 381553 | 2882370 | 859367 |
| E1BQ32 | 120952 | 386350 | 812128 | 957260 | 489510 | 770298 | 643141 | 591643 |
| F1MBB7 | 45767.6 | 501917 | 488852 | 747856 | 465242 | 658324 | 463796 | 569638 |
| F1MBW3 | 386039 | 1079430 | 1257270 | 1785730 | 984149 | 2480330 | 1763360 | 1785850 |
| F1MC11 | 3710500 | 5301740 | 10358900 | 4432510 | 21485900 | 11756000 | 12180000 | 8618770 |
| F1MCX1 | 16952300 | 14131600 | 16073800 | 80916700 | 9495570 | 13121300 | 16502600 | 28876800 |
| F1MD24 | 6324310 | 13356800 | 19064200 | 26221700 | 12604800 | 17376100 | 27196800 | 28108200 |
| F1MD47 | 563586 | 1391320 | 2207150 | 2074870 | 1523310 | 2119790 | 3694070 | 2639980 |
| F1MD77 | 748224 | 495521 | 552692 | 1895610 | 608965 | 281775 | 373148 | 858599 |
| F1MD94 | 780543 | 1268550 | 3779140 | 1231520 | 800076 | 4201390 | 1754150 | 2109820 |
| F1MDD6 | 61706.5 | 189046 | 361631 | 291419 | 234768 | 496481 | 512854 | 310747 |
| F1MDQ6 | 0 | 111916 | 174006 | 487335 | 259443 | 310728 | 1519780 | 857559 |
| F1MET0 | 8598260 | 1080520 | 3602220 | 678221 | 2835490 | 1541190 | 3233680 | 2175720 |
| F1MEX9 | 154464 | 627931 | 278425 | 780700 | 205739 | 559919 | 1624000 | 1232100 |
| F1MFI4 | 10779300 | 1550970 | 9722480 | 3019330 | 13075300 | 824309 | 2900530 | 4073740 |
| F1MFW9 | 639064 | 2065770 | 3578500 | 270787 | 1475520 | 1080780 | 1678690 | 1676720 |
| F1MGC2 | 334568 | 1002600 | 1794640 | 298171 | 0 | 248887 | 2305010 | 1218780 |
| F1MGR1 | 112712000 | 229740000 | 217551000 | 215955000 | 237945000 | 319272000 | 327212000 | 199948000 |
| F1MHC2 | 2461360 | 8046280 | 8854360 | 6172870 | 8679740 | 10272800 | 19139100 | 9081060 |
| F1MHP4 | 447689 | 596180 | 763629 | 1468800 | 809970 | 978537 | 1443840 | 981200 |
| F1MHQ1 | 588929 | 903230 | 1692380 | 1875030 | 668599 | 1023480 | 776598 | 435607 |
| F1MHS5 | 201739 | 271038 | 1323460 | 1111370 | 372151 | 1278550 | 2496670 | 639565 |
| F1MIH9 | 1705330 | 169002 | 523092 | 496727 | 332016 | 299056 | 371877 | 240897 |
| F1MJ96 | 0 | 93406.6 | 41808.1 | 244037 | 76792.3 | 155073 | 150929 | 64268.4 |
| F1MJK3 | 5380870 | 513121 | 1425250 | 81407.3 | 835092 | 719303 | 181884 | 353334 |
| F1MJP7 | 137501 | 365035 | 503091 | 1230020 | 160833 | 653511 | 1158910 | 863083 |
| F1MJQ1 | 359249 | 735507 | 955586 | 1559960 | 1055820 | 1324140 | 2140730 | 1819730 |
| F1MJQ3 | 5961650 | 1766610 | 3426890 | 1378890 | 2226820 | 1513560 | 1728850 | 1446290 |
| F1MJU5 | 500074 | 957650 | 5510650 | 635294 | 1776850 | 3410680 | 3495590 | 5605060 |
| F1MJV6 | 2956720 | 2618920 | 2613310 | 7934520 | 2024810 | 3624690 | 2553580 | 6914590 |
| F1MJW4 | 4734090 | 929217 | 2742050 | 1481560 | 1862540 | 1501540 | 2694470 | 1416190 |
| F1MK52 | 28258700 | 46468400 | 49870200 | 169081000 | 33789600 | 44308000 | 28058300 | 79003400 |
| F1MKU3 | 294472 | 735009 | 1698000 | 1758540 | 1470110 | 1924900 | 3145180 | 868565 |
| F1ML02 | 1060280 | 1097420 | 1158140 | 3549630 | 1310310 | 2118250 | 1327790 | 2164530 |
| F1MLA4 | 258012 | 481634 | 819413 | 723137 | 488185 | 602034 | 1349460 | 667975 |
| F1MLI8 | 2064550 | 4203290 | 8743310 | 8649420 | 3315780 | 3573140 | 12500700 | 8354680 |
| F1MLZ1 | 19327800 | 30371300 | 32215400 | 6525590 | 35437400 | 36767600 | 8314010 | 3560850 |
| F1MM13 | 7439850 | 979316 | 2281360 | 2090290 | 3156590 | 1714640 | 3949160 | 3180020 |
| F1MM32 | 12049300 | 2049580 | 4650140 | 2249810 | 2101500 | 1603490 | 6388120 | 4804180 |
| F1MM83 | 83806.1 | 730524 | 907661 | 1107770 | 939616 | 1178310 | 2465340 | 1308630 |
| F1MM86 | 1689570 | 260154 | 730148 | 371016 | 370692 | 201582 | 285252 | 1266300 |
| F1MMV6 | 70067.2 | 322218 | 326631 | 559506 | 240140 | 358930 | 592703 | 276119 |
| F1MNI4 | 1000750 | 1548630 | 2611340 | 3588970 | 1903980 | 1996360 | 2861600 | 1837300 |
| F1MNN6 | 720927 | 701267 | 1140560 | 929332 | 957891 | 1647080 | 3413930 | 210212 |
| F1MNN7 | 21188900 | 4883800 | 5583180 | 11242600 | 2651770 | 10565200 | 8423150 | 6711320 |
| F1MNV5 | 151529 | 0 | 0 | 100679 | 247408 | 213725 | 437695 | 274698 |
| F1MPI4 | 3926480 | 635178 | 1758810 | 695133 | 1212580 | 828347 | 1329340 | 903659 |
| F1MPP2 | 1319890 | 222331 | 596586 | 516041 | 281552 | 363394 | 577701 | 324335 |
| F1MPZ9 | 47225.7 | 313812 | 252436 | 100037 | 242818 | 598146 | 140652 | 113968 |
| F1MQD1 | 0 | 0 | 35246.3 | 0 | 62490 | 46584.2 | 62258.8 | 54588.9 |
| F1MRM8 | 6677570 | 3465810 | 4825720 | 114366000 | 13387500 | 3826240 | 19594800 | 15298300 |
| F1MS05 | 5147320 | 9675190 | 12796100 | 10616100 | 8416100 | 13332300 | 17066000 | 10863300 |
| F1MS08 | 174577 | 1886210 | 1259780 | 2785980 | 1650280 | 3094710 | 1871730 | 2311230 |
| F1MSI2 | 82671.9 | 415409 | 310365 | 2960440 | 214695 | 224817 | 300164 | 535117 |
| F1MSN2 | 133719 | 790801 | 886509 | 1251800 | 682092 | 1476690 | 495099 | 665181 |
| F1MSZ6 | 2535330 | 462643 | 1077920 | 1080300 | 804137 | 755234 | 1317350 | 1176990 |
| F1MT69 | 965123 | 2297920 | 2088320 | 7779210 | 2440860 | 2684650 | 1918630 | 2508640 |
| F1MTR4 | 658752 | 418894 | 733940 | 3981980 | 1304030 | 1093190 | 510122 | 3431390 |
| F1MU79 | 0 | 87079.7 | 120094 | 194654 | 120406 | 165596 | 631687 | 180467 |
| F1MU85 | 3803420 | 6598530 | 6357410 | 37289900 | 9384200 | 11399000 | 10983700 | 10747700 |
| F1MUP1 | 60366200 | 33404100 | 144075000 | 66695800 | 64567600 | 120272000 | 36620700 | 61868900 |
| F1MUP9 | 2893000 | 5233190 | 5636820 | 4075240 | 4004380 | 4775050 | 8069930 | 4260890 |
| F1MUT3 | 48471 | 2845930 | 1845120 | 1465390 | 1841780 | 3815130 | 4150080 | 3125690 |
| F1MVJ8 | 1398640 | 828545 | 3351660 | 1380800 | 467392 | 5051800 | 28876500 | 3864730 |
| F1MVK1 | 2788690 | 868303 | 838941 | 1667600 | 970326 | 1153370 | 874659 | 1321990 |
| F1MW13 | 972501 | 999268 | 1994200 | 3631670 | 1320780 | 2717760 | 2109690 | 2689000 |
| F1MW44 | 741216 | 142576 | 764262 | 140757 | 295479 | 262801 | 481745 | 393025 |
| F1MWI1 | 588435 | 515454 | 878968 | 132332 | 177984 | 111954 | 0 | 126219 |
| F1MWQ2 | 35366.3 | 224792 | 2207530 | 163145 | 237195 | 895728 | 1124390 | 708507 |
| F1MWX3 | 1886570 | 641462 | 2958880 | 9321420 | 3002070 | 2640360 | 366520 | 330475 |
| F1MX50 | 3627930 | 845886 | 3033740 | 1388260 | 1758400 | 1240570 | 2628760 | 2182830 |
| F1MX87 | 1474100 | 150551 | 667448 | 164732 | 125573 | 111174 | 74263.9 | 641273 |
| F1MXD2 | 0 | 171109 | 139070 | 366668 | 97422.4 | 149298 | 98149.7 | 110027 |
| F1MXG6 | 0 | 3358770 | 249968 | 365374 | 67894.5 | 235325 | 22049.8 | 300825 |
| F1MXH7 | 79543.8 | 82139.8 | 206269 | 754540 | 93491.7 | 50657.1 | 196509 | 260592 |
| F1MY58 | 808327 | 2250640 | 2609230 | 7084080 | 2531490 | 3331210 | 4093550 | 4370800 |
| F1MY82 | 0 | 17462.4 | 18844.5 | 82361.2 | 0 | 22033.9 | 208672 | 61723.8 |
| F1MY85 | 64253900 | 8568820 | 15567700 | 18299500 | 19183800 | 14114700 | 18616600 | 18280200 |
| F1MYQ3 | 23503 | 202340 | 441394 | 57910.6 | 85221 | 256838 | 458156 | 176393 |
| F1MYX5 | 0 | 22190.5 | 563478 | 0 | 483332 | 788220 | 919792 | 633684 |
| F1MZ71 | 315185 | 775898 | 1044200 | 3789300 | 987754 | 993571 | 1445520 | 2347540 |
| F1MZA0 | 21036000 | 7668290 | 16952400 | 10736000 | 17784400 | 6841750 | 15827600 | 10582300 |
| F1MZQ4 | 1077350000 | 3102970000 | 2534900000 | 3806170000 | 2829770000 | 3380010000 | 4396320000 | 4379020000 |
| F1MZV2 | 909100 | 3977600 | 3095190 | 13331300 | 6233660 | 5332950 | 3756810 | 2206490 |
| F1N026 | 5502350 | 9081120 | 17885400 | 35883100 | 18031100 | 7401830 | 21795000 | 11927800 |
| F1N036 | 15016200 | 3355400 | 8641300 | 2517700 | 7872330 | 3663360 | 11299300 | 7536100 |
| F1N076 | 27881800 | 6112070 | 17722100 | 5385900 | 14250900 | 6927600 | 16529400 | 17894100 |
| F1N0I3 | 6372720 | 1096630 | 1834690 | 3232670 | 1240120 | 1528930 | 2562150 | 1898880 |
| F1N0J3 | 620881 | 1329320 | 1796350 | 2221320 | 1244270 | 2017110 | 1666990 | 1632640 |
| F1N0M7 | 11458400 | 2284210 | 7979510 | 1625800 | 3507800 | 3388460 | 6820970 | 5278040 |
| F1N102 | 10649000 | 1386890 | 3298410 | 935134 | 1842230 | 1297800 | 2386620 | 2332660 |
| F1N152 | 9843170 | 2379460 | 8447080 | 3739450 | 3686480 | 3820110 | 7945690 | 6372410 |
| F1N261 | 1212380 | 3669990 | 3427720 | 13695900 | 3513470 | 5407520 | 7134260 | 7303440 |
| F1N290 | 100917 | 482800 | 571941 | 424445 | 273121 | 655068 | 850361 | 516250 |
| F1N2K1 | 929513 | 211214 | 644158 | 250180 | 468910 | 326243 | 727322 | 686247 |
| F1N332 | 43611 | 345026 | 270268 | 1376160 | 442392 | 482201 | 551682 | 383793 |
| F1N3A1 | 5832860 | 3703350 | 2980590 | 1889120 | 2694480 | 1425300 | 4958850 | 5724680 |
| F1N3P2 | 0 | 73085.4 | 27816.2 | 133203 | 82883.1 | 223718 | 434224 | 146417 |
| F1N412 | 101736 | 324979 | 884668 | 843631 | 390672 | 511627 | 1393700 | 372742 |
| F1N4K7 | 878887 | 1603950 | 1642600 | 3529360 | 1703430 | 2184390 | 1670900 | 1682810 |
| F1N4W0 | 3118400 | 351157 | 1156990 | 815583 | 1340080 | 417877 | 1760340 | 767002 |
| F1N5T0 | 624594 | 211471 | 547695 | 223652 | 235174 | 163519 | 234093 | 164903 |
| F1N5T9 | 561445 | 1419990 | 140743 | 9934820 | 3231420 | 1550420 | 163223 | 3290390 |
| F1N5W5 | 689970 | 1413790 | 1763660 | 1796140 | 1265930 | 2417030 | 5069210 | 1701660 |
| F1N5Z0 | 4756870 | 8485820 | 9703830 | 30853900 | 6745580 | 9126080 | 5427710 | 7928920 |
| F1N647 | 653804 | 4474920 | 7121040 | 7716620 | 2247100 | 4983500 | 11015300 | 5725270 |
| F1N6D4 | 27246800 | 147105000 | 113839000 | 363189000 | 89160900 | 137888000 | 246700000 | 302205000 |
| F1N6H1 | 9891180 | 19677700 | 34938900 | 108767000 | 23160100 | 15538800 | 27230900 | 47833200 |
| F1N6W6 | 0 | 193365 | 179504 | 760575 | 314732 | 341712 | 1038460 | 428116 |
| F1N780 | 558635 | 123809 | 379208 | 183107 | 245316 | 131379 | 274191 | 134554 |
| F1N7C5 | 233245 | 328563 | 933114 | 475078 | 2621540 | 4363270 | 3453200 | 3305040 |
| F1N7H0 | 67682.6 | 33859.5 | 46040.6 | 237876 | 125180 | 154303 | 315656 | 155270 |
| F1N7I6 | 270223 | 595414 | 3005390 | 1338460 | 1108530 | 1393600 | 1512960 | 864369 |
| F1N7R5 | 0 | 40378.6 | 31656.8 | 156743 | 90634.7 | 85535.2 | 100688 | 88278.1 |
| F1N7T1 | 8147020 | 13482800 | 16586200 | 26212200 | 15409400 | 27644900 | 32531800 | 32401300 |
| F2Z4F5 | 90928.3 | 240817 | 372614 | 264611 | 308286 | 347692 | 606243 | 487778 |
| F2Z4I1 | 466664 | 1056380 | 1279590 | 1395300 | 1116610 | 1167720 | 1714730 | 2259770 |
| F6PWS3 | 1025330 | 3101580 | 3200020 | 3212660 | 2448400 | 3029300 | 3284800 | 2777900 |
| F6PZI3 | 3008300 | 11783000 | 15545800 | 43699100 | 12468000 | 25372900 | 13071000 | 20234200 |
| F6PZZ7 | 4755160 | 10285500 | 8688380 | 48448700 | 14039800 | 17597200 | 21205900 | 11220100 |
| F6Q751 | 438263 | 1624100 | 3280130 | 1880330 | 1538690 | 2681970 | 4656540 | 2723640 |
| F6Q9I3 | 464150 | 214577 | 155847 | 846971 | 408196 | 302639 | 279958 | 339312 |
| F6QAZ2 | 946113 | 653869 | 687886 | 3590960 | 761962 | 1067790 | 1402710 | 2665180 |
| F6QE93 | 5634080 | 1319450 | 3206980 | 1397780 | 2005460 | 1783260 | 3877930 | 2999080 |
| F6QEU6 | 1690440 | 2137480 | 4449590 | 4902630 | 3212710 | 3925940 | 4989620 | 2702810 |
| F6QJE8 | 32325.9 | 233748 | 327099 | 309189 | 224618 | 400462 | 910145 | 454345 |
| F6QK60 | 18234.3 | 33099.8 | 114205 | 204476 | 128606 | 155326 | 370136 | 259336 |
| F6QKI7 | 1029240 | 841056 | 1324240 | 187701 | 1296400 | 816865 | 436157 | 954526 |
| F6QPB9 | 316829 | 245336 | 5107920 | 360824 | 2206310 | 4530450 | 1487920 | 2665380 |
| F6QPI1 | 433360 | 280610 | 167705 | 1152220 | 128409 | 71025.4 | 661533 | 383708 |
| F6QQD9 | 261555 | 316435 | 363252 | 90450.9 | 211891 | 278076 | 28826.1 | 0 |
| F6QZ79 | 33050.5 | 225702 | 581536 | 215208 | 253540 | 377702 | 513098 | 205790 |
| F6R1P1 | 8934460 | 1230960 | 4692240 | 2495700 | 3037900 | 1829150 | 4489980 | 2354110 |
| F6R3N4 | 14145400 | 23014900 | 54605700 | 12968700 | 25339100 | 21645200 | 30765300 | 24863900 |
| F6R4P6 | 58331000 | 9655740 | 37200800 | 8479690 | 23063200 | 14798900 | 23011600 | 21034700 |
| F6RAW2 | 146961 | 137746 | 151976 | 700551 | 135656 | 114744 | 152984 | 213733 |
| F6RFP6 | 7383030 | 20398900 | 27023600 | 70321000 | 25691700 | 33941900 | 33638700 | 32396600 |
| F6RM11 | 172350 | 621116 | 920746 | 1278210 | 927279 | 842916 | 1070430 | 893754 |
| F6RQC9 | 2897860 | 6240050 | 10029400 | 10265200 | 8040890 | 14732300 | 21856200 | 12451100 |
| F6RR15 | 38496.7 | 395634 | 444650 | 954586 | 384951 | 843128 | 179512 | 0 |
| F6RS49 | 66101.7 | 135603 | 356664 | 0 | 309064 | 304460 | 0 | 0 |
| F6RU06 | 69403900 | 23709600 | 68907800 | 6534660 | 56559700 | 51294400 | 39278100 | 22299200 |
| F6S0B8 | 1938490 | 622571 | 1554790 | 618185 | 947836 | 827671 | 1655450 | 1038830 |
| G1K122 | 2252140 | 287851 | 975885 | 285628 | 915014 | 597060 | 1014350 | 338622 |
| G3MWP1 | 281323 | 178778 | 898856 | 268365 | 296371 | 473431 | 1033330 | 419289 |
| G3MWT2 | 1158550 | 2156290 | 10020200 | 2934510 | 5862010 | 5207190 | 6107190 | 27569400 |
| G3MWU7 | 0 | 0 | 78842.2 | 0 | 0 | 80899.8 | 60097.9 | 0 |
| G3MXB5 | 172088000 | 262741000 | 142211000 | 56486800 | 68714400 | 302726000 | 96334100 | 97470100 |
| G3MXZ7 | 7430080 | 1078170 | 2737420 | 1919730 | 2166220 | 1638630 | 3064840 | 2468410 |
| G3MYD9 | 55234 | 97059.8 | 1234000 | 142070 | 591389 | 1842400 | 1936950 | 1279410 |
| G3MZ88 | 560330 | 491199 | 677444 | 110756 | 528552 | 485124 | 351783 | 453457 |
| G3MZH7 | 809217 | 1993170 | 2192880 | 2061810 | 2858920 | 6530140 | 2720490 | 4132940 |
| G3N072 | 0 | 0 | 0 | 145437 | 99918.2 | 73853.8 | 33922.9 | 0 |
| G3N0K3 | 48889.7 | 176464 | 272055 | 166219 | 185383 | 412696 | 377517 | 222159 |
| G3N0S9 | 804632 | 138667 | 326023 | 644084 | 296986 | 303873 | 376630 | 601853 |
| G3N0V0 | 62385200 | 43935500 | 28568500 | 27010400 | 8300380 | 7285790 | 11443300 | 6733460 |
| G3N0V2 | 16602000 | 18860800 | 47913900 | 12881800 | 35493700 | 27142700 | 27060400 | 34602800 |
| G3N188 | 3122910 | 662066 | 808953 | 678923 | 1196320 | 3883630 | 1119250 | 176132 |
| G3N1H5 | 653108 | 312210 | 252817 | 533480 | 100774 | 443254 | 171531 | 288503 |
| G3N2D8 | 120111000 | 146948000 | 214424000 | 567444000 | 127570000 | 209713000 | 195294000 | 285796000 |
| G3N2F2 | 2019410 | 301224 | 874760 | 305639 | 589505 | 343734 | 587186 | 545421 |
| G3N342 | 1208660 | 2116310 | 1458510 | 840095 | 1261980 | 1429030 | 4432360 | 1285220 |
| G3X696 | 0 | 77691.2 | 315873 | 478884 | 95462.2 | 128891 | 408072 | 204619 |
| G3X6G8 | 24321700 | 6605430 | 20572000 | 11600000 | 13859600 | 9436800 | 19404800 | 10128700 |
| G3X6I7 | 771382 | 1292970 | 1664880 | 1690100 | 1182780 | 1727400 | 2079190 | 1801860 |
| G3X6N3 | 17686300 | 5529590 | 9140690 | 5789180 | 5089000 | 4780740 | 9437250 | 4080630 |
| G3X743 | 105340 | 176874 | 308559 | 280437 | 212205 | 373292 | 458493 | 230200 |
| G3X745 | 1054490 | 556277 | 422761 | 3068120 | 609054 | 433076 | 439956 | 978975 |
| G5E513 | 7225930000 | 8115950000 | 4116420000 | 5148330000 | 3811950000 | 5213240000 | 3583720000 | 3550230000 |
| G5E531 | 69064.9 | 198006 | 234029 | 0 | 48294.6 | 225104 | 300192 | 229527 |
| G5E589 | 1127960 | 342287 | 837869 | 540687 | 922667 | 1079840 | 1449260 | 589189 |
| G5E5A8 | 20054100 | 8019040 | 14936700 | 26809100 | 6534410 | 3165510 | 9765080 | 7547600 |
| G5E5C8 | 853695 | 1651370 | 3664010 | 1632290 | 3004950 | 3577070 | 2869690 | 1561250 |
| G5E5M9 | 944961 | 105684 | 777255 | 139931 | 368226 | 250765 | 421266 | 204739 |
| G5E5T5 | 8801560 | 6804390 | 6336980 | 5919050 | 4127790 | 2218220 | 2174440 | 6288740 |
| G5E5V1 | 93842600 | 99322700 | 123071000 | 144488000 | 54960900 | 64847600 | 58612800 | 87287200 |
| G5E641 | 424480 | 1013180 | 1291300 | 5773960 | 1539180 | 812391 | 4902070 | 2104560 |
| G5E6P3 | 6674780 | 11691700 | 13299400 | 18732900 | 13083900 | 19643900 | 30332300 | 24544600 |
| H7BWW0 | 17624.2 | 36982.2 | 75009.5 | 121326 | 129971 | 274629 | 365847 | 101262 |
| O18836 | 2788920 | 1012880 | 3731370 | 1041160 | 525894 | 734691 | 955920 | 1289770 |
| O18875 | 2816100 | 3710420 | 5619220 | 14078700 | 5205220 | 6019410 | 7452430 | 6397770 |
| O19131 | 859429 | 786005 | 569099 | 680526 | 179964 | 484261 | 1030410 | 769165 |
| O46375 | 8457020 | 1812580 | 3972700 | 1349070 | 2836880 | 1304830 | 1342720 | 2023440 |
| O46414 | 1549220 | 715853 | 1501040 | 584370 | 1579810 | 1189120 | 968200 | 1451850 |
| O46563 | 63044.6 | 114240 | 298607 | 0 | 192324 | 303986 | 506289 | 193582 |
| O46631 | 777501 | 942605 | 973910 | 5716250 | 1321400 | 2073870 | 1936790 | 2224140 |
| O77834 | 3090910 | 5185080 | 8539240 | 7379410 | 5809380 | 8523010 | 10150200 | 7706690 |
| O97680 | 735345 | 2389670 | 4318200 | 2695150 | 3244030 | 1650610 | 2700720 | 3670510 |
| O97764 | 730757 | 1119910 | 2687520 | 1626790 | 1452490 | 2093780 | 1912330 | 1217240 |
| P00442 | 517044 | 1867850 | 2847580 | 3452200 | 4120460 | 1836680 | 1647390 | 2216730 |
| P00515 | 669248 | 504306 | 1133920 | 707395 | 636179 | 951039 | 1560910 | 1143390 |
| P00711 | 9118310 | 5727680 | 11113100 | 8780840 | 10013900 | 4516040 | 11021600 | 8505180 |
| P00727 | 4595280 | 9940590 | 17626400 | 13441700 | 14261800 | 17678000 | 24198900 | 20695300 |
| P00829 | 842909 | 1268170 | 1696060 | 837649 | 1206590 | 833168 | 431235 | 3538510 |
| P01017 | 11754200 | 2710130 | 5959750 | 868062 | 3498030 | 4045110 | 4356120 | 6877300 |
| P01045 | 3548280 | 1271800 | 2544960 | 952096 | 1595760 | 1515480 | 2047780 | 1492840 |
| P01888 | 16012100 | 35418900 | 22958600 | 840898 | 53337500 | 11821700 | 2366720 | 1545100 |
| P01966 | 51069900 | 5085760 | 50312700 | 29284400 | 22358900 | 2669350 | 7377610 | 30511400 |
| P02070 | 121656000 | 5061140 | 103326000 | 81397900 | 25324200 | 4535560 | 16943900 | 56778100 |
| P02584 | 2635930 | 6137540 | 9917580 | 4838940 | 6420380 | 5843480 | 7640770 | 5950320 |
| P02662 | 3871070000 | 724982000 | 1806650000 | 710027000 | 1206950000 | 607375000 | 1704010000 | 1221780000 |
| P02663 | 484626000 | 270075000 | 348637000 | 143159000 | 260208000 | 92625200 | 434777000 | 372853000 |
| P02754 | 266341000 | 109551000 | 210608000 | 91485500 | 208784000 | 45697600 | 162021000 | 166229000 |
| P02769 | 56627800 | 27451800 | 46692800 | 25477900 | 40693700 | 40859600 | 59472500 | 30100300 |
| P04272 | 35012200 | 74790000 | 179461000 | 138901000 | 124987000 | 149509000 | 97556600 | 73328500 |
| P05786 | 0 | 1002630 | 339324 | 143258 | 201465 | 215899 | 331248 | 281062 |
| P06623 | 1250520 | 1703590 | 2525200 | 3492620 | 2377800 | 3902510 | 5674750 | 3848730 |
| P08037 | 183600000 | 35179900 | 97246600 | 37474400 | 54062200 | 41283000 | 93821800 | 65560300 |
| P08169 | 294967 | 397335 | 810700 | 1854980 | 385524 | 197578 | 370539 | 308189 |
| P08728 | 826061 | 1615750 | 3152510 | 1540160 | 2236800 | 2232050 | 1896850 | 1542510 |
| P0CH28 | 103470000 | 229620000 | 221340000 | 367400000 | 335291000 | 294463000 | 130002000 | 241014000 |
| P10152 | 407501 | 724777 | 402120 | 226128 | 265703 | 39546.3 | 84983.5 | 452580 |
| P10790 | 10412800 | 43429200 | 35497500 | 62385400 | 29650400 | 23627200 | 189967000 | 156425000 |
| P11017 | 11133100 | 16949500 | 21248300 | 21539000 | 14945200 | 26250300 | 37813300 | 32166200 |
| P11151 | 5051160 | 1187970 | 2887360 | 2256720 | 2214110 | 1481340 | 5271710 | 3440930 |
| P12624 | 546901 | 1168080 | 2802480 | 1559010 | 4419590 | 7367360 | 8179190 | 3491580 |
| P12763 | 778346 | 177472 | 360474 | 229872 | 208214 | 227581 | 218088 | 77045 |
| P13135 | 97335.1 | 131159 | 282461 | 191567 | 258389 | 347444 | 484555 | 340453 |
| P13696 | 3077970 | 5748200 | 8158780 | 14189200 | 6331800 | 9598590 | 19461900 | 8921180 |
| P13752 | 15798600 | 20623600 | 52462800 | 59011200 | 72464100 | 73061400 | 38636300 | 62976700 |
| P13753 | 10208400 | 7258150 | 17833100 | 42116400 | 13410500 | 21450700 | 16546700 | 749989 |
| P15396 | 401665 | 370095 | 693932 | 1103090 | 729233 | 854075 | 11443500 | 3035680 |
| P15467 | 544515 | 287680 | 193729 | 239507 | 75467.2 | 27782.2 | 409117 | 130070 |
| P15497 | 172034000 | 60826100 | 161208000 | 82725100 | 104968000 | 78805500 | 147901000 | 157931000 |
| P17248 | 4538270 | 10860900 | 18920900 | 13164600 | 15046500 | 18401800 | 34743100 | 14600600 |
| P18203 | 0 | 75332.5 | 124184 | 1791500 | 100686 | 83905.5 | 209546 | 151962 |
| P18892 | 13686100 | 30580300 | 82371.4 | 32287500 | 384522 | 42679800 | 52452200 | 47790600 |
| P19120 | 113077000 | 208884000 | 228386000 | 609454000 | 384630000 | 377581000 | 313549000 | 320053000 |
| P19803 | 3404170 | 7105540 | 9332860 | 11596900 | 7886800 | 11190900 | 21408900 | 14546100 |
| P22226 | 6066860 | 3234120 | 11054000 | 2832520 | 3712020 | 8331310 | 34260100 | 6976610 |
| P22444 | 324581 | 59661.9 | 147571 | 26917.6 | 122836 | 83977 | 228888 | 0 |
| P25417 | 984405 | 1417480 | 1855360 | 3305880 | 3154760 | 1872070 | 3100670 | 3649360 |
| P26201 | 403890000 | 1126580000 | 1057580000 | 2714250000 | 1041260000 | 1002680000 | 1383830000 | 2246290000 |
| P26452 | 188877 | 408633 | 723505 | 472123 | 614747 | 813533 | 1356600 | 214322 |
| P27479 | 78999.3 | 504902 | 104087 | 568269 | 321514 | 182882 | 2417950 | 1982200 |
| P28782 | 53371.2 | 58623.2 | 808497 | 184879 | 315873 | 681873 | 408925 | 27175.7 |
| P28801 | 1426290 | 1331890 | 3238270 | 1822670 | 1769060 | 4195080 | 4418220 | 2581300 |
| P31404 | 506644 | 869428 | 1542910 | 668624 | 941171 | 1420570 | 1559030 | 1105380 |
| P31408 | 247721 | 448979 | 1408140 | 1088540 | 919682 | 1039450 | 2077080 | 882800 |
| P31754 | 21661.7 | 209807 | 721062 | 565097 | 92465.6 | 418584 | 665920 | 150784 |
| P32749 | 187772 | 0 | 0 | 681234 | 47994 | 169280 | 0 | 0 |
| P33672 | 206151 | 298432 | 613294 | 320794 | 346116 | 574086 | 551326 | 271484 |
| P34955 | 28225400 | 5511320 | 12820900 | 6110660 | 8410430 | 8622390 | 12149300 | 6927570 |
| P37980 | 750144 | 2099680 | 2611710 | 2735680 | 2008470 | 2969680 | 8074520 | 3731830 |
| P46193 | 3497170 | 5195460 | 29975500 | 7707910 | 17112200 | 30812800 | 27853400 | 11571100 |
| P48644 | 1881800 | 4532590 | 4487570 | 5741520 | 2459280 | 1168260 | 15779300 | 2164610 |
| P50227 | 335089 | 657693 | 1181630 | 922620 | 900139 | 1351040 | 1627460 | 1208770 |
| P50397 | 18721500 | 42888200 | 49915100 | 54908800 | 59956700 | 69360500 | 80644000 | 54475900 |
| P52175 | 207170 | 471349 | 1258090 | 435140 | 863731 | 652200 | 457606 | 320828 |
| P53712 | 420162 | 755771 | 1272680 | 2108290 | 1180720 | 2081550 | 1002890 | 1405830 |
| P55859 | 706222 | 1384520 | 1460590 | 1235630 | 741800 | 1390550 | 9919560 | 3846110 |
| P60661 | 204309 | 693407 | 1464210 | 689887 | 816229 | 805409 | 1270910 | 1079910 |
| P61085 | 44986 | 229491 | 277518 | 141580 | 226556 | 322902 | 437301 | 337014 |
| P61157 | 1577580 | 3389970 | 5451180 | 2821300 | 3372740 | 7089090 | 5654140 | 2573180 |
| P61223 | 12923500 | 24921900 | 31333000 | 55891400 | 28632800 | 47261200 | 51851500 | 61192700 |
| P61284 | 94623.7 | 110887 | 209326 | 246675 | 235467 | 376260 | 455777 | 136617 |
| P61420 | 355092 | 608702 | 977611 | 1316620 | 1011590 | 1308130 | 839170 | 971202 |
| P61585 | 2939720 | 7328750 | 9299240 | 8708780 | 6789730 | 10417000 | 7882150 | 11741400 |
| P61625 | 11515600 | 1425470 | 3465550 | 1862160 | 4328420 | 3028400 | 4904070 | 4531250 |
| P62157 | 1164730 | 2993680 | 5833530 | 3473240 | 4170480 | 3718220 | 3478100 | 4252440 |
| P62248 | 658815 | 195527 | 545354 | 101802 | 169018 | 105171 | 372816 | 384388 |
| P62833 | 980312 | 2240450 | 3288920 | 3527790 | 1840250 | 2580340 | 3359060 | 2345140 |
| P62871 | 9071340 | 13851200 | 20590400 | 26204700 | 14835900 | 23976600 | 41778800 | 34351100 |
| P62935 | 8189810 | 19029000 | 24198300 | 31521100 | 17788200 | 19695500 | 40976100 | 28266400 |
| P63009 | 270446 | 670449 | 913494 | 846038 | 594580 | 1054850 | 1544430 | 379060 |
| P63103 | 9975790 | 20624500 | 39573000 | 28304100 | 33247300 | 41684000 | 46860700 | 32643000 |
| P67774 | 50363.2 | 171763 | 378716 | 219427 | 194230 | 343634 | 497080 | 373872 |
| P68103 | 7529650 | 18290100 | 22647900 | 23243900 | 17659000 | 26172900 | 42911800 | 24901300 |
| P68138 | 3479340 | 11663800 | 12312600 | 4875430 | 5295610 | 9142350 | 8878870 | 6395310 |
| P68252 | 2399880 | 5215660 | 7748510 | 5066140 | 6637990 | 11295300 | 9309960 | 3912400 |
| P68265 | 513588 | 574350 | 999208 | 722293 | 1061860 | 1332290 | 897832 | 814774 |
| P68401 | 0 | 128965 | 245904 | 201666 | 135636 | 230643 | 313655 | 145001 |
| P68509 | 1440190 | 2845970 | 3217210 | 2755010 | 3255990 | 4600960 | 5656950 | 2120080 |
| P79121 | 2705720 | 1889750 | 4132490 | 1582620 | 1905750 | 1089310 | 2667900 | 3309840 |
| P80195 | 9076740 | 3689800 | 7694500 | 30853600 | 3021620 | 3130550 | 20725600 | 12308100 |
| P80311 | 999066 | 314525 | 741022 | 367123 | 373687 | 483342 | 1357520 | 1107010 |
| P80457 | 686790000 | 1724520000 | 1428030000 | 1420830000 | 1646560000 | 2065610000 | 4076250000 | 2102300000 |
| P81019 | 57465.2 | 109901 | 153232 | 96146.3 | 0 | 97507.7 | 102579 | 156252 |
| P81125 | 2186950 | 4504790 | 7035800 | 4492680 | 6682420 | 9125470 | 8931340 | 8125150 |
| P81134 | 23973500 | 5133840 | 14691100 | 5188800 | 9824250 | 7117200 | 12918500 | 10467300 |
| P81265 | 584281000 | 612673000 | 378347000 | 598000000 | 273976000 | 563252000 | 313235000 | 326626000 |
| P81287 | 37536400 | 62696100 | 272922000 | 189910000 | 157376000 | 181112000 | 133537000 | 124329000 |
| P81947 | 3116270 | 4331610 | 10106400 | 4421690 | 4454550 | 7307940 | 8739000 | 6394680 |
| P84466 | 309805 | 580570 | 472742 | 916856 | 382744 | 576103 | 2512600 | 1362250 |
| Q08DI5 | 1006650 | 2195750 | 3032300 | 2561200 | 3064820 | 4159900 | 2465460 | 2001660 |
| Q08DK7 | 1393770 | 410342 | 777855 | 123338 | 831862 | 316942 | 766001 | 356009 |
| Q08DN8 | 2258880 | 3827890 | 4939260 | 11290800 | 4628200 | 7357560 | 12096000 | 9400690 |
| Q08DR7 | 344085 | 822929 | 700661 | 3717190 | 959005 | 1054450 | 903364 | 1239050 |
| Q08DV2 | 0 | 178457 | 211570 | 152370 | 195521 | 266656 | 452399 | 198490 |
| Q08DW9 | 1490550 | 214894 | 833528 | 302991 | 550141 | 292346 | 622703 | 550992 |
| Q08E11 | 1232450 | 388297 | 904896 | 451953 | 582162 | 953342 | 1695900 | 1508520 |
| Q08E32 | 2566090 | 5701920 | 6458810 | 19193500 | 11807400 | 9686330 | 7751860 | 4717250 |
| Q0II59 | 3886820 | 10498200 | 16288700 | 10102500 | 9667240 | 11819600 | 15345100 | 12656800 |
| Q0IIG7 | 1307440 | 2588220 | 3084920 | 5846160 | 3316400 | 3974150 | 4511920 | 3997960 |
| Q0IIG8 | 19640400 | 43401600 | 47854500 | 58618300 | 45584100 | 56273200 | 62215300 | 65124500 |
| Q0P5B0 | 5870100 | 7406020 | 9617610 | 23683800 | 10565000 | 11446400 | 7923800 | 17543300 |
| Q0P5F3 | 489673 | 1631430 | 3366060 | 3585650 | 1405830 | 2779810 | 5131890 | 1886710 |
| Q0V8R6 | 1902640 | 289420 | 1018870 | 313489 | 658711 | 353820 | 755110 | 723056 |
| Q0V8S0 | 49018.8 | 236183 | 481887 | 753094 | 369494 | 359850 | 541276 | 175859 |
| Q0VC36 | 358548 | 1047860 | 2348720 | 1717310 | 1472260 | 1860900 | 4980090 | 1674230 |
| Q0VCB5 | 3378530 | 764740 | 2907990 | 432852 | 1583210 | 1758690 | 1470630 | 1988120 |
| Q0VCH9 | 70107.9 | 516180 | 605751 | 2471490 | 452945 | 999096 | 4920950 | 1607890 |
| Q0VCJ8 | 1642400 | 511236 | 935071 | 807083 | 652572 | 793517 | 1456370 | 1257330 |
| Q0VCK1 | 309001 | 1297220 | 2169200 | 1457360 | 1532120 | 2647200 | 1288580 | 2254890 |
| Q0VCM0 | 68621.2 | 110609 | 529474 | 221657 | 346878 | 242418 | 63148.3 | 96719.2 |
| Q0VCM5 | 4910170 | 760475 | 2179350 | 729798 | 924505 | 783374 | 1011370 | 1182720 |
| Q0VCN9 | 38656000 | 39631700 | 74665200 | 111363000 | 43296300 | 82770800 | 48174400 | 45416600 |
| Q0VCP3 | 1026630 | 78134.1 | 341852 | 221995 | 308818 | 270893 | 561466 | 408929 |
| Q0VCQ6 | 1667650 | 5154600 | 5918660 | 5754170 | 6241970 | 7823940 | 11954800 | 6084320 |
| Q0VCX2 | 16918300 | 3549580 | 9247140 | 3701220 | 8060990 | 4747600 | 11675000 | 6075860 |
| Q0VCX5 | 0 | 274744 | 302839 | 220231 | 219904 | 312912 | 502143 | 264962 |
| Q0VD48 | 7804260 | 18939500 | 13328900 | 60296800 | 30710100 | 32037900 | 29434800 | 18583500 |
| Q0VD53 | 544292 | 638838 | 614412 | 524319 | 710237 | 864941 | 1506790 | 523826 |
| Q0VFX8 | 0 | 0 | 88209.6 | 0 | 51144.7 | 86553 | 579872 | 145937 |
| Q148C9 | 532640 | 776443 | 977579 | 1128050 | 774510 | 1546370 | 2418410 | 1625730 |
| Q148J6 | 1887370 | 3968790 | 7286600 | 3399000 | 3774790 | 8399700 | 6493240 | 3616780 |
| Q17QE5 | 500849 | 1959890 | 1831260 | 9698600 | 958702 | 1096100 | 3661040 | 2578260 |
| Q17QH1 | 499504 | 598414 | 519863 | 1159970 | 591348 | 499806 | 748649 | 691787 |
| Q17QT7 | 1098990 | 3647640 | 2033810 | 8553880 | 8028750 | 4878550 | 5819160 | 3092010 |
| Q17R06 | 761462 | 1169300 | 2586300 | 2507870 | 2000920 | 2459690 | 3403710 | 2281200 |
| Q1JP79 | 190593 | 349135 | 554141 | 352964 | 341836 | 910567 | 786703 | 205105 |
| Q1JPB0 | 3712680 | 5913600 | 13938600 | 5498870 | 7633490 | 12563100 | 15186900 | 5135890 |
| Q1JPD9 | 24581700 | 61534700 | 43041900 | 295614000 | 40379200 | 46446000 | 38946400 | 150730000 |
| Q1JQD4 | 206095 | 759776 | 713134 | 1561440 | 888299 | 1650900 | 3311010 | 2524540 |
| Q1RMI2 | 1239370 | 10309300 | 4200140 | 6165930 | 18146300 | 11577400 | 5533910 | 5937490 |
| Q1RMR9 | 778759 | 757593 | 2460760 | 2473320 | 683637 | 3641740 | 2896220 | 1737790 |
| Q1RMW9 | 141185 | 204232 | 273458 | 253172 | 308518 | 363245 | 573749 | 176002 |
| Q1RMX7 | 176718 | 408921 | 880140 | 433847 | 376029 | 631732 | 1209760 | 383880 |
| Q27975 | 11251300 | 16467400 | 14721500 | 82310100 | 27641000 | 26026300 | 27227700 | 22641900 |
| Q28024 | 299917 | 855623 | 1064510 | 805807 | 588783 | 813942 | 761837 | 1451310 |
| Q28046 | 659416 | 54605.6 | 793141 | 0 | 365941 | 993753 | 985640 | 540673 |
| Q28065 | 495938 | 29212.6 | 13777.8 | 211896 | 182267 | 43019 | 336220 | 39063.5 |
| Q28125 | 78486.5 | 710174 | 758323 | 1918950 | 818227 | 885180 | 1822580 | 1894330 |
| Q29455 | 242159 | 344554 | 595101 | 1282040 | 656348 | 905130 | 1607410 | 1138670 |
| Q29460 | 201468 | 598623 | 672748 | 556824 | 583257 | 1074610 | 1503730 | 862667 |
| Q29RQ1 | 6700190 | 1221960 | 2831110 | 747768 | 1450010 | 1210720 | 1908660 | 3804830 |
| Q29RR7 | 0 | 65885.7 | 96293.9 | 816233 | 173023 | 164554 | 130441 | 189154 |
| Q29RU0 | 430283 | 571643 | 1068680 | 1505700 | 599940 | 467399 | 887326 | 741488 |
| Q29RY7 | 3470970 | 1361590 | 769803 | 1509600 | 160473 | 2450540 | 822149 | 2492720 |
| Q29S21 | 337035 | 517807 | 1059080 | 1027940 | 640392 | 917104 | 1066890 | 711085 |
| Q2HJG5 | 0 | 133320 | 252638 | 258645 | 25046.5 | 242713 | 530950 | 184835 |
| Q2KI48 | 1231250 | 1898200 | 5107540 | 7733780 | 5369130 | 2483030 | 4371830 | 1790410 |
| Q2KIG3 | 317332 | 47704.3 | 78519.8 | 45019.3 | 69852.2 | 41424.4 | 64274.4 | 137422 |
| Q2KIP5 | 211472 | 655672 | 1196570 | 896494 | 1092360 | 661552 | 670114 | 1228650 |
| Q2KIS4 | 5350870 | 7852980 | 7841640 | 9503980 | 8240860 | 13547200 | 18182500 | 13045800 |
| Q2KIT0 | 20390600 | 1467510 | 2154830 | 382633 | 3234890 | 1441390 | 1895340 | 464227 |
| Q2KIV8 | 889588 | 2142760 | 3778100 | 2017070 | 3848900 | 5704600 | 5836080 | 2998510 |
| Q2KIV9 | 4027110 | 4556080 | 5146240 | 5528210 | 2166100 | 3207530 | 5530730 | 4845730 |
| Q2KIW4 | 2432850 | 366356 | 1513610 | 497702 | 2265450 | 1200070 | 1018400 | 960939 |
| Q2KIX7 | 6698180 | 880166 | 984049 | 256934 | 1569050 | 549690 | 1112520 | 206110 |
| Q2KJ20 | 2949720 | 560005 | 1703250 | 560511 | 792930 | 390554 | 780141 | 254722 |
| Q2KJ41 | 1534230 | 2599220 | 5785690 | 10317000 | 4376120 | 3822750 | 1979030 | 2971250 |
| Q2KJ54 | 432317 | 1706370 | 1238850 | 2603280 | 1166770 | 1767310 | 12205400 | 432038 |
| Q2KJD0 | 2141210 | 4313880 | 9149500 | 4204590 | 4695790 | 4502990 | 11058400 | 5863680 |
| Q2KJE7 | 464334 | 1724780 | 2861650 | 2543760 | 1606780 | 3745210 | 3936040 | 1561410 |
| Q2KJH9 | 1147620 | 1388810 | 4339310 | 2044650 | 3001950 | 2045710 | 2913410 | 1741430 |
| Q2KJJ8 | 802230 | 416728 | 1033470 | 1934960 | 770838 | 379760 | 685448 | 944785 |
| Q2LGB5 | 1524380 | 1937900 | 2944260 | 7474630 | 1699190 | 2944880 | 4602880 | 3292300 |
| Q2T9M8 | 12170300 | 11417200 | 19347800 | 21828200 | 15994200 | 18803800 | 19305200 | 13214200 |
| Q2T9X2 | 43493.6 | 370855 | 281736 | 123812 | 193289 | 402282 | 463068 | 135886 |
| Q2TA08 | 0 | 217121 | 85261.4 | 376322 | 415618 | 252893 | 248571 | 375047 |
| Q2TA46 | 226879 | 300649 | 530804 | 1023070 | 397498 | 311676 | 500312 | 913502 |
| Q2TBH2 | 1013710 | 2773530 | 4388700 | 4247670 | 3136900 | 4484330 | 5037850 | 3254290 |
| Q2TBU0 | 4924020 | 4150720 | 14692600 | 4590350 | 2681300 | 7727580 | 35314000 | 3644630 |
| Q2TBX4 | 2601940 | 377709 | 904436 | 246611 | 956032 | 806539 | 879897 | 1261900 |
| Q2UVX4 | 839107000 | 353134000 | 390073000 | 279640000 | 300837000 | 321905000 | 310154000 | 295103000 |
| Q2YDJ4 | 174547 | 266390 | 450925 | 531664 | 413014 | 501195 | 1263390 | 905758 |
| Q2YDJ9 | 265987 | 1240510 | 1744690 | 1766870 | 1270900 | 1902210 | 3766910 | 1860080 |
| Q32KR9 | 71652.8 | 240955 | 197250 | 1017010 | 288826 | 340168 | 263739 | 60713.1 |
| Q32KV6 | 17118300 | 4066520 | 12329000 | 5593230 | 6694940 | 5255840 | 13588300 | 11812600 |
| Q32L48 | 1361280 | 831146 | 3094660 | 695846 | 1477420 | 1240470 | 3199840 | 3188730 |
| Q32L99 | 448416 | 1213090 | 1755110 | 949639 | 1343790 | 1227060 | 1657550 | 2002970 |
| Q32LJ4 | 1910040 | 744987 | 1359650 | 830603 | 850610 | 747066 | 1394820 | 1181000 |
| Q32LK2 | 402345 | 1146950 | 1169910 | 3171430 | 1401860 | 972460 | 1028060 | 82138.5 |
| Q32PA1 | 16808400 | 23046700 | 36053900 | 56306900 | 27570800 | 33514100 | 34703500 | 36250500 |
| Q32PI5 | 30902.5 | 229553 | 204478 | 228084 | 149713 | 242479 | 488762 | 100489 |
| Q32PJ2 | 137008000 | 51088500 | 136174000 | 44016400 | 80058700 | 71121800 | 136582000 | 142418000 |
| Q3B7M5 | 44558.5 | 525955 | 799983 | 775085 | 420539 | 1668840 | 1390920 | 442017 |
| Q3MHE8 | 2438410 | 370200 | 912777 | 359299 | 459599 | 217216 | 816016 | 525178 |
| Q3MHL4 | 1297420 | 2672180 | 4342340 | 2312390 | 3544760 | 4135010 | 5168220 | 4724320 |
| Q3MHL7 | 0 | 430228 | 486909 | 355626 | 295917 | 513201 | 1040010 | 382995 |
| Q3MHM5 | 526136 | 737116 | 1795120 | 788901 | 828580 | 1062380 | 1597130 | 197325 |
| Q3MHN2 | 21224700 | 2807990 | 8098230 | 4547500 | 4260060 | 3371960 | 3978980 | 9231640 |
| Q3MHP2 | 8963790 | 20085000 | 38884200 | 39048800 | 36999600 | 36897700 | 60994200 | 35060700 |
| Q3MHR3 | 483699 | 650219 | 887185 | 962949 | 866772 | 798391 | 1186320 | 656955 |
| Q3MHR7 | 1395800 | 2644950 | 3704070 | 2294380 | 2436570 | 5996910 | 4446410 | 2476510 |
| Q3MHW6 | 5008920 | 17253500 | 28219100 | 56998600 | 51925300 | 32836400 | 25024000 | 12178800 |
| Q3MI05 | 452849 | 57528.5 | 473624 | 0 | 264029 | 101503 | 320735 | 78272.3 |
| Q3SWW9 | 0 | 80708.2 | 187397 | 242590 | 59858.2 | 243412 | 392036 | 191719 |
| Q3SX14 | 12913500 | 3485180 | 8638090 | 3132530 | 4096110 | 4529310 | 10483000 | 9774660 |
| Q3SX42 | 282371 | 959734 | 1087230 | 474757 | 2442490 | 2248280 | 280575 | 0 |
| Q3SX46 | 8257460 | 1374280 | 4826960 | 1718420 | 1853610 | 1719950 | 5437170 | 4186620 |
| Q3SYS6 | 121566 | 571321 | 697910 | 942573 | 508721 | 823378 | 1441960 | 1975880 |
| Q3SYU2 | 2072940 | 4582840 | 5561330 | 6216850 | 4479340 | 6317540 | 14314400 | 8467230 |
| Q3SYX9 | 294964 | 687810 | 1063520 | 873446 | 761685 | 1146120 | 955453 | 704495 |
| Q3SYZ4 | 27717.8 | 35210.1 | 100627 | 78633.4 | 274405 | 359654 | 360084 | 124368 |
| Q3SZ54 | 878236 | 2440500 | 3914850 | 2862640 | 3320370 | 4526040 | 5212360 | 3250080 |
| Q3SZ62 | 1305310 | 1523470 | 4861390 | 1795150 | 2781080 | 3593850 | 4043010 | 2270930 |
| Q3SZA1 | 189725 | 1337200 | 918979 | 4072640 | 1620290 | 2036520 | 2544980 | 1278310 |
| Q3SZB7 | 243807 | 269320 | 492567 | 349703 | 64773.8 | 372030 | 566286 | 121220 |
| Q3SZF2 | 978591 | 2880560 | 3793070 | 2910210 | 4295990 | 4404210 | 5319640 | 1574990 |
| Q3SZH7 | 1857630 | 2175830 | 4244500 | 2253650 | 3201940 | 4388470 | 13080500 | 2046530 |
| Q3SZI4 | 5439060 | 10952200 | 17551300 | 18161600 | 14927600 | 21391500 | 21534100 | 9590700 |
| Q3SZK8 | 4023630 | 8933210 | 19738100 | 18801200 | 9339620 | 13654300 | 40884400 | 22550400 |
| Q3SZV7 | 28525000 | 11963900 | 12094200 | 5728690 | 11169600 | 13526600 | 5892620 | 4357840 |
| Q3T035 | 184524 | 799378 | 1438620 | 120463 | 212930 | 2163370 | 172780 | 183309 |
| Q3T054 | 466259 | 1219850 | 1489230 | 1674310 | 1200880 | 2023240 | 4287340 | 1204790 |
| Q3T067 | 373773 | 760657 | 916311 | 1733670 | 1181100 | 1814220 | 9498590 | 1622580 |
| Q3T0D5 | 95731.5 | 186197 | 350810 | 65448.7 | 204442 | 254568 | 224996 | 36012.3 |
| Q3T0D7 | 1734320 | 2806470 | 2821500 | 3163380 | 2414650 | 3869090 | 5194080 | 5427230 |
| Q3T0L2 | 1001550 | 50112.9 | 94817.8 | 0 | 130252 | 77770.7 | 282949 | 103964 |
| Q3T0L5 | 1423400 | 5735840 | 6208750 | 8648630 | 6665700 | 13818100 | 2875200 | 3588110 |
| Q3T0N1 | 2757570 | 5188880 | 4219240 | 24821800 | 5087250 | 6554980 | 5994940 | 13367400 |
| Q3T0Q2 | 830119 | 656970 | 1901820 | 3616560 | 2671760 | 781235 | 2348840 | 1563170 |
| Q3T0Q4 | 6080700 | 13188900 | 27240900 | 29293100 | 17812100 | 14054000 | 28666400 | 23396700 |
| Q3T0R1 | 77822.4 | 211748 | 351940 | 8276.96 | 847274 | 465223 | 273084 | 98361.3 |
| Q3T0T1 | 97964.4 | 285946 | 658745 | 296310 | 441592 | 384393 | 602746 | 343968 |
| Q3T0U5 | 2534210 | 11352300 | 9206650 | 2915840 | 22357000 | 19516900 | 543060 | 246129 |
| Q3T0Y8 | 528721 | 1823150 | 5080410 | 3039270 | 3245890 | 1857770 | 2646930 | 2835910 |
| Q3T105 | 0 | 196789 | 185955 | 256119 | 112879 | 293040 | 532582 | 350078 |
| Q3T114 | 201627 | 442482 | 970465 | 1291180 | 825757 | 363834 | 888515 | 975249 |
| Q3T145 | 1086180 | 2298920 | 5060200 | 5358220 | 2935750 | 4410560 | 6687670 | 3543170 |
| Q3T149 | 3553460 | 6732840 | 11135400 | 7788640 | 7103060 | 13789600 | 13508400 | 7061230 |
| Q3T178 | 2548400 | 8027040 | 6499990 | 39341400 | 8746530 | 7570250 | 5614710 | 9765600 |
| Q3ZBD7 | 1451430 | 1987960 | 3809400 | 1932700 | 2820120 | 3758830 | 3693400 | 2233780 |
| Q3ZBG0 | 488863 | 853666 | 1546580 | 1446200 | 1103370 | 1707860 | 2596390 | 787835 |
| Q3ZBG9 | 211504 | 2124570 | 724790 | 9780400 | 1799340 | 986337 | 2167260 | 3626300 |
| Q3ZBL4 | 71518.2 | 89096.6 | 201902 | 216365 | 134540 | 155257 | 329424 | 150723 |
| Q3ZBS7 | 18072400 | 6629720 | 16223300 | 9904470 | 11615200 | 7405320 | 10086500 | 9019870 |
| Q3ZBT1 | 952193 | 1198020 | 1573930 | 1934000 | 1205030 | 1334520 | 2037340 | 1346510 |
| Q3ZBT5 | 118346 | 191116 | 1222260 | 1201320 | 815628 | 757655 | 885209 | 339592 |
| Q3ZBV1 | 11516600 | 37193800 | 36613000 | 84912300 | 79101800 | 82483100 | 23279900 | 23031300 |
| Q3ZBV6 | 86378.5 | 250926 | 614151 | 894385 | 473286 | 96811 | 0 | 595079 |
| Q3ZBV8 | 900656 | 1543610 | 2790440 | 1826950 | 1575740 | 2146150 | 4292380 | 2143030 |
| Q3ZBW4 | 33266.6 | 168040 | 330573 | 109201 | 330735 | 192581 | 422589 | 270660 |
| Q3ZBW5 | 0 | 130905 | 161343 | 382492 | 118934 | 155335 | 167974 | 156514 |
| Q3ZBZ8 | 326160 | 876850 | 1193060 | 2187430 | 1217740 | 1935810 | 2224190 | 3553690 |
| Q3ZC13 | 40566.3 | 119634 | 215618 | 0 | 226441 | 439206 | 692460 | 308743 |
| Q3ZC35 | 485476 | 147932 | 332048 | 38259.4 | 341976 | 130729 | 633034 | 167243 |
| Q3ZC42 | 196073 | 600837 | 638184 | 395592 | 568593 | 935155 | 1026200 | 583675 |
| Q3ZCD0 | 35762700 | 67051100 | 46000500 | 378872000 | 58791000 | 60319800 | 40423100 | 156117000 |
| Q3ZCI9 | 76991.1 | 340773 | 354113 | 435701 | 241922 | 376802 | 867020 | 372572 |
| Q3ZCJ2 | 1787690 | 2742840 | 5555610 | 2876950 | 4565460 | 4211240 | 5764710 | 6015010 |
| Q3ZCK2 | 4035140 | 3548680 | 5609380 | 11720400 | 3873870 | 6229380 | 10497600 | 6226160 |
| Q3ZCK3 | 447826 | 1161710 | 1932710 | 2218050 | 1540490 | 1584670 | 2685690 | 1811250 |
| Q3ZCK9 | 116716 | 412888 | 637784 | 250723 | 561781 | 670906 | 593782 | 237283 |
| Q3ZCL0 | 262908 | 551196 | 0 | 0 | 0 | 743441 | 177905 | 168822 |
| Q4GZT4 | 46919900 | 112063000 | 96948600 | 158947000 | 140345000 | 167208000 | 288300000 | 218980000 |
| Q56JV6 | 187070 | 711283 | 1329730 | 989981 | 722574 | 1548540 | 1671480 | 943650 |
| Q56JW4 | 1546760 | 3142550 | 5128940 | 3744240 | 3600120 | 5025730 | 6300070 | 5296580 |
| Q58CQ2 | 896041 | 1745130 | 2414840 | 1703400 | 1690760 | 4215760 | 3820520 | 1858650 |
| Q58CS7 | 572893 | 1749930 | 1408390 | 4999040 | 4211000 | 3520250 | 2948780 | 1203150 |
| Q58CS8 | 1114880 | 255919 | 678120 | 223982 | 570517 | 309850 | 543093 | 266054 |
| Q58CW5 | 528955 | 1162380 | 532351 | 5584590 | 358336 | 1138770 | 1749650 | 988901 |
| Q58CY6 | 406737 | 1614630 | 1491550 | 319804 | 2454130 | 3697880 | 972210 | 490858 |
| Q58D08 | 817054 | 498628 | 2041230 | 1432510 | 692881 | 2857490 | 2496650 | 1208140 |
| Q58DA7 | 0 | 150086 | 159679 | 87926.6 | 153414 | 170249 | 303015 | 155417 |
| Q58DC0 | 1090940 | 2247940 | 3903700 | 4139620 | 3378320 | 4377520 | 5136060 | 3377030 |
| Q58DD3 | 179581 | 145501 | 536482 | 559204 | 431511 | 313364 | 445399 | 262305 |
| Q58DG6 | 267149 | 768981 | 729785 | 1999700 | 703026 | 1012500 | 1731010 | 1299940 |
| Q58DK5 | 145660 | 273774 | 648477 | 541052 | 455210 | 616556 | 526095 | 403352 |
| Q58DS5 | 345867 | 759559 | 1009210 | 1555400 | 687526 | 1332890 | 2342910 | 1849170 |
| Q58DU5 | 262185 | 633538 | 823720 | 377693 | 744037 | 1005390 | 1139490 | 227987 |
| Q58DW6 | 1619720 | 3156070 | 5321040 | 8410750 | 3945800 | 6336730 | 8604150 | 5760380 |
| Q5BIP4 | 345302 | 941083 | 1819430 | 2477030 | 1953760 | 1859850 | 2117260 | 1331300 |
| Q5E946 | 2065980 | 3732960 | 7705020 | 7392480 | 5391150 | 8391340 | 11260600 | 5251880 |
| Q5E947 | 2104610 | 5878050 | 8798060 | 7917420 | 7771670 | 9631060 | 12325300 | 5875450 |
| Q5E954 | 2216550 | 2994160 | 2726620 | 7590020 | 4743790 | 5230360 | 5097050 | 4547460 |
| Q5E956 | 1738140 | 2873710 | 5884240 | 3981970 | 3912940 | 5995040 | 7979400 | 3963020 |
| Q5E958 | 46592.1 | 340322 | 235328 | 69604.2 | 238713 | 555616 | 761672 | 0 |
| Q5E963 | 313876 | 464664 | 818706 | 657126 | 420068 | 669118 | 611072 | 286341 |
| Q5E994 | 2217280 | 6466110 | 5059670 | 2099510 | 9254210 | 10719700 | 8015230 | 0 |
| Q5E997 | 2311410 | 4483870 | 5731060 | 6260170 | 5547510 | 8908270 | 6277330 | 5795020 |
| Q5E9A6 | 724066 | 1730810 | 2215680 | 7229060 | 1724870 | 2346250 | 1649310 | 2147260 |
| Q5E9D5 | 583144 | 1074710 | 2247630 | 1541550 | 1017030 | 1734910 | 2660930 | 1115440 |
| Q5E9E2 | 95058 | 124596 | 714433 | 279997 | 145145 | 178171 | 221501 | 161026 |
| Q5E9E3 | 4807350 | 7207970 | 6762690 | 4146690 | 3351860 | 5047370 | 3557550 | 3826790 |
| Q5E9F7 | 9267960 | 18291800 | 35629600 | 21425600 | 18455800 | 36103800 | 44749700 | 21925900 |
| Q5E9G3 | 237144 | 1203130 | 1663140 | 1382830 | 1154770 | 1929310 | 2416820 | 1309530 |
| Q5E9I4 | 22128700 | 8723470 | 16031900 | 10059600 | 13174900 | 6088110 | 18449700 | 26156800 |
| Q5E9K0 | 218811 | 412613 | 716125 | 497970 | 488330 | 721233 | 856556 | 404350 |
| Q5E9T8 | 0 | 18598.5 | 0 | 52183 | 0 | 0 | 236329 | 55565 |
| Q5EA01 | 4016960 | 990155 | 2292700 | 1281710 | 1322000 | 1042060 | 1962050 | 1990970 |
| Q5EA79 | 5049460 | 12414300 | 16310700 | 18764300 | 11708400 | 16480800 | 18751200 | 12890400 |
| Q5EA88 | 107033 | 212799 | 215137 | 624305 | 184564 | 378000 | 1233810 | 593598 |
| Q5EAB4 | 11562400 | 2621360 | 6367600 | 1911650 | 5655070 | 3454060 | 6590810 | 5475540 |
| Q5EAC2 | 241584 | 516461 | 1125730 | 1148560 | 823993 | 1093260 | 1512880 | 811527 |
| Q5EAD0 | 3337830 | 4570290 | 6456010 | 9859430 | 6241780 | 7165400 | 7582070 | 3899700 |
| Q5EAD2 | 55576.2 | 503717 | 614290 | 328046 | 323826 | 704685 | 1357540 | 809850 |
| Q5GN72 | 362264 | 39537.9 | 175595 | 481726 | 132922 | 145654 | 55275 | 35673.4 |
| Q6EWQ7 | 70389.8 | 500182 | 616974 | 793725 | 564324 | 844597 | 1373940 | 732161 |
| Q6QRN6 | 454590 | 196291 | 450607 | 403732 | 302693 | 311135 | 576523 | 275046 |
| Q6QRN7 | 6553920 | 22031000 | 16525200 | 61385500 | 21647600 | 31071700 | 32842900 | 19091400 |
| Q6R8F2 | 2475110 | 1270520 | 2060530 | 1175070 | 1244620 | 680413 | 1430090 | 947922 |
| Q762I5 | 250634 | 2058150 | 4998300 | 428577 | 383879 | 1485620 | 5685810 | 451704 |
| Q765N9 | 2236910 | 7380570 | 10787200 | 5703650 | 12564600 | 14684500 | 2317340 | 3806010 |
| Q76LV2 | 4527170 | 12243600 | 18947300 | 15424800 | 10632300 | 17537700 | 24113800 | 13273200 |
| Q8SPP7 | 12927400 | 10320700 | 10733600 | 4510610 | 5972010 | 9283320 | 12726900 | 4830820 |
| Q8SQ28 | 6837460 | 6408770 | 17698000 | 7143360 | 4775430 | 4175720 | 7709320 | 10903400 |
| Q92176 | 35914.3 | 0 | 319793 | 52160.5 | 176223 | 270010 | 598059 | 143783 |
| Q95107 | 0 | 120999 | 117059 | 623893 | 204239 | 187014 | 339729 | 250963 |
| Q95122 | 7450020 | 29856000 | 22231200 | 142737000 | 22819000 | 24837700 | 26165100 | 83395000 |
| Q95135 | 974610 | 2581840 | 5216420 | 4419930 | 2082750 | 3730140 | 2505950 | 2805790 |
| Q95JC7 | 566160 | 3097010 | 3142970 | 11876800 | 1897740 | 2230270 | 1411770 | 3510200 |
| Q95M18 | 1855700 | 458194 | 1263800 | 297597 | 960513 | 812881 | 2190720 | 1284620 |
| Q9BDR7 | 317696 | 340132 | 990624 | 1130110 | 604405 | 1003870 | 1716520 | 1428700 |
| Q9BGI2 | 21878300 | 6454240 | 14622300 | 10578000 | 8913910 | 13488400 | 14058900 | 9193360 |
| Q9BGI3 | 662606 | 1460700 | 3215840 | 1905010 | 1606610 | 2724380 | 3235130 | 1584820 |
| Q9GL65 | 2330620 | 5109250 | 5643700 | 12898700 | 4986080 | 5671170 | 6600950 | 6913250 |
| Q9GMB8 | 390201 | 1446450 | 1297840 | 1031610 | 894982 | 1157650 | 2275050 | 1151480 |
| Q9MZ06 | 531371 | 638616 | 387974 | 901884 | 546622 | 402696 | 670542 | 443327 |
| Q9MZ08 | 441082 | 314881 | 482756 | 974650 | 475050 | 331410 | 582012 | 667197 |
| Q9N2J2 | 80887.6 | 286650 | 463179 | 372225 | 294519 | 360778 | 925701 | 207728 |
| Q9TTE1 | 10621700 | 2381230 | 6582160 | 2205340 | 3348040 | 4616070 | 2027210 | 2871740 |
| Q9TU47 | 84070.8 | 90394.5 | 311518 | 510909 | 175703 | 281511 | 594284 | 235289 |
| Q9XSA7 | 5027960 | 10960000 | 19878800 | 8635510 | 8485170 | 13620300 | 37681000 | 19485000 |
| Q9XT56 | 558163 | 876555 | 1033270 | 2381780 | 965108 | 1854060 | 1402100 | 1253910 |

**Supplementary Table 2:** Differential protein abundance analysis. ^1^UniProtKB accession Identification. ^2^base expression mean across samples. NA = not assigned; FC = Fold Change; LR = Likelihood Ratio; FDR = False Discovery Rate

| **Protein_ID^1^** | **Gen symbol** | **Description** | **BaseMeanAbundance^2^** | **logFC** | **LR** | **p-value** | **FDR** |
| --- | --- | --- | --- | --- | --- | --- | --- |
| A0AAA9THP8 | *ANXA1* | annexin A1 | 7.37 | -26.52 | 1095.08 | 0.0000 | 4.20E-237 |
| F1N7C5 | *IDO1* | indoleamine 2,3-dioxygenase 1 | 5.66 | 2.58 | 32.12 | 0.0000 | 7.83E-06 |
| G3N0V0 | *Ig-like* | ig-like domain-containing protein | 9.82 | -2.92 | 20.79 | 0.0000 | 0.0018 |
| E1BDW9 | *EXOC2* | exocyst complex component 2 | -0.30 | 18.85 | 19.07 | 0.0000 | 0.0034 |
| A0A3Q1LJT1 | *Ig-like* | ig-like domain-containing protein | 4.40 | -3.08 | 17.61 | 0.0000 | 0.0059 |
| P12624 | *MARCKS* | myristoylated alanine rich protein kinase C substrate | 6.56 | 1.71 | 14.35 | 0.0002 | 0.0274 |
| F1MWI1 | *CLU* | clusterin | 3.45 | -2.70 | 13.75 | 0.0002 | 0.0323 |
| A0AAA9SGV1 | *NID1* | nidogen 1 | 2.94 | -4.60 | 13.40 | 0.0003 | 0.0340 |
| F1MYX5 | *LCP1* | lymphocyte cytosolic protein 1 | 3.39 | 2.30 | 12.61 | 0.0004 | 0.0392 |
| A5D7I4 | *EXT1* | exostosin glycosyltransferase 1 | 3.61 | -2.52 | 12.52 | 0.0004 | 0.0392 |
| A0AAA9TDK9 | *ARSA* | arylsulfatase A | 4.02 | -3.12 | 12.39 | 0.0004 | 0.0392 |
| A0A3Q1LWD3 | *DMBT1* | deleted in Malignant Brain Tumors 1 | 4.86 | -2.84 | 12.38 | 0.0004 | 0.0392 |
| E1BAW2 | *LRRC32* | leucine rich repeat containing 32 | 2.90 | -1.99 | 11.88 | 0.0006 | 0.0473 |
| A0AAF6ZEE7 | *CYB561* | NA | 3.21 | -1.39 | 11.49 | 0.0007 | 0.0541 |
| A0A3Q1MJT2 | *A1BG* | alpha-1-B glycoprotein | 7.50 | -5.84 | 10.44 | 0.0012 | 0.0850 |
| G5E5A8 | *FN1* | fibronectin 1 | 8.57 | -1.82 | 10.40 | 0.0013 | 0.0850 |
| A0AAA9TU07 | *GPX3* | glutathione peroxidase 3 | 6.29 | -1.74 | 8.53 | 0.0035 | 0.2225 |
| A0AAA9S0J0 | *HSD17B7* | hydroxysteroid 17-beta dehydrogenase 7 | 5.09 | 1.64 | 8.32 | 0.0039 | 0.2363 |
| A0AAA9S9Q3 | *PTPRC* | protein tyrosine phosphatase receptor type C | 2.20 | 2.11 | 7.80 | 0.0052 | 0.2799 |
| A0A3Q1MIL5 | *NA* | NA | 7.05 | -1.50 | 7.72 | 0.0055 | 0.2799 |
| G5E5V1 | *NA* | NA | 11.42 | -1.15 | 7.71 | 0.0055 | 0.2799 |
| P15396 | *ENPP3* | ectonucleotide pyrophosphatase/phosphodiesterase 3 | 5.74 | 2.17 | 7.59 | 0.0059 | 0.2799 |
| O18836 | *MSTN* | myostatin | 5.61 | -1.79 | 7.57 | 0.0059 | 0.2799 |
| A0A3Q1M3A8 | *NA* | NA | 4.44 | -1.74 | 7.48 | 0.0062 | 0.2812 |
| A5PK70 | *NME3* | NME/NM23 nucleoside diphosphate kinase 3 | 8.09 | -1.47 | 6.70 | 0.0096 | 0.4174 |
| A7E3W2 | *LGALS3BP* | NA | 6.47 | -2.84 | 6.53 | 0.0106 | 0.4283 |
| A6QL48 | *IL34* | interleukin 34 | 3.88 | -1.98 | 6.50 | 0.0108 | 0.4283 |
| E1B6Z6 | *LCN2* | lipocalin 2 | 8.70 | -2.05 | 6.39 | 0.0115 | 0.4283 |
| A5PKG9 | *FGR* | FGR proto-onco, Src family tyrosine kinase | 3.77 | 2.00 | 6.35 | 0.0118 | 0.4283 |
| A0AAA9RU48 | *ICAM1* | intercellular adhesion molecule 1 | 3.36 | 1.57 | 6.33 | 0.0119 | 0.4283 |
| F1MNV5 | *KNG1* | kininogen 1 | 2.31 | 1.45 | 6.22 | 0.0126 | 0.4358 |
| A0A3Q1LK49 | *ITIH2* | inter-alpha-trypsin inhibitor heavy chain 2 | 6.10 | -2.22 | 6.18 | 0.0129 | 0.4358 |
| A0AAA9RSR9 | *MAN1A2* | mannosidase alpha class 1A member 2 | 7.05 | -1.69 | 5.99 | 0.0144 | 0.4579 |
| A0AAA9TGA8 | *PDIA6* | protein disulfide isomerase family A member 6 | 2.39 | 1.30 | 5.97 | 0.0146 | 0.4579 |
| Q2KJ20 | *CHRDL2* | chordin like 2 | 5.19 | -2.07 | 5.94 | 0.0148 | 0.4579 |
| A0A452DHW7 | *CSN2* | casein beta | 12.98 | -2.34 | 5.82 | 0.0158 | 0.4688 |
| P08169 | *IGF2R* | NA | 3.97 | -1.52 | 5.80 | 0.0160 | 0.4688 |
| Q3SYZ4 | *DARS1* | NA | 2.13 | 1.99 | 5.65 | 0.0174 | 0.4925 |
| A0A3Q1LPI6 | *NA* | NA | 10.38 | -1.52 | 5.62 | 0.0178 | 0.4925 |
| A0A452DJD0 | *NA* | NA | 6.67 | -1.57 | 5.51 | 0.0189 | 0.4928 |
| Q3T067 | *SCCPDH* | NA | 5.72 | 1.50 | 5.49 | 0.0191 | 0.4928 |
| A6QPQ2 | *SERPINA3-8* | NA | 7.76 | -2.65 | 5.49 | 0.0191 | 0.4928 |
| A0AAA9SKJ1 | *A2M* | alpha-2-macroglobulin | 10.38 | -2.22 | 5.23 | 0.0221 | 0.5572 |
| Q0VCM5 | *ITIH1* | NA | 5.87 | -1.88 | 5.05 | 0.0247 | 0.6051 |
| A0AAA9SE43 | *GLG1* | golgi glycoprotein 1 | 4.93 | -3.61 | 4.99 | 0.0255 | 0.6051 |
| G5E5T5 | *NA* | NA | 7.47 | -1.38 | 4.92 | 0.0266 | 0.6051 |
| F1MIH9 | *PLBD2* | phospholipase B domain containing 2 | 4.28 | -1.97 | 4.89 | 0.0270 | 0.6051 |
| P32749 | *BCHE* | NA | 1.98 | -2.16 | 4.77 | 0.0289 | 0.6051 |
| A0A3Q1LXF3 | *CIDEA* | cell death inducing DFFA like effector a | 5.63 | 1.38 | 4.69 | 0.0303 | 0.6051 |
| A6QQN6 | *SMPDL3B* | sphingomyelin phosphodiesterase acid like 3B | 1.83 | 2.87 | 4.67 | 0.0306 | 0.6051 |
| A0A3Q1MZW0 | *FCGR2B* | Fc gamma receptor IIb | 7.55 | -0.95 | 4.61 | 0.0317 | 0.6051 |
| F1MJK3 | *LOC506828* | NA | 5.67 | -2.62 | 4.61 | 0.0318 | 0.6051 |
| A0AAA9TIR2 | *TCN2* | transcobalamin 2 | 5.33 | -1.74 | 4.60 | 0.0320 | 0.6051 |
| A0AAA9SEK8 | *SEMA7A* | semaphorin 7A | 3.92 | -2.57 | 4.59 | 0.0321 | 0.6051 |
| A0A3Q1LZ01 | *SPOCK1* | SPARC (osteonectin), cwcv and kazal like domains proteoglycan 1 | 7.18 | -1.13 | 4.59 | 0.0322 | 0.6051 |
| F1MHQ1 | *GPA33* | glycoprotein A33 | 4.80 | -1.02 | 4.58 | 0.0324 | 0.6051 |
| Q2KIT0 | *NA* | NA | 7.47 | -2.74 | 4.57 | 0.0325 | 0.6051 |
| Q6R8F2 | *CDH1* | NA | 5.53 | -1.26 | 4.53 | 0.0332 | 0.6051 |
| E1B805 | *NA* | NA | 6.08 | -2.77 | 4.53 | 0.0332 | 0.6051 |
| G3X6N3 | *TF* | transferrin | 8.04 | -1.42 | 4.52 | 0.0336 | 0.6051 |
| A0AAA9TQL1 | *DDR2* | discoidin domain receptor tyrosine kinase 2 | 4.09 | -1.28 | 4.40 | 0.0360 | 0.6232 |
| F1MJQ3 | *NA* | NA | 6.42 | -1.50 | 4.33 | 0.0374 | 0.6232 |
| A0AAA9U0R1 | *DPP10* | dipeptidyl peptidase like 10 | 5.55 | -1.00 | 4.31 | 0.0378 | 0.6232 |
| G3MYD9 | *ITGAM* | integrin subunit alpha M | 4.46 | 1.81 | 4.31 | 0.0379 | 0.6232 |
| F1N0I3 | *F5* | coagulation factor V | 6.42 | -1.52 | 4.29 | 0.0382 | 0.6232 |
| G5E513 | *NA* | NA | 17.35 | -1.15 | 4.28 | 0.0386 | 0.6232 |
| Q5E9E3 | *C1QA* | complement C1q A chain | 7.22 | -0.97 | 4.25 | 0.0393 | 0.6232 |
| F1MVJ8 | *OLFM4* | olfactomedin 4 | 7.01 | 1.89 | 4.24 | 0.0394 | 0.6232 |
| O46375 | *TTR* | transthyretin | 6.75 | -1.72 | 4.22 | 0.0400 | 0.6232 |
| F1N102 | *C8B* | complement C8 beta chain | 6.87 | -1.89 | 4.19 | 0.0406 | 0.6232 |
| Q3MHE8 | *SRPRA* | SRP receptor subunit alpha | 4.83 | -1.84 | 4.18 | 0.0409 | 0.6232 |
| Q1JQD4 | *GIPC2* | GIPC PDZ domain containing family member 2 | 5.20 | 1.12 | 4.11 | 0.0427 | 0.6264 |
| A0A3Q1LR40 | *LIPG* | lipase G, endothelial type | 6.12 | -1.46 | 4.10 | 0.0429 | 0.6264 |
| P02070 | *HBB* | hemoglobin, beta | 10.76 | -2.03 | 4.09 | 0.0430 | 0.6264 |
| A0A3Q1LQK2 | *IL1RAP* | interleukin 1 receptor accessory protein | 3.05 | -1.42 | 4.08 | 0.0434 | 0.6264 |
| P81265 | *PIGR* | NA | 13.82 | -1.05 | 4.03 | 0.0447 | 0.6364 |
| A0A3Q1LFK0 | *SLC1A2* | solute carrier family 1 member 2 | 7.91 | -1.23 | 4.01 | 0.0453 | 0.6368 |
| G3MZH7 | *ENTPD2* | ectonucleoside triphosphate diphosphohydrolase 2 | 6.32 | 0.93 | 3.90 | 0.0482 | 0.6639 |
| F1N5Z0 | *AQP5* | aquaporin 5 | 8.15 | -1.00 | 3.87 | 0.0492 | 0.6639 |
| P12763 | *AHSG* | NA | 3.35 | -1.77 | 3.83 | 0.0503 | 0.6639 |
| Q2KIX7 | *NA* | NA | 6.02 | -2.27 | 3.83 | 0.0504 | 0.6639 |
| Q2UVX4 | *C3* | complement C3 | 13.70 | -1.26 | 3.81 | 0.0510 | 0.6639 |
| F1N5T0 | *CUTA* | cutA divalent cation tolerance homolog | 3.34 | -1.57 | 3.80 | 0.0513 | 0.6639 |
| E1BBP4 | *DDR1* | discoidin domain receptor tyrosine kinase 1 | 3.43 | -1.92 | 3.79 | 0.0515 | 0.6639 |
| E1B726 | *PLG* | NA | 8.35 | -1.26 | 3.69 | 0.0548 | 0.6936 |
| A0AAF6ZE69 | *SHC1* | NA | 4.57 | -1.17 | 3.68 | 0.0551 | 0.6936 |
| F1MPP2 | *IGFBP7* | insulin like growth factor binding protein 7 | 4.18 | -1.50 | 3.63 | 0.0568 | 0.7062 |
| A0AAA9SXF2 | *MAN2A1* | mannosidase alpha class 2A member 1 | 6.57 | -1.22 | 3.58 | 0.0584 | 0.7179 |
| A0AAA9T5W3 | *MPZL1* | myelin protein zero like 1 | 5.03 | 1.20 | 3.45 | 0.0632 | 0.7244 |
| A0A452DJ03 | *VWA1* | von Willebrand factor A domain containing 1 | 7.33 | -1.58 | 3.43 | 0.0641 | 0.7244 |
| A0AAA9T1E6 | *JCHAIN* | joining chain of multimeric IgA and IgM | 12.08 | -0.91 | 3.41 | 0.0648 | 0.7244 |
| G3MWT2 | *NA* | NA | 7.66 | 1.37 | 3.39 | 0.0657 | 0.7244 |
| A6QNW7 | *CD5L* | CD5 molecule like | 11.33 | -1.04 | 3.37 | 0.0666 | 0.7244 |
| F1MX87 | *C8A* | complement C8 alpha chain | 4.04 | -2.08 | 3.36 | 0.0666 | 0.7244 |
| Q2KIV8 | *GSTM3* | glutathione S-transferase mu 3 | 6.50 | 0.79 | 3.36 | 0.0668 | 0.7244 |
| A0A3Q1LRP5 | *CFB* | complement factor B | 5.74 | -1.79 | 3.35 | 0.0672 | 0.7244 |
| F1MCX1 | *GPC4* | glypican 4 | 9.40 | -1.10 | 3.31 | 0.0688 | 0.7244 |
| G3X745 | *GPC1* | NA | 4.80 | -1.30 | 3.30 | 0.0693 | 0.7244 |
| Q5GN72 | *agp* | NA | 2.54 | -1.89 | 3.28 | 0.0702 | 0.7244 |
| F1MC11 | *KRT14* | keratin 14 | 8.09 | 0.93 | 3.27 | 0.0708 | 0.7244 |
| P15467 | *RNASE4* | ribonuclease A family member 4 | 3.03 | -1.81 | 3.26 | 0.0709 | 0.7244 |
| A0AAA9RTT5 | *GASK1A* | golgi associated kinase 1A | 7.72 | -1.28 | 3.26 | 0.0711 | 0.7244 |
| Q9TTE1 | *SERPINA3-1* | NA | 7.25 | -1.39 | 3.25 | 0.0713 | 0.7244 |
| E1BH06 | *NA* | NA | 8.07 | -1.36 | 3.25 | 0.0713 | 0.7244 |
| Q3MHN2 | *C9* | complement C9 | 8.04 | -1.54 | 3.25 | 0.0715 | 0.7244 |
| F1MPI4 | *POFUT2* | protein O-fucosyltransferase 2 | 5.66 | -1.45 | 3.24 | 0.0718 | 0.7244 |
| A6QR60 | *CRISPLD1* | cysteine rich secretory protein LCCL domain containing 1 | 7.96 | -1.21 | 3.23 | 0.0725 | 0.7244 |
| H7BWW0 | *CORO1B* | coronin 1B | 1.80 | 1.59 | 3.23 | 0.0725 | 0.7244 |
| A0AAA9SRZ8 | *SHBG* | sex hormone binding globulin | 4.39 | -1.38 | 3.21 | 0.0730 | 0.7244 |
| A0A452DHZ3 | *SERPINA5* | serpin family A member 5 | 6.21 | -1.83 | 3.18 | 0.0745 | 0.7249 |
| A0AAA9RY95 | *GBA1* | glucosylceramidase beta 1 | 4.84 | -1.25 | 3.18 | 0.0747 | 0.7249 |
| Q3SZV7 | *HPX* | NA | 8.71 | -1.41 | 3.17 | 0.0750 | 0.7249 |
| P55859 | *PNP* | NA | 5.99 | 1.20 | 3.13 | 0.0771 | 0.7282 |
| A0A3Q1LI44 | *NA* | NA | 8.00 | -1.33 | 3.13 | 0.0771 | 0.7282 |
| F1MNN7 | *LBP* | lipopolysaccharide binding protein | 8.25 | -1.32 | 3.11 | 0.0778 | 0.7282 |
| A0AAA9SHI3 | *NA* | NA | 4.03 | -1.43 | 3.10 | 0.0781 | 0.7282 |
| Q2KIV9 | *C1QB* | complement C1q B chain | 7.01 | -0.74 | 3.03 | 0.0816 | 0.7508 |
| F6RQC9 | *GNAI2* | G protein subunit alpha i2 | 8.14 | 0.67 | 3.02 | 0.0821 | 0.7508 |
| A0A452DJA9 | *NA* | NA | 8.07 | -1.87 | 3.01 | 0.0828 | 0.7508 |
| G3N2F2 | *P4HA2* | prolyl 4-hydroxylase subunit alpha 2 | 4.67 | -1.50 | 2.99 | 0.0838 | 0.7508 |
| A0AAA9TDX3 | *B3GAT3* | beta-1,3-glucuronyltransferase 3 | 6.03 | -1.53 | 2.98 | 0.0843 | 0.7508 |
| A0AAA9SST4 | *ACTR1A* | actin related protein 1A | 4.88 | -1.17 | 2.97 | 0.0848 | 0.7508 |
| P34955 | *SERPINA1* | serpin family A member 1 | 8.60 | -1.29 | 2.96 | 0.0854 | 0.7508 |
| F1MSI2 | *AGRN* | agrin | 4.00 | -1.55 | 2.93 | 0.0869 | 0.7551 |
| A0AAA9S490 | *CLU* | clusterin | 13.50 | -1.02 | 2.92 | 0.0872 | 0.7551 |
| A1L5C6 | *HPN* | hepsin | 8.74 | -0.77 | 2.90 | 0.0886 | 0.7605 |
| A0AAA9SXD6 | *CHPF2* | chondroitin polymerizing factor 2 | 5.21 | -1.42 | 2.88 | 0.0899 | 0.7646 |
| A0AAA9T6M9 | *AGPAT1* | 1-acylglycerol-3-phosphate O-acyltransferase 1 | 7.44 | -1.39 | 2.85 | 0.0911 | 0.7646 |
| A0AAA9TBW3 | *LRG1* | leucine rich alpha-2-glycoprotein 1 | 5.88 | -1.22 | 2.85 | 0.0916 | 0.7646 |
| P02662 | *CSN1S1* | casein alpha s1 | 15.67 | -1.34 | 2.84 | 0.0919 | 0.7646 |
| F1MY85 | *C5* | complement C5 | 9.65 | -1.37 | 2.81 | 0.0937 | 0.7734 |
| F6R1P1 | *TOR3A* | torsin family 3 member A | 6.96 | -1.28 | 2.79 | 0.0949 | 0.7734 |
| G3N1H5 | *NA* | NA | 3.51 | -1.37 | 2.79 | 0.0951 | 0.7734 |
| P27479 | *ALOX15* | arachidonate 15-lipoxygenase | 4.26 | 1.56 | 2.73 | 0.0983 | 0.7854 |
| A0A3Q1LZZ0 | *ATP6AP1* | ATPase H+ transporting accessory protein 1 | 5.95 | -1.21 | 2.73 | 0.0983 | 0.7854 |
| Q4GZT4 | *ABCG2* | NA | 12.00 | 0.65 | 2.72 | 0.0994 | 0.7854 |
| F1MVK1 | *NA* | NA | 5.47 | -1.13 | 2.71 | 0.0994 | 0.7854 |
| A0AAA9TVF8 | *ADAM10* | ADAM metallopeptidase domain 10 | 9.69 | -0.86 | 2.59 | 0.1072 | 0.8005 |
| P01966 | *HBA* | NA | 9.68 | -1.51 | 2.59 | 0.1073 | 0.8005 |
| A0A3Q1LI72 | *MAN2A2* | mannosidase alpha class 2A member 2 | 5.78 | -1.20 | 2.59 | 0.1073 | 0.8005 |
| A0AAA9TM63 | *C1QB* | complement C1q B chain | 5.70 | -1.02 | 2.59 | 0.1074 | 0.8005 |
| G3MXZ7 | *NAGLU* | N-acetyl-alpha-glucosaminidase | 6.63 | -1.25 | 2.58 | 0.1081 | 0.8005 |
| A0A3Q1LPG0 | *NA* | NA | 11.25 | -1.43 | 2.58 | 0.1081 | 0.8005 |
| Q5EA01 | *B4GAT1* | beta-1,4-glucuronyltransferase 1 | 5.97 | -1.11 | 2.58 | 0.1084 | 0.8005 |
| Q3ZBS7 | *VTN* | vitronectin | 8.47 | -0.92 | 2.58 | 0.1085 | 0.8005 |
| P08037 | *B4GALT1* | beta-1,4-galactosyltransferase 1 | 11.35 | -1.21 | 2.57 | 0.1091 | 0.8005 |
| A0AAF6Z3H7 | *CTSS* | NA | 5.66 | -1.33 | 2.56 | 0.1096 | 0.8005 |
| A0A3Q1MGB8 | *AMBP* | alpha-1-microglobulin/bikunin precursor | 4.09 | -1.59 | 2.54 | 0.1113 | 0.8005 |
| A0AAA9TVX5 | *CFH* | complement factor H | 2.61 | -1.74 | 2.53 | 0.1120 | 0.8005 |
| A0AAA9TAD8 | *ADK* | adenosine kinase | 4.15 | 0.92 | 2.53 | 0.1120 | 0.8005 |
| Q8SQ28 | *SAA3* | NA | 7.89 | -0.79 | 2.52 | 0.1121 | 0.8005 |
| F1MM32 | *QSOX1* | quiescin sulfhydryl oxidase 1 | 7.30 | -1.33 | 2.52 | 0.1125 | 0.8005 |
| A0A3Q1M3Z5 | *CTSD* | cathepsin D | 6.71 | -1.11 | 2.51 | 0.1132 | 0.8005 |
| F1MD77 | *LAMC1* | laminin subunit gamma 1 | 4.43 | -1.05 | 2.50 | 0.1140 | 0.8013 |
| A4FV54 | *RAB8A* | RAB8A, member RAS onco family | 7.59 | 0.69 | 2.44 | 0.1180 | 0.8235 |
| A0A3Q1MUV9 | *INHCA* | inhibitor of carbonic anhydrase | 7.77 | -1.38 | 2.43 | 0.1193 | 0.8244 |
| Q95JC7 | *SLC1A5* | solute carrier family 1 member 5 | 6.51 | -1.06 | 2.42 | 0.1196 | 0.8244 |
| Q3ZBZ8 | *STIP1* | NA | 5.47 | 0.80 | 2.39 | 0.1219 | 0.8316 |
| F1MJW4 | *FAM20A* | FAM20A golgi associated secretory pathway pseudokinase | 6.18 | -1.08 | 2.38 | 0.1230 | 0.8316 |
| P10152 | *ANG1* | NA | 3.45 | -1.50 | 2.37 | 0.1235 | 0.8316 |
| A0AAA9TNC9 | *LRRC26* | leucine rich repeat containing 26 | 5.29 | -1.32 | 2.37 | 0.1237 | 0.8316 |
| P10790 | *FABP3* | fatty acid binding protein 3 | 10.76 | 1.07 | 2.33 | 0.1268 | 0.8415 |
| A2I7N3 | *SERPINA3-7* | NA | 10.97 | -1.57 | 2.33 | 0.1271 | 0.8415 |
| A0AAF6YZK4 | *SPCS3* | NA | 6.49 | -1.29 | 2.32 | 0.1275 | 0.8415 |
| A6QLB7 | *CAP1* | cyclase associated actin cytoskeleton regulatory protein 1 | 6.50 | 0.82 | 2.30 | 0.1292 | 0.8457 |
| P37980 | *PPA1* | inorganic pyrophosphatase 1 | 6.32 | 0.69 | 2.28 | 0.1311 | 0.8457 |
| A0A3Q1MIB0 | *IQGAP1* | IQ motif containing GTPase activating protein 1 | 2.63 | 1.34 | 2.27 | 0.1319 | 0.8457 |
| A0AAA9SKJ4 | *NA* | NA | 4.23 | -1.10 | 2.25 | 0.1334 | 0.8457 |
| A0A3Q1MRQ2 | *NA* | NA | 4.21 | -1.72 | 2.25 | 0.1336 | 0.8457 |
| F1MXG6 | *KRT33A* | keratin 33A | 4.31 | -3.10 | 2.25 | 0.1340 | 0.8457 |
| A0A3Q1MUN0 | *SORD* | sorbitol dehydrogenase | 3.87 | 0.97 | 2.23 | 0.1357 | 0.8457 |
| A0AAA9T4C8 | *MATN2* | matrilin 2 | 6.10 | -1.14 | 2.21 | 0.1368 | 0.8457 |
| P81125 | *NAPA* | NA | 7.44 | 0.57 | 2.21 | 0.1370 | 0.8457 |
| A0AAA9STV6 | *LGALS3* | galectin 3 | 2.84 | 1.11 | 2.21 | 0.1373 | 0.8457 |
| A0AAA9T3I7 | *B4GALT4* | beta-1,4-galactosyltransferase 4 | 4.97 | -1.36 | 2.21 | 0.1374 | 0.8457 |
| E1BMM8 | *CHPF* | chondroitin polymerizing factor | 4.03 | -1.31 | 2.20 | 0.1378 | 0.8457 |
| Q58CW5 | *SERINC2* | serine incorporator 2 | 5.29 | -1.08 | 2.18 | 0.1400 | 0.8457 |
| P01045 | *KNG2* | NA | 5.94 | -0.96 | 2.17 | 0.1409 | 0.8457 |
| A8YXY3 | *SELENOF* | selenoprotein F | 5.59 | -1.01 | 2.16 | 0.1414 | 0.8457 |
| F1N780 | *EPHA7* | EPH receptor A7 | 3.10 | -1.29 | 2.14 | 0.1435 | 0.8457 |
| Q8SPP7 | *PGLYRP1* | peptidoglycan recognition protein 1 | 8.14 | -0.87 | 2.14 | 0.1439 | 0.8457 |
| C4T7Z1 | *LY96* | lymphocyte antigen 96 | 7.06 | -0.81 | 2.13 | 0.1443 | 0.8457 |
| A0AAA9TGC0 | *MASP1* | MBL associated serine protease 1 | 4.20 | -1.57 | 2.10 | 0.1474 | 0.8457 |
| A0A452DHZ5 | *NUCB1* | NA | 4.87 | -1.03 | 2.09 | 0.1478 | 0.8457 |
| A0AAA9TR33 | *PDGFC* | platelet derived growth factor C | 8.45 | -1.08 | 2.09 | 0.1486 | 0.8457 |
| A0AAA9SG26 | *STX3* | syntaxin 3 | 8.25 | 0.61 | 2.08 | 0.1492 | 0.8457 |
| F1MET0 | *PLOD3* | procollagen-lysine,2-oxoglutarate 5-dioxygenase 3 | 6.75 | -1.31 | 2.07 | 0.1502 | 0.8457 |
| F1MSZ6 | *SERPINC1* | serpin family C member 1 | 5.27 | -1.03 | 2.07 | 0.1502 | 0.8457 |
| Q2KJJ8 | *PLD3* | phospholipase D family member 3 | 4.65 | -0.84 | 2.07 | 0.1503 | 0.8457 |
| F6R4P6 | *SERPIND1* | serpin family D member 1 | 9.72 | -1.15 | 2.07 | 0.1507 | 0.8457 |
| A0AAA9RZ65 | *SLC31A1* | solute carrier family 31 member 1 | 6.58 | 1.20 | 2.06 | 0.1515 | 0.8457 |
| Q0V8R6 | *HEXA* | hexosaminidase subunit alpha | 4.71 | -1.21 | 2.05 | 0.1520 | 0.8457 |
| Q2YDJ4 | *HINT3* | NA | 3.87 | 0.81 | 2.03 | 0.1545 | 0.8457 |
| A0A3Q1LND7 | *NA* | NA | 4.56 | 0.84 | 2.02 | 0.1552 | 0.8457 |
| A0AAA9RW27 | *OSTF1* | osteoclast stimulating factor 1 | 4.38 | 0.78 | 2.01 | 0.1561 | 0.8457 |
| A0AAF6Z3Q2 | *UBA1* | NA | 4.18 | -1.05 | 2.01 | 0.1567 | 0.8457 |
| A0AAA9TQH8 | *TSPAN9* | tetraspanin 9 | 7.77 | -1.06 | 2.00 | 0.1576 | 0.8457 |
| Q08DW9 | *ABHD12* | NA | 4.39 | -1.19 | 1.99 | 0.1582 | 0.8457 |
| E1B9R3 | *UGGT1* | UDP-glucose glycoprotein glucosyltransferase 1 | 5.97 | -1.14 | 1.99 | 0.1584 | 0.8457 |
| A0AAA9T4F5 | *PDCD6* | programmed cell death 6 | 7.99 | -0.81 | 1.98 | 0.1589 | 0.8457 |
| A0AAF7AP21 | *GNAS* | NA | 8.11 | 0.57 | 1.98 | 0.1593 | 0.8457 |
| A0AAA9S0T2 | *SERPINF2* | serpin family F member 2 | 7.77 | -1.05 | 1.98 | 0.1595 | 0.8457 |
| G5E5M9 | *PI15* | peptidase inhibitor 15 | 3.77 | -1.30 | 1.97 | 0.1602 | 0.8457 |
| Q9BGI2 | *PRDX4* | peroxiredoxin 4 | 8.63 | -0.84 | 1.97 | 0.1606 | 0.8457 |
| F1MK52 | *PROM1* | prominin 1 | 10.69 | -0.79 | 1.97 | 0.1607 | 0.8457 |
| A0AAA9SM26 | *FUCA1* | alpha-L-fucosidase 1 | 5.72 | -1.08 | 1.96 | 0.1612 | 0.8457 |
| A0AAA9S7F8 | *GNA13* | G protein subunit alpha 13 | 4.10 | 0.79 | 1.96 | 0.1618 | 0.8457 |
| A0AAF7A4E2 | *ADPRH* | NA | 3.85 | -1.33 | 1.93 | 0.1647 | 0.8568 |
| Q29RQ1 | *C7* | NA | 6.48 | -1.22 | 1.92 | 0.1655 | 0.8568 |
| A0AAA9SH17 | *PRADC1* | protease associated domain containing 1 | 6.76 | -1.02 | 1.88 | 0.1706 | 0.8746 |
| F1MM83 | *PGLS* | 6-phosphogluconolactonase | 4.79 | 0.85 | 1.87 | 0.1710 | 0.8746 |
| A5D984 | *PKM* | pyruvate kinase M1/2 | 7.19 | 0.59 | 1.86 | 0.1728 | 0.8746 |
| E1BA17 | *COL14A1* | collagen type XIV alpha 1 chain | 9.11 | -1.10 | 1.85 | 0.1737 | 0.8746 |
| G3X6G8 | *LGR6* | leucine rich repeat containing G protein-coupled receptor 6 | 8.84 | -0.83 | 1.85 | 0.1739 | 0.8746 |
| P06623 | *CNP* | 2',3'-cyclic nucleotide 3' phosphodiesterase | 6.36 | 0.50 | 1.83 | 0.1764 | 0.8746 |
| P13752 | *NA* | NA | 10.39 | 0.62 | 1.82 | 0.1775 | 0.8746 |
| P00727 | *LAP3* | leucine aminopeptidase 3 | 8.67 | 0.50 | 1.81 | 0.1790 | 0.8746 |
| Q32LJ4 | *TPRG1L* | NA | 5.17 | -0.83 | 1.80 | 0.1800 | 0.8746 |
| A0AAF6YTE2 | *NSDHL* | NA | 4.65 | 0.69 | 1.79 | 0.1812 | 0.8746 |
| A0AAA9TL27 | *NECTIN4* | nectin cell adhesion molecule 4 | 2.68 | -1.26 | 1.78 | 0.1816 | 0.8746 |
| A0AAA9SG32 | *KCTD12* | potassium channel tetramerization domain containing 12 | 1.20 | 1.26 | 1.78 | 0.1819 | 0.8746 |
| F1MWX3 | *NA* | NA | 6.20 | -1.23 | 1.78 | 0.1827 | 0.8746 |
| F6QK60 | *SIRT2* | sirtuin 2 | 1.99 | 1.20 | 1.77 | 0.1835 | 0.8746 |
| F1MRM8 | *GPRC5C* | G protein-coupled receptor class C group 5 member C | 9.14 | -1.31 | 1.77 | 0.1835 | 0.8746 |
| Q3SYS6 | *CHP1* | calcineurin like EF-hand protein 1 | 4.52 | 0.82 | 1.76 | 0.1840 | 0.8746 |
| Q3SX46 | *C1GALT1C1* | C1GALT1 specific chaperone 1 | 6.93 | -1.05 | 1.76 | 0.1840 | 0.8746 |
| P81134 | *ATP6AP2* | ATPase H+ transporting accessory protein 2 | 8.55 | -0.96 | 1.76 | 0.1849 | 0.8746 |
| A0AAA9SME9 | *POGLUT2* | protein O-glucosyltransferase 2 | 2.79 | -1.40 | 1.75 | 0.1860 | 0.8746 |
| Q29RU0 | *RNF128* | NA | 4.43 | -0.64 | 1.74 | 0.1869 | 0.8746 |
| F6QPI1 | *NDNF* | neuron derived neurotrophic factor | 3.55 | -1.16 | 1.74 | 0.1872 | 0.8746 |
| P62248 | *MYDGF* | myeloid derived growth factor | 3.40 | -1.21 | 1.74 | 0.1875 | 0.8746 |
| Q58CS8 | *GNPTG* | NA | 4.08 | -1.09 | 1.73 | 0.1884 | 0.8746 |
| A0AAA9TMK4 | *APMAP* | adipocyte plasma membrane associated protein | 6.44 | -1.02 | 1.72 | 0.1892 | 0.8746 |
| G1K122 | *RBP4* | retinol binding protein 4 | 4.89 | -1.19 | 1.72 | 0.1901 | 0.8746 |
| A0A3Q1M8N6 | *ABO* | ABO, alpha 1-3-N-acetylgalactosaminyltransferase and alpha 1-3-galactosyltransferase | 4.81 | -1.03 | 1.71 | 0.1911 | 0.8746 |
| F1N0M7 | *B4GALNT3* | beta-1,4-N-acetyl-galactosaminyltransferase 3 | 7.46 | -1.01 | 1.69 | 0.1938 | 0.8746 |
| P19803 | *ARHGDIA* | NA | 8.15 | 0.50 | 1.68 | 0.1951 | 0.8746 |
| P46193 | *ANXA1* | NA | 8.75 | 0.76 | 1.68 | 0.1952 | 0.8746 |
| A0AAA9U0S2 | *PTPRK* | protein tyrosine phosphatase receptor type K | 2.76 | -1.17 | 1.68 | 0.1953 | 0.8746 |
| A0A3Q1MDA4 | *DAG1* | dystroglycan 1 | 9.07 | -1.71 | 1.67 | 0.1957 | 0.8746 |
| A0AAA9SK87 | *ITFG1* | integrin alpha FG-GAP repeat containing 1 | 3.23 | -0.93 | 1.66 | 0.1976 | 0.8746 |
| F1N4W0 | *PIGT* | phosphatidylinositol glycan anchor biosynthesis class T | 5.40 | -1.11 | 1.66 | 0.1977 | 0.8746 |
| O19131 | *TNFRSF1A* | NA | 4.36 | -0.86 | 1.66 | 0.1979 | 0.8746 |
| F6RAW2 | *SPINT1* | serine peptidase inhibitor, Kunitz type 1 | 2.60 | -1.06 | 1.64 | 0.2002 | 0.8746 |
| A0A3Q1M5V0 | *NAPG* | NSF attachment protein gamma | 4.98 | 0.61 | 1.64 | 0.2004 | 0.8746 |
| A0A3Q1LGL7 | *LAMA4* | laminin subunit alpha 4 | 1.95 | -2.45 | 1.64 | 0.2010 | 0.8746 |
| Q3SYU2 | *EEF2* | eukaryotic translation elongation factor 2 | 7.42 | 0.52 | 1.63 | 0.2013 | 0.8746 |
| F1MJQ1 | *RAB7A* | RAB7A, member RAS onco family | 5.04 | 0.56 | 1.62 | 0.2026 | 0.8746 |
| Q3T178 | *VPS28* | NA | 8.18 | -0.85 | 1.61 | 0.2041 | 0.8746 |
| Q2KIG3 | *CPB2* | NA | 1.92 | -1.45 | 1.61 | 0.2044 | 0.8746 |
| A0A3S5ZPJ7 | *GFUS* | GDP-L-fucose synthase | 6.46 | 0.54 | 1.61 | 0.2045 | 0.8746 |
| A0AAF6Z8P4 | *SNAP29* | NA | 4.82 | 0.83 | 1.61 | 0.2049 | 0.8746 |
| A0A3Q1MR41 | *ADAM15* | ADAM metallopeptidase domain 15 | 3.94 | -1.18 | 1.60 | 0.2055 | 0.8746 |
| A6QLY9 | *EVA1B* | eva-1 homolog B | 6.31 | -1.05 | 1.60 | 0.2058 | 0.8746 |
| A0AAA9TRE9 | *SERPINF1* | serpin family F member 1 | 9.10 | -1.07 | 1.60 | 0.2061 | 0.8746 |
| F1MM86 | *C6* | complement C6 | 4.53 | -1.20 | 1.58 | 0.2082 | 0.8770 |
| Q17QE5 | *CIB1* | calcium and integrin binding 1 | 6.14 | -0.89 | 1.58 | 0.2089 | 0.8770 |
| Q29460 | *PAFAH1B3* | NA | 4.32 | 0.65 | 1.57 | 0.2103 | 0.8770 |
| Q2KJ41 | *TSPAN14* | tetraspanin 14 | 6.82 | -0.67 | 1.57 | 0.2105 | 0.8770 |
| G5E6P3 | *GNA13* | G protein subunit alpha 13 | 8.85 | 0.47 | 1.57 | 0.2109 | 0.8770 |
| P17248 | *WARS1* | tryptophanyl-tRNA synthetase 1 | 8.74 | 0.51 | 1.56 | 0.2115 | 0.8770 |
| A0A3Q1M7Q5 | *C1S* | complement C1s | 7.09 | -0.76 | 1.55 | 0.2127 | 0.8778 |
| F1MHC2 | *STXBP2* | syntaxin binding protein 2 | 7.92 | 0.53 | 1.55 | 0.2136 | 0.8778 |
| Q0VCQ6 | *PDCD10* | programmed cell death 10 | 7.39 | 0.50 | 1.54 | 0.2147 | 0.8778 |
| Q5E9A6 | *VPS25* | NA | 6.04 | -0.67 | 1.53 | 0.2160 | 0.8778 |
| A0AAA9T6U4 | *TAPBP* | TAP binding protein | 4.34 | -1.00 | 1.53 | 0.2162 | 0.8778 |
| A0A3Q1LQ02 | *GC* | GC vitamin D binding protein | 5.85 | -0.75 | 1.53 | 0.2166 | 0.8778 |
| E1BM92 | *PROM2* | prominin 2 | 3.30 | -1.01 | 1.50 | 0.2205 | 0.8798 |
| A0A452DI66 | *F2* | coagulation factor II, thrombin | 8.01 | -0.98 | 1.50 | 0.2207 | 0.8798 |
| A0AAA9SDB6 | *SLC3A2* | solute carrier family 3 member 2 | 8.58 | -0.48 | 1.50 | 0.2208 | 0.8798 |
| A0A3Q1MG73 | *FBLN1* | fibulin 1 | 3.06 | -2.30 | 1.50 | 0.2212 | 0.8798 |
| A0AAA9SRF6 | *TGFB2* | transforming growth factor beta 2 | 1.77 | -1.12 | 1.49 | 0.2218 | 0.8798 |
| A0A140T894 | *YWHAB* | tyrosine 3-monooxygenase/tryptophan 5-monooxygenase activation protein beta | 8.00 | 0.48 | 1.48 | 0.2230 | 0.8798 |
| A0A140T8A9 | *CSN3* | casein kappa | 16.89 | -0.84 | 1.48 | 0.2245 | 0.8798 |
| A0AAF7A715 | *ITGB6* | NA | 5.30 | -0.96 | 1.47 | 0.2250 | 0.8798 |
| A0AAF7A7H1 | *MAN2B1* | NA | 4.27 | -1.00 | 1.47 | 0.2255 | 0.8798 |
| Q0VC36 | *SFN* | stratifin | 5.60 | 0.61 | 1.46 | 0.2273 | 0.8798 |
| A0AAA9SJC5 | *GMPR2* | guanosine monophosphate reductase 2 | 2.07 | 0.98 | 1.45 | 0.2281 | 0.8798 |
| E1BEL6 | *NRP2* | neuropilin 2 | 5.31 | 0.60 | 1.45 | 0.2282 | 0.8798 |
| A0AAA9SSQ7 | *RTN3* | reticulon 3 | 2.63 | 0.93 | 1.44 | 0.2294 | 0.8798 |
| F1N152 | *HTRA1* | NA | 7.50 | -0.77 | 1.44 | 0.2295 | 0.8798 |
| A0A3Q1LX93 | *CZIB* | CXXC motif containing zinc binding protein | 2.75 | 0.88 | 1.44 | 0.2296 | 0.8798 |
| A0AAA9SLZ6 | *SLC44A2* | solute carrier family 44 member 2 | 4.30 | -0.83 | 1.43 | 0.2312 | 0.8798 |
| P80457 | *XDH* | xanthine dehydrogenase | 15.64 | 0.50 | 1.43 | 0.2315 | 0.8798 |
| Q0VCN9 | *FOLR2* | folate receptor 2 (fetal) | 10.72 | -0.50 | 1.43 | 0.2322 | 0.8798 |
| F1MX50 | *CREG1* | cellular repressor of E1A stimulateds 1 | 6.05 | -0.78 | 1.43 | 0.2326 | 0.8798 |
| A0A3Q1LQE4 | *ITGB2* | integrin subunit beta 2 | 6.27 | 0.81 | 1.40 | 0.2364 | 0.8805 |
| E1BB36 | *MGAT2* | alpha-1,6-mannosyl-glycoprotein 2-beta-N-acetylglucosaminyltransferase | 5.25 | -1.02 | 1.40 | 0.2373 | 0.8805 |
| F1N7T1 | *CYB5R3* | cytochrome b5 reductase 3 | 9.18 | 0.46 | 1.40 | 0.2373 | 0.8805 |
| A0A3Q1LQU2 | *RNH1* | ribonuclease/angiogenin inhibitor 1 | 4.08 | 0.68 | 1.39 | 0.2377 | 0.8805 |
| Q3MHP2 | *RAB11B* | RAB11B, member RAS onco family | 9.83 | 0.46 | 1.38 | 0.2399 | 0.8805 |
| A0A3Q1N5X6 | *FAM20C* | FAM20C golgi associated secretory pathway kinase | 6.71 | -0.81 | 1.38 | 0.2400 | 0.8805 |
| Q9MZ06 | *FGFBP1* | fibroblast growth factor binding protein 1 | 4.08 | -0.68 | 1.37 | 0.2422 | 0.8805 |
| Q148C9 | *HEBP1* | NA | 5.03 | 0.52 | 1.36 | 0.2428 | 0.8805 |
| F6QE93 | *MLEC* | malectin | 6.51 | -0.83 | 1.36 | 0.2429 | 0.8805 |
| A0AAF6DLT6 | *PON1* | paraoxonase 1 | 6.34 | -1.09 | 1.36 | 0.2431 | 0.8805 |
| A0AAA9RTG5 | *TOR1B* | torsin family 1 member B | 3.58 | -1.24 | 1.36 | 0.2435 | 0.8805 |
| A8E4P3 | *STOM* | stomatin | 6.48 | 1.25 | 1.36 | 0.2435 | 0.8805 |
| P79121 | *TIMP3* | NA | 6.18 | -0.64 | 1.34 | 0.2470 | 0.8805 |
| Q5E9E2 | *MYL9* | NA | 2.67 | -0.92 | 1.34 | 0.2476 | 0.8805 |
| A0AAA9T2W5 | *DNAJA2* | DnaJ heat shock protein family (Hsp40) member A2 | 6.03 | 0.52 | 1.33 | 0.2480 | 0.8805 |
| Q3SZH7 | *LTA4H* | leukotriene A4 hydrolase | 6.74 | 0.63 | 1.33 | 0.2487 | 0.8805 |
| A0A3Q1MGP0 | *PRKCB* | NA | 1.80 | 1.00 | 1.33 | 0.2495 | 0.8805 |
| A0AAA9TJK9 | *CAB39* | calcium binding protein 39 | 7.44 | 0.46 | 1.32 | 0.2510 | 0.8805 |
| F6QJE8 | *PAFAH1B1* | platelet activating factor acetylhydrolase 1b regulatory subunit 1 | 3.18 | 0.89 | 1.32 | 0.2512 | 0.8805 |
| G3N2D8 | *GGT1* | gamma-glutamyltransferase 1 | 12.64 | -0.54 | 1.32 | 0.2513 | 0.8805 |
| A0A3Q1MRJ9 | *NECTIN2* | nectin cell adhesion molecule 2 | 5.23 | -0.59 | 1.32 | 0.2514 | 0.8805 |
| A0A3Q1NIZ0 | *TGFB1* | transforming growth factor beta 1 | 2.13 | -1.38 | 1.31 | 0.2515 | 0.8805 |
| A0AAA9RRF4 | *TSPAN4* | tetraspanin 4 | 2.89 | -0.80 | 1.31 | 0.2528 | 0.8805 |
| Q0VCX2 | *HSPA5* | heat shock protein family A (Hsp70) member 5 | 8.05 | -0.85 | 1.31 | 0.2530 | 0.8805 |
| A0AAF7AF61 | *MYO1C* | NA | 5.63 | 0.63 | 1.30 | 0.2534 | 0.8805 |
| A0A3Q1LUB1 | *PTPRD* | protein tyrosine phosphatase receptor type D | 0.72 | 2.82 | 1.30 | 0.2539 | 0.8805 |
| Q29455 | *DNAJC5* | DnaJ heat shock protein family (Hsp40) member C5 | 4.45 | 0.59 | 1.27 | 0.2606 | 0.8805 |
| A0A3Q1MJ73 | *NA* | NA | 11.43 | -0.75 | 1.26 | 0.2608 | 0.8805 |
| A0AAA9TNQ2 | *RIT1* | Ras like without CAAX 1 | 3.20 | 0.81 | 1.26 | 0.2620 | 0.8805 |
| A0AAF6ZGY7 | *BGN* | NA | 5.53 | -1.32 | 1.26 | 0.2624 | 0.8805 |
| A0AAF6Z0W1 | *CD9* | NA | 13.23 | -0.56 | 1.25 | 0.2640 | 0.8805 |
| Q2YDJ9 | *GMPPB* | NA | 5.47 | 0.56 | 1.24 | 0.2654 | 0.8805 |
| A0AAA9TZS0 | *SERPING1* | serpin family G member 1 | 7.79 | -0.89 | 1.24 | 0.2661 | 0.8805 |
| A0AAA9TEV2 | *PMM2* | phosphomannomutase 2 | 1.78 | 1.03 | 1.23 | 0.2669 | 0.8805 |
| Q3SZ54 | *EIF4A1* | eukaryotic translation initiation factor 4A1 | 6.46 | 0.44 | 1.23 | 0.2669 | 0.8805 |
| A0AAA9SNC1 | *TSG101* | tumor susceptibility 101 | 9.45 | -0.70 | 1.23 | 0.2671 | 0.8805 |
| F1MJU5 | *NA* | NA | 6.15 | 0.78 | 1.23 | 0.2684 | 0.8805 |
| Q32LK2 | *CYSTM1* | NA | 5.00 | -0.88 | 1.21 | 0.2710 | 0.8805 |
| P63103 | *YWHAZ* | tyrosine 3-monooxygenase/tryptophan 5-monooxygenase activation protein zeta | 9.73 | 0.41 | 1.20 | 0.2727 | 0.8805 |
| F1N5W5 | *STK26* | serine/threonine kinase 26 | 5.71 | 0.49 | 1.20 | 0.2735 | 0.8805 |
| P61625 | *ITGAL* | integrin subunit alpha L | 7.27 | -0.93 | 1.20 | 0.2737 | 0.8805 |
| F1MZA0 | *NA* | NA | 8.73 | -0.67 | 1.20 | 0.2741 | 0.8805 |
| Q5EAD2 | *PHGDH* | NA | 3.91 | 0.78 | 1.19 | 0.2745 | 0.8805 |
| E1B725 | *GLA* | galactosidase alpha | 5.84 | -0.90 | 1.19 | 0.2758 | 0.8805 |
| F6S0B8 | *NA* | NA | 5.19 | -0.71 | 1.18 | 0.2764 | 0.8805 |
| P02754 | *LGB* | NA | 12.32 | -0.77 | 1.18 | 0.2765 | 0.8805 |
| P02663 | *CSN1S2* | casein alpha-S2 | 13.24 | -0.75 | 1.18 | 0.2783 | 0.8805 |
| F1MW44 | *F13A1* | coagulation factor XIII A chain | 3.69 | -0.92 | 1.17 | 0.2786 | 0.8805 |
| A0AAA9SNY2 | *MOB1B* | MOB kinase activator 1B | 5.42 | 0.46 | 1.17 | 0.2793 | 0.8805 |
| A0AAA9SGY6 | *FCGBP* | Fc gamma binding protein | 4.95 | -1.06 | 1.17 | 0.2794 | 0.8805 |
| Q0VCH9 | *GLIPR2* | GLI pathosis related 2 | 5.09 | 0.92 | 1.17 | 0.2794 | 0.8805 |
| A5PJL8 | *C1QTNF5* | C1q and TNF related 5 | 4.95 | -0.76 | 1.17 | 0.2795 | 0.8805 |
| P01017 | *AGT* | angiotensinogen | 7.44 | -0.92 | 1.16 | 0.2811 | 0.8805 |
| Q08DK7 | *SLC25A29* | solute carrier family 25 member 29 | 4.42 | -0.96 | 1.16 | 0.2814 | 0.8805 |
| Q3MHL4 | *AHCY* | adenosylhomocysteinase | 6.59 | 0.43 | 1.16 | 0.2818 | 0.8805 |
| F1N6H1 | *LRP2* | LDL receptor related protein 2 | 9.88 | -0.66 | 1.15 | 0.2826 | 0.8805 |
| F1MM13 | *H6PD* | hexose-6-phosphate dehydrogenase/glucose 1-dehydrogenase | 6.73 | -0.86 | 1.14 | 0.2854 | 0.8805 |
| Q9BDR7 | *FCER1G* | Fc epsilon receptor Ig | 4.61 | 0.54 | 1.14 | 0.2859 | 0.8805 |
| Q2KJ54 | *SPINT2* | serine peptidase inhibitor, Kunitz type 2 | 5.99 | 0.90 | 1.14 | 0.2864 | 0.8805 |
| F1MT69 | *VPS36* | vacuolar protein sorting 36 homolog | 6.26 | -0.57 | 1.13 | 0.2868 | 0.8805 |
| A0A3Q1N570 | *CD46* | NA | 8.63 | -0.55 | 1.13 | 0.2877 | 0.8805 |
| A0AAA9TWN0 | *PEPD* | peptidase D | 5.21 | -0.78 | 1.13 | 0.2884 | 0.8805 |
| Q29RY7 | *FGL2* | fibrinogen like 2 | 5.80 | -0.98 | 1.12 | 0.2904 | 0.8805 |
| P84466 | *LSS* | NA | 4.52 | 0.65 | 1.11 | 0.2917 | 0.8805 |
| Q32KV6 | *SIL1* | SIL1 nucleotide exchange factor | 8.25 | -0.72 | 1.11 | 0.2918 | 0.8805 |
| G3N0S9 | *NA* | NA | 3.81 | -0.84 | 1.11 | 0.2929 | 0.8805 |
| Q3ZCD0 | *CD81* | NA | 11.46 | -0.78 | 1.11 | 0.2931 | 0.8805 |
| F1MDQ6 | *PRKG2* | protein kinase cGMP-dependent 2 | 3.46 | 1.81 | 1.10 | 0.2941 | 0.8805 |
| F1MXH7 | *CSF2RB* | colony stimulating factor 2 receptor subunit beta | 2.48 | -0.99 | 1.10 | 0.2941 | 0.8805 |
| P13135 | *CAPNS1* | NA | 2.83 | 0.73 | 1.10 | 0.2943 | 0.8805 |
| A0A452DKH0 | *PCBP1* | poly(rC) binding protein 1 | 5.17 | 0.48 | 1.10 | 0.2945 | 0.8805 |
| A4FUA8 | *CAPZA1* | capping actin protein of muscle Z-line subunit alpha 1 | 6.86 | 0.49 | 1.10 | 0.2951 | 0.8805 |
| A0AAA9RYS2 | *ATP1A1* | ATPase Na+/K+ transporting subunit alpha 1 | 5.39 | 0.48 | 1.09 | 0.2956 | 0.8805 |
| A0A3Q1M8J9 | *GAPDH* | glyceraldehyde-3-phosphate dehydrogenase | 8.76 | 0.40 | 1.09 | 0.2958 | 0.8805 |
| A0AAA9ST74 | *SDCBP* | syndecan binding protein | 10.93 | -0.64 | 1.09 | 0.2961 | 0.8805 |
| P62871 | *GNB1* | G protein subunit beta 1 | 9.26 | 0.41 | 1.09 | 0.2965 | 0.8805 |
| A0AAA9TBM6 | *IGFALS* | insulin like growth factor binding protein acid labile subunit | 4.33 | -0.98 | 1.09 | 0.2966 | 0.8805 |
| P50397 | *GDI2* | GDP dissociation inhibitor 2 | 10.52 | 0.38 | 1.09 | 0.2968 | 0.8805 |
| Q5EAB4 | *CHID1* | chitinase domain containing 1 | 7.52 | -0.79 | 1.08 | 0.2978 | 0.8805 |
| A0AAA9T4S8 | *ITGAV* | integrin subunit alpha V | 6.38 | -0.66 | 1.08 | 0.2981 | 0.8805 |
| Q3T054 | *RAN* | NA | 5.45 | 0.49 | 1.07 | 0.3020 | 0.8805 |
| E1BJV0 | *EHD4* | EH domain containing 4 | 8.10 | 0.45 | 1.06 | 0.3022 | 0.8805 |
| E1BNW4 | *TMC4* | transmembrane channel like 4 | 5.64 | -0.74 | 1.06 | 0.3030 | 0.8805 |
| P80195 | *GLYCAM1* | glycosylation dependent cell adhesion molecule 1 | 8.25 | -0.75 | 1.06 | 0.3035 | 0.8805 |
| Q3T0L2 | *ERP44* | endoplasmic reticulum protein 44 | 3.20 | -2.05 | 1.06 | 0.3035 | 0.8805 |
| A7MBJ4 | *PTPRF* | protein tyrosine phosphatase receptor type F | 3.75 | -0.67 | 1.06 | 0.3037 | 0.8805 |
| A0A3Q1MPD6 | *ZNF185* | zinc finger protein 185 with LIM domain | 4.48 | 0.62 | 1.06 | 0.3040 | 0.8805 |
| P50227 | *SULT1A1* | sulfotransferase family, cytosolic, 1A, phenol-preferring, member 1 | 4.77 | 0.45 | 1.06 | 0.3043 | 0.8805 |
| Q2KIS4 | *DHRS1* | dehydrogenase/reductase 1 | 8.17 | 0.39 | 1.05 | 0.3056 | 0.8816 |
| F1MKU3 | *BSPRY* | B-box and SPRY domain containing | 5.25 | 0.53 | 1.04 | 0.3082 | 0.8829 |
| A0A3Q1M1B6 | *CD177* | CD177 molecule | 4.34 | 0.90 | 1.04 | 0.3085 | 0.8829 |
| Q3T0R1 | *RPS18* | NA | 3.06 | 1.14 | 1.03 | 0.3090 | 0.8829 |
| G3MXB5 | *NA* | NA | 12.23 | -0.75 | 1.03 | 0.3093 | 0.8829 |
| Q0VFX8 | *CRIP2* | NA | 1.40 | 3.19 | 1.02 | 0.3121 | 0.8879 |
| A0A3S5ZPG9 | *MUC15* | mucin 15, cell surface associated | 9.08 | 1.06 | 1.02 | 0.3132 | 0.8879 |
| A0AAA9TK47 | *GLOD4* | glyoxalase domain containing 4 | 4.71 | 0.50 | 1.02 | 0.3135 | 0.8879 |
| F1MD47 | *C6H4orf19* | chromosome 6 C4orf19 homolog | 5.74 | 0.41 | 1.01 | 0.3149 | 0.8882 |
| Q58DS5 | *RAB13* | NA | 5.01 | 0.49 | 1.01 | 0.3152 | 0.8882 |
| Q3SZK8 | *NHERF1* | NHERF family PDZ scaffold protein 1 | 8.77 | 0.48 | 1.00 | 0.3163 | 0.8890 |
| Q3ZC13 | *AP2M1* | adaptor related protein complex 2 subunit mu 1 | 2.70 | 1.75 | 1.00 | 0.3181 | 0.8916 |
| A5PK45 | *COLGALT1* | collagen beta(1-O)galactosyltransferase 1 | 6.20 | -0.79 | 0.99 | 0.3196 | 0.8935 |
| A0AAA9T9N5 | *NA* | NA | 4.42 | 1.96 | 0.99 | 0.3208 | 0.8937 |
| Q5EA88 | *GPD1* | NA | 3.47 | 0.73 | 0.98 | 0.3213 | 0.8937 |
| A6QLS9 | *RAB10* | RAB10, member RAS onco family | 10.44 | 0.36 | 0.97 | 0.3237 | 0.8981 |
| A6QLY4 | *ISOC1* | NA | 4.38 | -0.48 | 0.97 | 0.3251 | 0.8994 |
| P68138 | *ACTA1* | actin alpha 1, skeletal muscle | 7.80 | -0.49 | 0.96 | 0.3260 | 0.8994 |
| Q6EWQ7 | *EIF5A* | eukaryotic translation initiation factor 5A | 4.14 | 0.62 | 0.96 | 0.3267 | 0.8994 |
| Q0VCP3 | *OLFML3* | NA | 3.79 | -0.92 | 0.96 | 0.3282 | 0.9013 |
| A0AAA9TCS0 | *ENO1* | enolase 1 | 8.88 | 0.41 | 0.95 | 0.3302 | 0.9015 |
| Q58CQ2 | *ARPC1B* | actin related protein 2/3 complex subunit 1B | 5.95 | 0.43 | 0.95 | 0.3306 | 0.9015 |
| Q2TBX4 | *HSPA13* | heat shock protein family A (Hsp70) member 13 | 5.16 | -0.87 | 0.94 | 0.3331 | 0.9015 |
| A0AAA9T2W8 | *SDS* | serine dehydratase | 4.20 | 0.98 | 0.93 | 0.3340 | 0.9015 |
| P25417 | *CSTB* | NA | 6.05 | 0.41 | 0.93 | 0.3346 | 0.9015 |
| F6QPB9 | *HLA-DRA* | major histocompatibility complex, class II, DR alpha | 5.78 | 0.86 | 0.93 | 0.3348 | 0.9015 |
| A0AAA9TDC9 | *PCMT1* | protein-L-isoaspartate (D-aspartate) O-methyltransferase | 4.56 | -0.83 | 0.92 | 0.3365 | 0.9015 |
| Q1RMI2 | *RHOG* | ras homolog family member G | 7.83 | 0.68 | 0.92 | 0.3375 | 0.9015 |
| P18203 | *FKBP1A* | FKBP prolyl isomerase 1A | 2.91 | -1.80 | 0.92 | 0.3380 | 0.9015 |
| F1N3P2 | *USP5* | ubiquitin specific peptidase 5 | 1.76 | 1.68 | 0.92 | 0.3385 | 0.9015 |
| Q3ZCJ2 | *AKR1A1* | aldo-keto reductase family 1 member A1 | 6.85 | 0.38 | 0.92 | 0.3388 | 0.9015 |
| Q0VCB5 | *HS3ST1* | heparan sulfate-glucosamine 3-sulfotransferase 1 | 5.87 | -0.74 | 0.91 | 0.3403 | 0.9015 |
| A0A3Q1LQY1 | *CNTFR* | ciliary neurotrophic factor receptor | 6.37 | -0.75 | 0.90 | 0.3429 | 0.9015 |
| A0AAA9U299 | *TMEM30A* | transmembrane protein 30A | 5.36 | 0.41 | 0.90 | 0.3436 | 0.9015 |
| P13753 | *NA* | NA | 8.78 | -0.77 | 0.90 | 0.3439 | 0.9015 |
| Q28065 | *C4BPA* | NA | 2.58 | -1.23 | 0.89 | 0.3457 | 0.9015 |
| A0A452DJ96 | *PGM1* | phosphoglucomutase 1 | 6.50 | 0.35 | 0.89 | 0.3459 | 0.9015 |
| A0AAF6Z5S1 | *NA* | NA | 4.59 | -2.51 | 0.89 | 0.3461 | 0.9015 |
| Q95135 | *SLC1A1* | NA | 6.36 | -0.41 | 0.89 | 0.3463 | 0.9015 |
| A0AAA9RWN7 | *PSPH* | phosphoserine phosphatase | 4.21 | 0.59 | 0.88 | 0.3469 | 0.9015 |
| A0A3Q1M1A5 | *HYOU1* | hypoxia up-regulated 1 | 3.74 | -0.83 | 0.88 | 0.3482 | 0.9015 |
| A0A3Q1LZC9 | *LAMP2* | lysosomal associated membrane protein 2 | 5.72 | -0.60 | 0.87 | 0.3497 | 0.9015 |
| Q3SZB7 | *FBP1* | fructose-bisphosphatase 1 | 3.11 | -0.74 | 0.87 | 0.3512 | 0.9015 |
| E1BGX8 | *HHIPL2* | HHIP like 2 | 10.56 | -0.71 | 0.87 | 0.3513 | 0.9015 |
| A0A140T8A5 | *IDH1* | isocitrate dehydrogenase (NADP(+)) 1 | 10.98 | 0.37 | 0.87 | 0.3517 | 0.9015 |
| F1MQD1 | *CAPN5* | calpain 5 | -0.27 | 2.79 | 0.87 | 0.3519 | 0.9015 |
| Q3SX14 | *GSN* | NA | 7.83 | -0.66 | 0.86 | 0.3525 | 0.9015 |
| F1N076 | *CP* | ceruloplasmin | 8.86 | -0.71 | 0.86 | 0.3530 | 0.9015 |
| F1MUT3 | *XDH* | xanthine dehydrogenase | 5.98 | 0.76 | 0.86 | 0.3538 | 0.9015 |
| A0A3Q1M9W0 | *NDRG1* | N-myc downstream regulated 1 | 5.96 | 0.42 | 0.86 | 0.3542 | 0.9015 |
| A0AAA9SQE0 | *SND1* | staphylococcal nuclease and tudor domain containing 1 | 2.67 | 0.72 | 0.85 | 0.3552 | 0.9015 |
| Q9XSA7 | *CLIC4* | chloride intracellular channel 4 | 8.66 | 0.46 | 0.85 | 0.3552 | 0.9015 |
| P00711 | *LALBA* | NA | 8.00 | -0.45 | 0.85 | 0.3558 | 0.9015 |
| F1N3A1 | *THBS1* | thrombospondin 1 | 6.88 | -0.64 | 0.85 | 0.3574 | 0.9026 |
| A5PKL2 | *RRAS2* | RAS related 2 | 4.26 | 0.71 | 0.84 | 0.3584 | 0.9026 |
| A0A3Q1MGP1 | *C1R* | complement C1r | 6.99 | -0.65 | 0.84 | 0.3594 | 0.9026 |
| A0A3Q1MQM0 | *TOR1A* | torsin family 1 member A | 3.28 | -0.80 | 0.84 | 0.3600 | 0.9026 |
| A0A3Q1LW01 | *CNDP2* | carnosine dipeptidase 2 | 5.41 | 0.40 | 0.84 | 0.3604 | 0.9026 |
| F6Q9I3 | *CC2D1A* | coiled-coil and C2 domain containing 1A | 3.51 | -0.70 | 0.83 | 0.3621 | 0.9041 |
| A0A3Q1MNN2 | *AKR7A2* | NA | 3.63 | 0.52 | 0.83 | 0.3636 | 0.9041 |
| A0AAA9SF39 | *NA* | NA | 15.51 | -0.44 | 0.82 | 0.3643 | 0.9041 |
| A0A3Q1LMW6 | *NA* | NA | 4.67 | 1.63 | 0.82 | 0.3643 | 0.9041 |
| Q17QH1 | *APOF* | apolipoprotein F | 4.26 | -0.47 | 0.82 | 0.3657 | 0.9054 |
| F6Q751 | *NA* | NA | 5.92 | 0.44 | 0.81 | 0.3671 | 0.9058 |
| A0AAA9RWM7 | *TPM3* | tropomyosin 3 | 3.45 | 0.60 | 0.81 | 0.3675 | 0.9058 |
| A0AAF6YH77 | *YKT6* | NA | 8.30 | 0.36 | 0.81 | 0.3692 | 0.9078 |
| Q1JPD9 | *GPRC5B* | G protein-coupled receptor class C group 5 member B | 11.19 | -0.67 | 0.80 | 0.3709 | 0.9099 |
| A5PJM4 | *AIFM2* | apoptosis inducing factor mitochondria associated 2 | 6.10 | 0.41 | 0.80 | 0.3719 | 0.9103 |
| P61223 | *RAP1B* | RAP1B, member of RAS onco family | 10.04 | 0.37 | 0.79 | 0.3745 | 0.9131 |
| Q3B7M5 | *LASP1* | LIM and SH3 protein 1 | 4.26 | 0.68 | 0.79 | 0.3748 | 0.9131 |
| A2VE47 | *ADPGK* | ADP dependent glucokinase | 5.56 | -0.59 | 0.79 | 0.3755 | 0.9131 |
| F1N036 | *DNAJC3* | DnaJ heat shock protein family (Hsp40) member C3 | 7.95 | -0.68 | 0.78 | 0.3783 | 0.9148 |
| A0A3Q1MUJ8 | *PLXNB2* | plexin B2 | 4.69 | -0.45 | 0.78 | 0.3785 | 0.9148 |
| A0A3S5ZPM3 | *PGD* | phosphogluconate dehydrogenase | 7.61 | -0.40 | 0.77 | 0.3788 | 0.9148 |
| Q5E963 | *ARPC5L* | actin related protein 2/3 complex subunit 5 like | 3.89 | -0.48 | 0.76 | 0.3824 | 0.9196 |
| A0A3Q1MQM3 | *ST13* | ST13 Hsp70 interacting protein | 8.23 | 0.36 | 0.76 | 0.3825 | 0.9196 |
| Q3ZBW4 | *PCNA* | proliferating cell nuclear antigen | 2.62 | 0.72 | 0.76 | 0.3837 | 0.9199 |
| F2Z4F5 | *DPP3* | dipeptidyl peptidase 3 | 3.17 | 0.58 | 0.76 | 0.3843 | 0.9199 |
| A0A3Q1NA28 | *ALCAM* | activated leukocyte cell adhesion molecule | 3.83 | -0.48 | 0.75 | 0.3873 | 0.9234 |
| F1MBW3 | *ACSL4* | acyl-CoA synthetase long chain family member 4 | 5.27 | 0.41 | 0.74 | 0.3889 | 0.9234 |
| A0AAA9U0A7 | *SH3YL1* | SH3 and SYLF domain containing 1 | 2.77 | 0.68 | 0.74 | 0.3891 | 0.9234 |
| A0A3Q1LQB6 | *BCAS1* | brain enriched myelin associated protein 1 | 4.45 | 0.50 | 0.74 | 0.3899 | 0.9234 |
| P15497 | *APOA1* | NA | 11.86 | -0.50 | 0.74 | 0.3900 | 0.9234 |
| A0AAA9SCA4 | *LOC515570* | NA | 2.33 | -1.57 | 0.73 | 0.3924 | 0.9253 |
| Q0VCJ8 | *METTL9* | NA | 4.98 | -0.55 | 0.73 | 0.3925 | 0.9253 |
| A0AAA9SNQ5 | *OLA1* | Obg like ATPase 1 | 2.96 | 0.62 | 0.72 | 0.3953 | 0.9279 |
| A0A3Q1NLW9 | *VPS37B* | VPS37B subunit of ESCRT-I | 8.80 | -0.49 | 0.72 | 0.3960 | 0.9279 |
| P22226 | *CATHL1* | NA | 7.94 | 0.60 | 0.72 | 0.3966 | 0.9279 |
| E1BEV7 | *BMP1* | bone morphotic protein 1 | 3.79 | -0.75 | 0.72 | 0.3975 | 0.9279 |
| A7E3T1 | *CD151* | CD151 molecule | 4.72 | 0.61 | 0.71 | 0.3979 | 0.9279 |
| P68103 | *EEF1A1* | eukaryotic translation elongation factor 1 alpha 1 | 9.26 | 0.32 | 0.71 | 0.3996 | 0.9289 |
| A0AAA9STL0 | *TPP1* | tripeptidyl peptidase 1 | 2.95 | -0.85 | 0.71 | 0.4007 | 0.9289 |
| A0A3Q1M909 | *MSRA* | methionine sulfoxide reductase A | 2.42 | 0.70 | 0.70 | 0.4020 | 0.9289 |
| A0A3Q1MD47 | *SPSB1* | splA/ryanodine receptor domain and SOCS box containing 1 | 2.77 | -1.04 | 0.70 | 0.4021 | 0.9289 |
| Q58CY6 | *PRXL2B* | NA | 5.36 | 0.68 | 0.70 | 0.4026 | 0.9289 |
| Q2LGB5 | *TOLLIP* | NA | 6.46 | -0.38 | 0.70 | 0.4040 | 0.9290 |
| Q32KR9 | *CHMP1A* | charged multivesicular body protein 1A | 3.03 | -0.75 | 0.69 | 0.4053 | 0.9290 |
| P28801 | *GSTP1* | NA | 6.14 | 0.36 | 0.69 | 0.4056 | 0.9290 |
| P61085 | *UBE2K* | NA | 2.75 | 0.64 | 0.69 | 0.4061 | 0.9290 |
| P31408 | *ATP6V1B2* | ATPase H+ transporting V1 subunit B2 | 4.70 | 0.42 | 0.68 | 0.4083 | 0.9321 |
| A0AAA9SP21 | *SDCBP* | syndecan binding protein | 13.09 | -0.49 | 0.68 | 0.4097 | 0.9331 |
| P11151 | *LPL* | lipoprotein lipase | 6.54 | -0.56 | 0.68 | 0.4105 | 0.9331 |
| E1BGA2 | *MITD1* | microtubule interacting and trafficking domain containing 1 | 4.24 | -0.64 | 0.67 | 0.4119 | 0.9343 |
| P02769 | *ALB* | NA | 10.30 | -0.46 | 0.67 | 0.4138 | 0.9366 |
| A0AAA9TJ27 | *EMB* | embigin | 3.37 | -0.60 | 0.65 | 0.4184 | 0.9449 |
| F1MEX9 | *ACSL3* | acyl-CoA synthetase long chain family member 3 | 4.15 | 0.59 | 0.65 | 0.4197 | 0.9449 |
| E1BJ18 | *CAPN6* | calpain 6 | 8.79 | -0.50 | 0.64 | 0.4223 | 0.9449 |
| F1MDD6 | *KCTD14* | potassium channel tetramerization domain containing 14 | 3.00 | 0.57 | 0.64 | 0.4229 | 0.9449 |
| Q6QRN6 | *NAXE* | NA | 3.46 | -0.54 | 0.64 | 0.4233 | 0.9449 |
| Q08DR7 | *SNF8* | SNF8 subunit of ESCRT-II | 5.01 | -0.50 | 0.64 | 0.4236 | 0.9449 |
| Q17R06 | *RAB21* | RAB21, member RAS onco family | 5.83 | 0.30 | 0.63 | 0.4269 | 0.9449 |
| A7E3W4 | *TKT* | transketolase | 8.32 | 0.31 | 0.63 | 0.4279 | 0.9449 |
| A0AAA9TZQ7 | *SFT2D2* | SFT2 domain containing 2 | 4.82 | 0.36 | 0.63 | 0.4287 | 0.9449 |
| Q08DN8 | *FLOT1* | flotillin 1 | 7.51 | 0.34 | 0.63 | 0.4291 | 0.9449 |
| A0AAA9S835 | *ERAP1* | endoplasmic reticulum aminopeptidase 1 | 8.44 | -0.73 | 0.62 | 0.4293 | 0.9449 |
| Q56JW4 | *APRT* | NA | 6.84 | 0.29 | 0.62 | 0.4298 | 0.9449 |
| A0AAA9SS47 | *P3H1* | prolyl 3-hydroxylase 1 | 2.55 | -0.70 | 0.62 | 0.4298 | 0.9449 |
| A7MB62 | *ACTR2* | actin related protein 2 | 5.89 | 0.33 | 0.62 | 0.4303 | 0.9449 |
| Q5E956 | *TPI1* | NA | 6.92 | 0.30 | 0.62 | 0.4315 | 0.9449 |
| A0A452DI45 | *CAPZB* | NA | 6.77 | 0.29 | 0.61 | 0.4340 | 0.9449 |
| A0AAA9TG85 | *ATP13A4* | ATPase 13A4 | 7.69 | 0.54 | 0.61 | 0.4340 | 0.9449 |
| A0AAF7AFD7 | *EHD1* | NA | 7.26 | 0.39 | 0.61 | 0.4341 | 0.9449 |
| A0A3Q1M913 | *PFAS* | phosphoribosylformylglycinamidine synthase | 2.76 | 0.56 | 0.61 | 0.4342 | 0.9449 |
| Q58DC0 | *CPPED1* | NA | 6.53 | 0.29 | 0.61 | 0.4349 | 0.9449 |
| A0AAA9TU41 | *DCTN2* | dynactin subunit 2 | 2.53 | 1.36 | 0.60 | 0.4369 | 0.9469 |
| A0AAF6YJV2 | *GNAI1* | NA | 3.30 | 1.33 | 0.60 | 0.4378 | 0.9469 |
| Q92176 | *CORO1A* | coronin 1A | 2.27 | 1.37 | 0.60 | 0.4385 | 0.9469 |
| A0A3Q1LIB4 | *VCL* | vinculin | 5.89 | -0.30 | 0.59 | 0.4408 | 0.9469 |
| G3N0K3 | *NA* | NA | 2.62 | 0.59 | 0.59 | 0.4416 | 0.9469 |
| Q0VCK1 | *MYADM* | myeloid associated differentiation marker | 5.45 | 0.41 | 0.59 | 0.4431 | 0.9469 |
| A0AAA9SEV1 | *CYRIB* | CYFIP related Rac1 interactor B | 5.15 | 0.37 | 0.59 | 0.4438 | 0.9469 |
| A5D7A2 | *GARS1* | glycyl-tRNA synthetase 1 | 3.66 | -0.50 | 0.59 | 0.4442 | 0.9469 |
| E1BNE5 | *RAB11FIP5* | RAB11 family interacting protein 5 | 1.75 | 2.40 | 0.59 | 0.4443 | 0.9469 |
| A0AAA9SY97 | *UBE2D3* | ubiquitin conjugating enzyme E2 D3 | 5.36 | -0.35 | 0.58 | 0.4446 | 0.9469 |
| A0AAA9SQ31 | *TXNL1* | thioredoxin like 1 | 5.01 | 0.34 | 0.58 | 0.4469 | 0.9496 |
| P67774 | *PPP2CA* | protein phosphatase 2 catalytic subunit alpha | 2.85 | 0.57 | 0.58 | 0.4478 | 0.9496 |
| A0AAA9SRD3 | *CLIC6* | NA | 3.87 | 0.59 | 0.57 | 0.4489 | 0.9496 |
| Q28125 | *ICAM3* | NA | 4.83 | 0.53 | 0.57 | 0.4500 | 0.9496 |
| F6QQD9 | *NA* | NA | 2.68 | -1.39 | 0.57 | 0.4502 | 0.9496 |
| F1MHS5 | *S100A9* | S100 calcium binding protein A9 | 4.56 | 0.50 | 0.56 | 0.4556 | 0.9507 |
| Q5E946 | *PARK7* | Parkinsonism associated deglycase | 7.40 | 0.29 | 0.56 | 0.4558 | 0.9507 |
| A0AAF6DMH4 | *LANCL2* | LanC like glutathione S-transferase 2 | 2.71 | -1.39 | 0.56 | 0.4562 | 0.9507 |
| A0AAA9SD79 | *TMC5* | transmembrane channel like 5 | 5.36 | -0.72 | 0.55 | 0.4567 | 0.9507 |
| P61284 | *RPL12* | ribosomal protein L12 | 2.63 | 0.56 | 0.55 | 0.4574 | 0.9507 |
| Q3ZC42 | *ADH5* | alcohol dehydrogenase 5 (class III), chi polypeptide | 4.07 | 0.42 | 0.55 | 0.4580 | 0.9507 |
| A0A3Q1MKI2 | *NA* | NA | 8.36 | -0.85 | 0.55 | 0.4588 | 0.9507 |
| Q3T0D7 | *SAR1A* | secretion associated Ras related GTPase 1A | 6.57 | 0.30 | 0.55 | 0.4599 | 0.9507 |
| A0AAA9TLU8 | *AK2* | adenylate kinase 2 | 2.65 | -0.61 | 0.55 | 0.4603 | 0.9507 |
| F1MU85 | *BROX* | BRO1 domain and CAAX motif containing | 8.31 | -0.43 | 0.54 | 0.4605 | 0.9507 |
| Q3ZBT1 | *VCP* | NA | 5.35 | -0.29 | 0.54 | 0.4613 | 0.9507 |
| Q56JV6 | *TWF1* | NA | 4.72 | 0.39 | 0.54 | 0.4629 | 0.9507 |
| Q2T9M8 | *SNAP23* | synaptosome associated protein 23 | 8.88 | -0.27 | 0.54 | 0.4635 | 0.9507 |
| A0A3Q1M2V6 | *CTSZ* | cathepsin Z | 5.15 | -0.67 | 0.54 | 0.4638 | 0.9507 |
| A0AAA9TJ11 | *NQO1* | NAD(P)H quinone dehydrogenase 1 | 3.08 | 0.50 | 0.54 | 0.4644 | 0.9507 |
| A0AAA9T6F1 | *RTN4* | reticulon 4 | 2.66 | 1.23 | 0.53 | 0.4651 | 0.9507 |
| F1MU79 | *FKBP4* | FKBP prolyl isomerase 4 | 2.16 | 1.25 | 0.53 | 0.4679 | 0.9507 |
| A0AAA9SBV4 | *PTP4A2* | protein tyrosine phosphatase 4A2 | 5.29 | 0.32 | 0.53 | 0.4682 | 0.9507 |
| A0A3Q1NBQ9 | *CD82* | CD82 molecule | 8.22 | -0.51 | 0.52 | 0.4689 | 0.9507 |
| P11017 | *GNB2* | G protein subunit beta 2 | 9.28 | 0.28 | 0.52 | 0.4697 | 0.9507 |
| A5PKM1 | *ZNRF2* | zinc and ring finger 2 | 3.92 | 0.46 | 0.52 | 0.4704 | 0.9507 |
| A0A3Q1MC95 | *CRISPLD2* | cysteine rich secretory protein LCCL domain containing 2 | 5.81 | -0.37 | 0.52 | 0.4712 | 0.9507 |
| F1N7H0 | *DNAJC7* | DnaJ heat shock protein family (Hsp40) member C7 | 1.89 | 0.65 | 0.52 | 0.4715 | 0.9507 |
| Q5E9G3 | *PSME2* | proteasome activator subunit 2 | 5.21 | 0.37 | 0.52 | 0.4719 | 0.9507 |
| F1MFI4 | *CRTAC1* | cartilage acidic protein 1 | 7.59 | -0.69 | 0.51 | 0.4750 | 0.9545 |
| Q9MZ08 | *BCAM* | basal cell adhesion molecule | 3.93 | -0.42 | 0.51 | 0.4755 | 0.9545 |
| A0AAA9T211 | *PSAT1* | phosphoserine aminotransferase 1 | 4.13 | 0.44 | 0.51 | 0.4772 | 0.9562 |
| F1MNI4 | *RAB5B* | RAB5B, member RAS onco family | 5.88 | -0.26 | 0.50 | 0.4804 | 0.9576 |
| A0AAA9TJ69 | *APOE* | apolipoprotein E | 11.73 | -0.41 | 0.50 | 0.4808 | 0.9576 |
| A0A3Q1LSP7 | *APH1A* | aph-1 homolog A, gamma-secretase subunit | 4.75 | 0.45 | 0.50 | 0.4816 | 0.9576 |
| F1N4K7 | *FZD7* | frizzled class receptor 7 | 5.69 | -0.30 | 0.49 | 0.4838 | 0.9576 |
| A4IFN6 | *SNX18* | sorting nexin 18 | 5.40 | -0.44 | 0.49 | 0.4852 | 0.9576 |
| A0A3Q1M458 | *LOC104968484* | NA | 8.62 | -0.57 | 0.49 | 0.4860 | 0.9576 |
| A0AAA9TS64 | *IAH1* | isoamyl acetate hydrolyzing esterase 1 (putative) | 2.67 | 1.26 | 0.49 | 0.4861 | 0.9576 |
| Q9GL65 | *TLR4* | NA | 7.41 | -0.29 | 0.48 | 0.4869 | 0.9576 |
| Q5E947 | *PRDX1* | peroxiredoxin 1 | 7.65 | 0.28 | 0.48 | 0.4882 | 0.9576 |
| A0AAA9RU21 | *NA* | NA | 9.28 | -0.29 | 0.48 | 0.4892 | 0.9576 |
| Q27975 | *HSPA1A* | NA | 9.58 | -0.39 | 0.48 | 0.4892 | 0.9576 |
| P68252 | *YWHAG* | NA | 7.46 | 0.31 | 0.48 | 0.4895 | 0.9576 |
| F6RU06 | *FGG* | fibrinogen gamma chain | 10.41 | -0.56 | 0.48 | 0.4903 | 0.9576 |
| A0A3Q1MJK1 | *SERPINB6* | serpin peptidase inhibitor, clade B (ovalbumin), member 6 | 3.81 | 0.38 | 0.48 | 0.4903 | 0.9576 |
| F1N2K1 | *PCYOX1* | prenylcysteine oxidase 1 | 4.08 | -0.54 | 0.47 | 0.4928 | 0.9595 |
| A0AAA9SSZ3 | *ERLEC1* | endoplasmic reticulum lectin 1 | 2.20 | -0.67 | 0.47 | 0.4931 | 0.9595 |
| A0A3Q1ME19 | *CD2AP* | CD2 associated protein | 6.88 | 0.31 | 0.47 | 0.4948 | 0.9598 |
| Q3ZBG9 | *PLSCR2* | phospholipid scramblase 1 | 6.11 | -0.60 | 0.47 | 0.4953 | 0.9598 |
| A0AAA9SQM8 | *HSP90AB1* | heat shock protein 90 alpha family class B member 1 | 7.46 | 0.26 | 0.46 | 0.4966 | 0.9598 |
| F1N026 | *ITM2B* | integral membrane protein 2B | 8.72 | -0.35 | 0.46 | 0.4985 | 0.9598 |
| A0AAF6DMB5 | *ESYT1* | extended synaptotagmin 1 | 1.23 | 1.19 | 0.46 | 0.4994 | 0.9598 |
| A0A3Q1MJU0 | *RAB2A* | RAB2A, member RAS onco family | 6.33 | 0.24 | 0.46 | 0.4995 | 0.9598 |
| A0AAA9TRV4 | *SHISA5* | shisa family member 5 | 5.28 | -1.76 | 0.45 | 0.5000 | 0.9598 |
| F2Z4I1 | *RHEB* | Ras homolog, mTORC1 binding | 5.16 | 0.30 | 0.45 | 0.5003 | 0.9598 |
| G3MZ88 | *DNAJB9* | DnaJ heat shock protein family (Hsp40) member B9 | 3.85 | -0.52 | 0.45 | 0.5034 | 0.9636 |
| F1MJV6 | *ADGRG6* | adhesion G protein-coupled receptor G6 | 6.80 | -0.38 | 0.45 | 0.5041 | 0.9636 |
| A0A452DID6 | *DHRS11* | dehydrogenase/reductase 11 | 5.49 | -0.32 | 0.44 | 0.5066 | 0.9662 |
| P62833 | *RAP1A* | RAP1A, member of RAS onco family | 6.10 | -0.25 | 0.44 | 0.5072 | 0.9662 |
| A0A3Q1MI34 | *EPN1* | epsin 1 | 2.57 | 0.54 | 0.44 | 0.5083 | 0.9666 |
| P26452 | *RPSA* | ribosomal protein SA | 3.97 | 0.42 | 0.43 | 0.5101 | 0.9673 |
| A0AAA9RWP8 | *TMPRSS2* | transmembrane serine protease 2 | 4.83 | -0.35 | 0.43 | 0.5112 | 0.9673 |
| A0AAA9SIE7 | *NA* | NA | 4.36 | 0.43 | 0.43 | 0.5119 | 0.9673 |
| P22444 | *BMP3* | bone morphotic protein 3 | 2.11 | -1.22 | 0.43 | 0.5143 | 0.9673 |
| A0AAF6DM03 | *LRRC57* | leucine rich repeat containing 57 | 6.32 | 0.27 | 0.42 | 0.5153 | 0.9673 |
| A0AAA9RVP4 | *TMEM106B* | transmembrane protein 106B | 4.47 | -0.51 | 0.42 | 0.5153 | 0.9673 |
| A6QP36 | *LMAN2* | lectin, mannose binding 2 | 2.97 | -0.55 | 0.42 | 0.5156 | 0.9673 |
| F6R3N4 | *KRT10* | keratin 10 | 9.51 | -0.32 | 0.42 | 0.5159 | 0.9673 |
| A0A3Q1LXC8 | *EMC1* | ER membrane protein complex subunit 1 | 2.76 | -0.62 | 0.42 | 0.5167 | 0.9673 |
| A0A3Q1MM55 | *G6PD* | glucose-6-phosphate dehydrogenase | 4.80 | 0.38 | 0.41 | 0.5210 | 0.9736 |
| A0A3Q1M6K1 | *PTPRJ* | protein tyrosine phosphatase receptor type J | 7.54 | -0.34 | 0.41 | 0.5237 | 0.9766 |
| A0AAA9SKR8 | *NA* | NA | 6.96 | -0.45 | 0.40 | 0.5246 | 0.9766 |
| F1MUP1 | *FOLH1* | folate hydrolase 1 | 11.05 | -0.36 | 0.40 | 0.5253 | 0.9766 |
| Q32PA1 | *CD59* | CD59 molecule | 9.83 | -0.23 | 0.40 | 0.5288 | 0.9785 |
| Q5E9I4 | *MGAT1* | alpha-1,3-mannosyl-glycoprotein 2-beta-N-acetylglucosaminyltransferase | 8.88 | -0.41 | 0.40 | 0.5289 | 0.9785 |
| E1BK71 | *PACSIN3* | protein kinase C and casein kinase substrate in neurons 3 | 5.42 | 0.33 | 0.40 | 0.5290 | 0.9785 |
| Q3MHM5 | *TUBB4B* | tubulin beta 4B class IVb | 4.69 | -0.40 | 0.39 | 0.5304 | 0.9794 |
| A0AAA9TM29 | *BLVRB* | biliverdin reductase B | 4.23 | 0.31 | 0.39 | 0.5334 | 0.9817 |
| F1MFW9 | *KRT24* | keratin 24 | 5.47 | -0.44 | 0.39 | 0.5335 | 0.9817 |
| A0AAA9TXX7 | *GNAO1* | G protein subunit alpha o1 | 8.33 | 0.23 | 0.38 | 0.5383 | 0.9836 |
| A0AAA9SNV2 | *YARS1* | tyrosyl-tRNA synthetase 1 | 2.77 | 0.50 | 0.38 | 0.5386 | 0.9836 |
| Q17QT7 | *CYRIA* | CYFIP related Rac1 interactor A | 6.98 | 0.36 | 0.38 | 0.5389 | 0.9836 |
| A0AAA9TKA2 | *CLIC1* | chloride intracellular channel 1 | 6.83 | 0.29 | 0.38 | 0.5394 | 0.9836 |
| A0A3Q1LNG3 | *MFSD6* | major facilitator superfamily domain containing 6 | 1.40 | 1.06 | 0.37 | 0.5412 | 0.9836 |
| A0AAA9RTB6 | *YWHAE* | tyrosine 3-monooxygenase/tryptophan 5-monooxygenase activation protein epsilon | 10.67 | 0.23 | 0.37 | 0.5415 | 0.9836 |
| A0AAA9SNZ0 | *VPS37C* | VPS37C subunit of ESCRT-I | 4.33 | -0.50 | 0.37 | 0.5416 | 0.9836 |
| F1MS08 | *SLC12A2* | solute carrier family 12 member 2 | 5.65 | 0.39 | 0.37 | 0.5423 | 0.9836 |
| A0AAF6YUU7 | *NMRAL1* | NA | 0.96 | 1.08 | 0.37 | 0.5433 | 0.9836 |
| A0AAA9T5S8 | *ITGA6* | integrin subunit alpha 6 | 5.97 | 0.41 | 0.37 | 0.5439 | 0.9836 |
| A0AAA9T1Q1 | *BLVRA* | biliverdin reductase A | 4.10 | 0.47 | 0.37 | 0.5445 | 0.9836 |
| A0AAA9SUS5 | *ARF3* | ADP ribosylation factor 3 | 8.83 | -0.27 | 0.36 | 0.5463 | 0.9838 |
| A0AAA9T2J7 | *PITPNB* | phosphatidylinositol transfer protein beta | 5.44 | 0.25 | 0.36 | 0.5468 | 0.9838 |
| Q5E997 | *CAPZA2* | capping actin protein of muscle Z-line subunit alpha 2 | 7.29 | 0.23 | 0.36 | 0.5479 | 0.9838 |
| Q3SZF2 | *ARF4* | ADP ribosylation factor 4 | 6.47 | 0.29 | 0.36 | 0.5483 | 0.9838 |
| Q3MHR7 | *ARPC2* | actin related protein 2/3 complex subunit 2 | 6.45 | 0.27 | 0.35 | 0.5522 | 0.9844 |
| E1BJW2 | *STEAP4* | STEAP4 metalloreductase | 5.99 | 0.27 | 0.35 | 0.5537 | 0.9844 |
| A0AAA9TXF5 | *WBP2* | WW domain binding protein 2 | 1.86 | 1.00 | 0.35 | 0.5537 | 0.9844 |
| A0AAA9THQ0 | *EXOC7* | exocyst complex component 7 | -0.14 | 1.79 | 0.35 | 0.5541 | 0.9844 |
| Q5EAC2 | *GSS* | NA | 4.57 | 0.28 | 0.35 | 0.5552 | 0.9844 |
| A0AAA9TW15 | *FGB* | fibrinogen beta chain | 9.65 | -0.49 | 0.34 | 0.5574 | 0.9844 |
| Q5EAD0 | *BAIAP2* | BAR/IMD domain containing adaptor protein 2 | 7.41 | -0.23 | 0.34 | 0.5581 | 0.9844 |
| E1BBY7 | *HSPA4* | heat shock protein family A (Hsp70) member 4 | 2.50 | 0.48 | 0.34 | 0.5582 | 0.9844 |
| O18875 | *SLC6A8* | solute carrier family 6 member 8 | 7.43 | -0.25 | 0.34 | 0.5583 | 0.9844 |
| A0AAF6DLV1 | *RAB14* | RAB14, member RAS onco family | 5.55 | -0.29 | 0.34 | 0.5593 | 0.9844 |
| A0A3Q1MAZ8 | *ATP6V0A1* | ATPase H+ transporting V0 subunit a1 | 2.92 | 1.00 | 0.34 | 0.5601 | 0.9844 |
| A0AAA9U0A3 | *PTK7* | protein tyrosine kinase 7 (inactive) | 2.93 | -0.45 | 0.33 | 0.5638 | 0.9844 |
| A0AAF7A8X1 | *CAPG* | NA | 3.98 | 0.32 | 0.33 | 0.5643 | 0.9844 |
| Q2KJE7 | *PSME1* | proteasome activator subunit 1 | 5.91 | 0.28 | 0.33 | 0.5656 | 0.9844 |
| A0AAA9TXC4 | *PGLYRP2* | peptidoglycan recognition protein 2 | 2.65 | -0.50 | 0.33 | 0.5666 | 0.9844 |
| P68509 | *YWHAH* | tyrosine 3-monooxygenase/tryptophan 5-monooxygenase activation protein eta | 6.47 | 0.24 | 0.33 | 0.5675 | 0.9844 |
| A5D795 | *HRH1* | histamine receptor H1 | 1.29 | -1.40 | 0.32 | 0.5689 | 0.9844 |
| G5E5C8 | *TALDO1* | transaldolase 1 | 6.01 | 0.27 | 0.32 | 0.5694 | 0.9844 |
| A0AAA9RUQ7 | *ATIC* | 5-aminoimidazole-4-carboxamide ribonucleotide formyltransferase/IMP cyclohydrolase | 5.59 | 0.25 | 0.32 | 0.5701 | 0.9844 |
| E1BQ28 | *SLC39A8* | solute carrier family 39 member 8 | 4.29 | 0.57 | 0.32 | 0.5714 | 0.9844 |
| A6QLB1 | *RAB27A* | RAB27A, member RAS onco family | 2.28 | 0.95 | 0.32 | 0.5735 | 0.9844 |
| Q32PJ2 | *APOA4* | NA | 11.58 | -0.35 | 0.32 | 0.5745 | 0.9844 |
| A6H6Y1 | *BOLA-DQA1* | NA | 6.48 | 0.50 | 0.31 | 0.5756 | 0.9844 |
| E1BJ75 | *CRTAP* | cartilage associated protein | 5.71 | -0.41 | 0.31 | 0.5787 | 0.9844 |
| A0AAF7AEM3 | *SGTA* | NA | 2.57 | 0.94 | 0.30 | 0.5810 | 0.9844 |
| A0AAA9U0F6 | *SH3GL1* | SH3 domain containing GRB2 like 1, endophilin A2 | 5.38 | 0.33 | 0.30 | 0.5814 | 0.9844 |
| A0AAA9SUD5 | *LTF* | lactotransferrin | 13.52 | 0.53 | 0.30 | 0.5821 | 0.9844 |
| Q58CS7 | *CHMP3* | charged multivesicular body protein 3 | 6.11 | 0.33 | 0.30 | 0.5825 | 0.9844 |
| A0AAA9T9G7 | *PTGES3* | prostaglandin E synthase 3 | 2.84 | 0.39 | 0.30 | 0.5826 | 0.9844 |
| Q3T0N1 | *MVB12A* | multivesicular body subunit 12A | 7.82 | -0.35 | 0.30 | 0.5830 | 0.9844 |
| O46563 | *ATP6V1H* | ATPase H+ transporting V1 subunit H | 2.45 | 0.95 | 0.30 | 0.5848 | 0.9844 |
| Q3MI05 | *CTSA* | NA | 2.84 | -1.01 | 0.30 | 0.5858 | 0.9844 |
| A0A3Q1LUE9 | *NA* | NA | 11.97 | -0.64 | 0.30 | 0.5859 | 0.9844 |
| P13696 | *PEBP1* | NA | 7.94 | 0.23 | 0.29 | 0.5873 | 0.9844 |
| A0AAA9TKS9 | *PABPC1* | poly(A) binding protein cytoplasmic 1 | 0.94 | 2.03 | 0.29 | 0.5881 | 0.9844 |
| A0A3Q1MDG3 | *GK* | NA | 0.85 | 1.29 | 0.29 | 0.5893 | 0.9844 |
| E1BIX3 | *CAPN7* | calpain 7 | 7.62 | -0.31 | 0.29 | 0.5897 | 0.9844 |
| Q5BIP4 | *TOM1* | target of myb1 membrane trafficking protein | 5.40 | 0.26 | 0.29 | 0.5907 | 0.9844 |
| Q5E9F7 | *CFL1* | cofilin 1 | 9.42 | 0.22 | 0.29 | 0.5916 | 0.9844 |
| F1MZQ4 | *BTN1A1* | butyrophilin subfamily 1 member A1 | 16.42 | 0.22 | 0.28 | 0.5935 | 0.9844 |
| A0A3Q1N4Y8 | *JMJD8* | jumonji domain containing 8 | 4.98 | -0.45 | 0.28 | 0.5938 | 0.9844 |
| A0AAA9T9T5 | *NA* | NA | 5.35 | 0.29 | 0.28 | 0.5939 | 0.9844 |
| P05786 | *KRT8* | NA | 3.23 | -0.94 | 0.28 | 0.5956 | 0.9844 |
| F1MLZ1 | *CYBRD1* | cytochrome b reductase 1 | 9.40 | -0.45 | 0.28 | 0.5982 | 0.9844 |
| A0A3Q1MG97 | *LNPEP* | leucyl and cystinyl aminopeptidase | 1.79 | 0.91 | 0.28 | 0.5982 | 0.9844 |
| E1BNH2 | *CTH* | cystathionine gamma-lyase | 5.07 | -0.26 | 0.27 | 0.6004 | 0.9844 |
| Q3ZCK2 | *RALA* | RAS like proto-onco A | 7.46 | -0.22 | 0.27 | 0.6025 | 0.9844 |
| A0A3Q1MK61 | *UEVLD* | UEV and lactate/malate dehyrogenase domains | 8.31 | 0.29 | 0.27 | 0.6050 | 0.9844 |
| E1B7G3 | *STUB1* | STIP1 homology and U-box containing protein 1 | 1.84 | -1.24 | 0.27 | 0.6060 | 0.9844 |
| F6PZZ7 | *VPS4A* | vacuolar protein sorting 4 homolog A | 8.80 | -0.30 | 0.27 | 0.6064 | 0.9844 |
| Q32L99 | *PTGR2* | prostaglandin reductase 2 | 5.19 | 0.24 | 0.26 | 0.6082 | 0.9844 |
| A0AAF6Z333 | *CSNK1G1* | NA | 4.00 | 0.33 | 0.26 | 0.6091 | 0.9844 |
| G3N188 | *NA* | NA | 5.62 | -0.51 | 0.26 | 0.6105 | 0.9844 |
| Q2KIW4 | *LCAT* | lecithin-cholesterol acyltransferase | 5.41 | -0.42 | 0.26 | 0.6112 | 0.9844 |
| Q95122 | *CD14* | NA | 10.20 | -0.39 | 0.26 | 0.6113 | 0.9844 |
| F6QKI7 | *KRT5* | keratin 5 | 4.77 | -0.39 | 0.26 | 0.6114 | 0.9844 |
| Q2KI48 | *PTTG1IP* | PTTG1 interacting protein | 6.64 | -0.27 | 0.25 | 0.6140 | 0.9844 |
| Q58D08 | *PYCR3* | NA | 5.32 | 0.28 | 0.25 | 0.6140 | 0.9844 |
| A0AAA9T4B1 | *STK24* | serine/threonine kinase 24 | 3.68 | 0.30 | 0.25 | 0.6141 | 0.9844 |
| A0A3Q1LUP7 | *RAB1B* | RAB1B, member RAS onco family | 4.48 | -0.44 | 0.25 | 0.6146 | 0.9844 |
| A0A3Q1NHX4 | *FAH* | fumarylacetoacetate hydrolase | 2.55 | 0.91 | 0.25 | 0.6150 | 0.9844 |
| F1MZV2 | *CHMP5* | charged multivesicular body protein 5 | 7.02 | -0.33 | 0.25 | 0.6154 | 0.9844 |
| A4IFU2 | *RAP2B* | RAP2B, member of RAS onco family | 4.95 | -0.22 | 0.25 | 0.6164 | 0.9844 |
| A0A452DJG2 | *GNA11* | G protein subunit alpha 11 | 7.11 | 0.18 | 0.25 | 0.6169 | 0.9844 |
| A0AAA9TSW5 | *GOLGA7* | golgin A7 | 3.16 | -0.33 | 0.25 | 0.6176 | 0.9844 |
| A5D989 | *EEF1D* | NA | 1.90 | 0.87 | 0.25 | 0.6179 | 0.9844 |
| Q3T149 | *HSPB1* | heat shock protein family B (small) member 1 | 7.90 | 0.20 | 0.25 | 0.6180 | 0.9844 |
| Q58DW6 | *RAB25* | RAB25, member RAS onco family | 7.14 | 0.20 | 0.25 | 0.6185 | 0.9844 |
| Q2TA08 | *BAG5* | BAG cochaperone 5 | 2.73 | 0.85 | 0.25 | 0.6191 | 0.9844 |
| A0A3Q1LJG7 | *PMEPA1* | prostate transmembrane protein, androgen induced 1 | 4.08 | -0.47 | 0.25 | 0.6191 | 0.9844 |
| A0AAA9TES1 | *RAB5C* | RAB5C, member RAS onco family | 8.81 | 0.18 | 0.24 | 0.6233 | 0.9844 |
| A0AAF6DM44 | *CCNY* | cyclin Y | 2.34 | 0.83 | 0.24 | 0.6249 | 0.9844 |
| A0AAF7A9C9 | *CDC42* | NA | 8.30 | 0.23 | 0.24 | 0.6250 | 0.9844 |
| A0A3Q1LJ64 | *CTNNB1* | catenin beta 1 | 4.21 | 0.27 | 0.24 | 0.6259 | 0.9844 |
| Q08DV2 | *GNPNAT1* | glucosamine-phosphate N-acetyltransferase 1 | 2.40 | 0.82 | 0.24 | 0.6268 | 0.9844 |
| A0AAA9TNM9 | *SERINC3* | serine incorporator 3 | 3.91 | -0.35 | 0.23 | 0.6280 | 0.9844 |
| A0A3Q1MMV0 | *SH3GLB2* | SH3 domain containing GRB2 like, endophilin B2 | 2.52 | 0.41 | 0.23 | 0.6293 | 0.9844 |
| A0AAA9TDL4 | *MEMO1* | mediator of cell motility 1 | 3.28 | -0.40 | 0.23 | 0.6299 | 0.9844 |
| F1MNN6 | *MVP* | major vault protein | 5.01 | 0.33 | 0.23 | 0.6308 | 0.9844 |
| E1BA29 | *GNAQ* | G protein subunit alpha q | 9.10 | 0.17 | 0.23 | 0.6311 | 0.9844 |
| A0AAF6DLY7 | *PAK4* | p21 (RAC1) activated kinase 4 | 1.81 | 1.13 | 0.23 | 0.6313 | 0.9844 |
| O46631 | *SIRPA* | signal regulatory protein alpha | 5.72 | -0.28 | 0.23 | 0.6337 | 0.9844 |
| A0AAF6YWZ4 | *GDI1* | NA | 6.21 | 0.18 | 0.23 | 0.6339 | 0.9844 |
| A6H7F6 | *GOLPH3L* | NA | 2.23 | 0.38 | 0.23 | 0.6342 | 0.9844 |
| A0AAA9SUB4 | *ABI1* | abl interactor 1 | 4.36 | 0.27 | 0.23 | 0.6349 | 0.9844 |
| E1BLF1 | *PATJ* | PATJ crumbs cell polarity complex component | 5.34 | 0.29 | 0.22 | 0.6360 | 0.9844 |
| P68265 | *GLTP* | NA | 4.62 | 0.23 | 0.22 | 0.6360 | 0.9844 |
| A6H7G2 | *DBNL* | NA | 3.70 | 0.34 | 0.22 | 0.6378 | 0.9844 |
| A0AAF7AJ81 | *NA* | NA | 4.49 | -0.82 | 0.22 | 0.6387 | 0.9844 |
| A0AAF6YW73 | *NAXD* | NA | 3.40 | 0.34 | 0.22 | 0.6390 | 0.9844 |
| Q3T105 | *GALE* | NA | 2.60 | 0.79 | 0.22 | 0.6400 | 0.9844 |
| F1N6W6 | *SNX9* | sorting nexin 9 | 3.31 | 0.79 | 0.22 | 0.6411 | 0.9844 |
| Q3ZBG0 | *PSMA7* | proteasome 20S subunit alpha 7 | 5.12 | 0.21 | 0.21 | 0.6430 | 0.9844 |
| A0AAA9U1Y7 | *CD80* | CD80 molecule | 7.18 | -0.19 | 0.21 | 0.6439 | 0.9844 |
| A0AAA9RVD6 | *PCDHGB4* | protocadherin gamma subfamily B, 4 | 5.43 | -0.24 | 0.21 | 0.6445 | 0.9844 |
| A0AAA9T5Z8 | *NPEPPS* | aminopeptidase puromycin sensitive | 7.23 | 0.17 | 0.21 | 0.6447 | 0.9844 |
| A0A3Q1MS33 | *RDX* | radixin | 7.14 | 0.24 | 0.21 | 0.6448 | 0.9844 |
| A0AAA9TZ12 | *SHTN1* | shootin 1 | 2.14 | 0.78 | 0.21 | 0.6453 | 0.9844 |
| A1L528 | *RAB1A* | RAB1A, member RAS onco family | 8.44 | 0.18 | 0.21 | 0.6457 | 0.9844 |
| Q1RMW9 | *GOLPH3* | golgi phosphoprotein 3 | 2.96 | 0.32 | 0.21 | 0.6459 | 0.9844 |
| Q3ZCK3 | *BPNT1* | NA | 5.47 | 0.19 | 0.21 | 0.6465 | 0.9844 |
| A0AAF6YQA7 | *PSMA1* | NA | 4.72 | 0.24 | 0.21 | 0.6476 | 0.9844 |
| Q5E9T8 | *MVK* | NA | 0.00 | 1.69 | 0.21 | 0.6483 | 0.9844 |
| F1ML02 | *EPHB3* | EPH receptor B3 | 5.59 | -0.23 | 0.21 | 0.6493 | 0.9844 |
| E1BCU8 | *ICOSLG* | inducible T cell costimulator ligand | 5.20 | -0.23 | 0.21 | 0.6494 | 0.9844 |
| A0AAA9SX90 | *ENTREP1* | endosomal transmembrane epsin interactor 1 | 2.29 | -0.87 | 0.21 | 0.6495 | 0.9844 |
| A0A3Q1MPS2 | *SH3BP4* | SH3 domain binding protein 4 | 4.39 | 0.26 | 0.21 | 0.6503 | 0.9844 |
| A0AAF6YU11 | *PSMA6* | NA | 5.00 | -0.20 | 0.20 | 0.6520 | 0.9844 |
| F1MY82 | *PRKCH* | protein kinase C eta | 0.21 | 1.08 | 0.20 | 0.6525 | 0.9844 |
| B5TYW4 | *TLR1* | toll like receptor 1 | 2.25 | -0.39 | 0.20 | 0.6533 | 0.9844 |
| A0A3Q1MB37 | *ACSS2* | acyl-CoA synthetase short chain family member 2 | 5.42 | -0.20 | 0.20 | 0.6554 | 0.9844 |
| A0AAF6Z059 | *RHOC* | NA | 9.34 | -0.17 | 0.20 | 0.6561 | 0.9844 |
| A7E3Q2 | *HSPA2* | heat shock protein family A (Hsp70) member 2 | 4.23 | 0.29 | 0.20 | 0.6564 | 0.9844 |
| Q3ZCI9 | *CCT8* | chaperonin containing TCP1 subunit 8 | 3.31 | 0.31 | 0.20 | 0.6567 | 0.9844 |
| Q3ZBV8 | *TARS1* | threonyl-tRNA synthetase 1 | 5.85 | 0.18 | 0.20 | 0.6571 | 0.9844 |
| F1MUP9 | *VAT1* | vesicle amine transport 1 | 7.10 | -0.18 | 0.20 | 0.6578 | 0.9844 |
| A0AAA9TGJ5 | *NA* | NA | 3.84 | -0.25 | 0.19 | 0.6595 | 0.9844 |
| A0AAA9S8E2 | *PRSS23* | serine protease 23 | 4.30 | -0.33 | 0.19 | 0.6599 | 0.9844 |
| Q58DA7 | *GLRX3* | NA | 1.94 | 0.74 | 0.19 | 0.6649 | 0.9844 |
| A0AAF6Z0U5 | *EIF4A2* | NA | 3.09 | 0.31 | 0.19 | 0.6664 | 0.9844 |
| A0A3Q1M0I1 | *NA* | NA | 2.73 | 0.36 | 0.19 | 0.6666 | 0.9844 |
| A0AAA9TNM4 | *CPNE8* | copine 8 | 3.29 | -0.35 | 0.18 | 0.6680 | 0.9844 |
| A0AAA9TCP0 | *GP2* | glycoprotein 2 | 12.83 | -0.28 | 0.18 | 0.6693 | 0.9844 |
| A5D7R9 | *NRAS* | NRAS proto-onco, GTPase | 6.03 | -0.16 | 0.18 | 0.6709 | 0.9844 |
| Q1JP79 | *ARPC1A* | actin related protein 2/3 complex subunit 1A | 3.64 | 0.28 | 0.18 | 0.6717 | 0.9844 |
| A4FV32 | *PLEKHB2* | pleckstrin homology domain containing B2 | 4.99 | -0.27 | 0.18 | 0.6748 | 0.9844 |
| Q0IIG8 | *RAB18* | NA | 10.42 | 0.16 | 0.18 | 0.6750 | 0.9844 |
| A0A3Q1NM87 | *EPS15* | epidermal growth factor receptor pathway substrate 15 | 0.39 | 1.25 | 0.18 | 0.6751 | 0.9844 |
| Q3ZC35 | *CCN1* | cellular communication network factor 1 | 3.16 | -0.42 | 0.17 | 0.6774 | 0.9844 |
| A0A3Q1MHZ1 | *GBP1* | guanylate binding protein 1 | 2.95 | 0.30 | 0.17 | 0.6803 | 0.9844 |
| Q3T0U5 | *CHMP2A* | charged multivesicular body protein 2A | 8.01 | 0.49 | 0.17 | 0.6806 | 0.9844 |
| Q3ZCK9 | *PSMA4* | proteasome 20S subunit alpha 4 | 3.57 | 0.29 | 0.17 | 0.6808 | 0.9844 |
| Q1RMR9 | *PACSIN2* | protein kinase C and casein kinase substrate in neurons 2 | 5.66 | 0.22 | 0.17 | 0.6810 | 0.9844 |
| A0A3Q1LKM6 | *TFG* | trafficking from ER to golgi regulator | 3.40 | -1.28 | 0.17 | 0.6843 | 0.9844 |
| F1N5T9 | *SERINC5* | serine incorporator 5 | 6.09 | -0.46 | 0.16 | 0.6850 | 0.9844 |
| A0AAA9TN00 | *GMDS* | GDP-mannose 4,6-dehydratase | 2.61 | 0.29 | 0.16 | 0.6862 | 0.9844 |
| Q3SWW9 | *PPP1CB* | protein phosphatase 1 catalytic subunit beta | 2.08 | 0.68 | 0.16 | 0.6927 | 0.9844 |
| P61157 | *ACTR3* | NA | 6.77 | 0.18 | 0.16 | 0.6929 | 0.9844 |
| A0AAA9RRE8 | *LOC510369* | NA | 4.39 | 0.20 | 0.15 | 0.6959 | 0.9844 |
| E1BKQ2 | *RAB11FIP1* | RAB11 family interacting protein 1 | 1.28 | 0.95 | 0.15 | 0.6960 | 0.9844 |
| F1N7R5 | *CC2D1B* | coiled-coil and C2 domain containing 1B | 0.92 | 0.67 | 0.15 | 0.6979 | 0.9844 |
| A0A3Q1MMI3 | *MGRN1* | mahogunin ring finger 1 | 2.48 | -0.72 | 0.15 | 0.6990 | 0.9844 |
| Q08DI5 | *RAP2C* | RAP2C, member of RAS onco family | 6.16 | 0.17 | 0.15 | 0.6998 | 0.9844 |
| A0AAA9T600 | *NA* | NA | 3.25 | -0.40 | 0.15 | 0.7001 | 0.9844 |
| A0A3Q1M2P8 | *EPS8* | EGFR pathway substrate 8, signaling adaptor | 3.37 | -0.34 | 0.15 | 0.7007 | 0.9844 |
| F6PWS3 | *RALB* | RAS like proto-onco B | 6.26 | -0.15 | 0.15 | 0.7017 | 0.9844 |
| Q95M18 | *HSP90B1* | heat shock protein 90 beta family member 1 | 5.15 | -0.29 | 0.14 | 0.7035 | 0.9844 |
| E1BEN4 | *GMPPA* | GDP-mannose pyrophosphorylase A | 4.23 | 0.24 | 0.14 | 0.7037 | 0.9844 |
| O46414 | *FTH1* | ferritin heavy chain 1 | 5.22 | -0.24 | 0.14 | 0.7045 | 0.9844 |
| P31754 | *UMPS* | NA | 3.12 | -0.35 | 0.14 | 0.7058 | 0.9844 |
| Q2TBU0 | *HP* | haptoglobin | 7.93 | 0.28 | 0.14 | 0.7069 | 0.9844 |
| P01888 | *B2M* | beta-2-microglobulin | 9.23 | -0.46 | 0.14 | 0.7076 | 0.9844 |
| Q9N2J2 | *GPX4* | glutathione peroxidase 4 | 3.26 | 0.27 | 0.14 | 0.7078 | 0.9844 |
| A0A3Q1M762 | *NA* | NA | 11.50 | -0.15 | 0.14 | 0.7096 | 0.9844 |
| A0A3Q1LUG9 | *TXNDC5* | thioredoxin domain containing 5 | 5.47 | -0.27 | 0.14 | 0.7102 | 0.9844 |
| Q6QRN7 | *TMBIM1* | transmembrane BAX inhibitor motif containing 1 | 9.45 | -0.19 | 0.14 | 0.7108 | 0.9844 |
| A0AAA9RYW7 | *SPART* | spartin | 4.62 | 0.29 | 0.14 | 0.7109 | 0.9844 |
| A0A452DJ12 | *FCGR2B* | Fc gamma receptor IIb | 5.66 | 0.29 | 0.14 | 0.7110 | 0.9844 |
| F1MXD2 | *CDCP1* | CUB domain containing protein 1 | 1.90 | -0.64 | 0.14 | 0.7112 | 0.9844 |
| F1MD24 | *SLC5A1* | solute carrier family 5 member 1 | 8.97 | 0.15 | 0.14 | 0.7123 | 0.9844 |
| F1N290 | *COBLL1* | cordon-bleu WH2 repeat protein like 1 | 3.68 | 0.24 | 0.14 | 0.7125 | 0.9844 |
| A0AAA9T4C9 | *SLC44A1* | solute carrier family 44 member 1 | 3.19 | 0.25 | 0.13 | 0.7140 | 0.9844 |
| A0A3Q1LVV7 | *FGA* | fibrinogen alpha chain | 9.55 | -0.30 | 0.13 | 0.7157 | 0.9844 |
| A0AAF6DM78 | *ENG* | endoglin | 6.61 | 0.14 | 0.13 | 0.7170 | 0.9844 |
| A0A3Q1MHX8 | *RAB2A* | RAB2A, member RAS onco family | 8.30 | 0.14 | 0.13 | 0.7174 | 0.9844 |
| G3X743 | *RNPEP* | arginyl aminopeptidase | 2.84 | 0.25 | 0.13 | 0.7178 | 0.9844 |
| A5PJY9 | *CPNE3* | copine 3 | 5.97 | 0.15 | 0.13 | 0.7179 | 0.9844 |
| A0AAA9SWX5 | *CRB3* | crumbs cell polarity complex component 3 | 5.06 | 0.33 | 0.13 | 0.7179 | 0.9844 |
| Q762I5 | *RETN* | resistin | 5.62 | -0.35 | 0.13 | 0.7188 | 0.9844 |
| A0AAA9SBL4 | *AEBP1* | AE binding protein 1 | 4.50 | -0.66 | 0.13 | 0.7197 | 0.9844 |
| F6QAZ2 | *PCDHGB4* | protocadherin gamma subfamily B, 4 | 5.34 | -0.23 | 0.13 | 0.7198 | 0.9844 |
| A0AAF7ALE0 | *RPL18* | NA | 0.47 | 1.06 | 0.13 | 0.7210 | 0.9844 |
| A0AAA9U0Z7 | *NOS1AP* | nitric oxide synthase 1 adaptor protein | 2.01 | 0.62 | 0.13 | 0.7219 | 0.9844 |
| A7MB21 | *FERMT2* | FERM domain containing kindlin 2 | 2.60 | 0.26 | 0.13 | 0.7219 | 0.9844 |
| Q3SX42 | *CHMP2B* | NA | 4.82 | 0.64 | 0.13 | 0.7232 | 0.9844 |
| A0AAA9TDX2 | *SPPL2B* | signal peptide peptidase like 2B | 2.50 | -0.61 | 0.13 | 0.7235 | 0.9844 |
| A0AAA9SQI1 | *GFPT1* | glutamine--fructose-6-phosphate transaminase 1 | 3.86 | 0.22 | 0.12 | 0.7245 | 0.9844 |
| A0A3Q1MAQ8 | *PRSS8* | serine protease 8 | 7.78 | 0.15 | 0.12 | 0.7256 | 0.9844 |
| P02584 | *PFN1* | profilin 1 | 7.43 | -0.14 | 0.12 | 0.7268 | 0.9844 |
| Q1JPB0 | *SERPINB1* | serpin family B member 1 | 7.87 | 0.16 | 0.12 | 0.7278 | 0.9844 |
| P81019 | *NA* | NA | 1.45 | -0.60 | 0.12 | 0.7278 | 0.9844 |
| A0A3Q1MTH2 | *PPP3CA* | protein phosphatase 3 catalytic subunit alpha | 2.38 | 0.27 | 0.12 | 0.7287 | 0.9844 |
| P53712 | *ITGB1* | NA | 5.11 | 0.17 | 0.12 | 0.7293 | 0.9844 |
| F1MPZ9 | *NCKAP1* | NCK associated protein 1 | 2.68 | 0.33 | 0.12 | 0.7302 | 0.9844 |
| A0AAA9SF14 | *CLTC* | clathrin heavy chain | 3.04 | 0.30 | 0.12 | 0.7304 | 0.9844 |
| A0A452DK44 | *AZGP1* | alpha-2-glycoprotein 1, zinc-binding | 1.69 | -0.61 | 0.12 | 0.7312 | 0.9844 |
| A0AAA9TQX8 | *RPS3* | ribosomal protein S3 | 4.17 | 0.23 | 0.12 | 0.7322 | 0.9844 |
| A0AAF6YY22 | *UBTD1* | NA | 4.69 | -0.24 | 0.12 | 0.7322 | 0.9844 |
| A0AAF6Z676 | *IMPDH2* | NA | 2.49 | 0.57 | 0.12 | 0.7344 | 0.9844 |
| A0A3Q1LWU8 | *GPD1L* | glycerol-3-phosphate dehydrogenase 1 like | 3.49 | 0.21 | 0.11 | 0.7353 | 0.9844 |
| Q3T145 | *MDH1* | malate dehydrogenase 1 | 6.67 | 0.14 | 0.11 | 0.7354 | 0.9844 |
| O77834 | *PRDX6* | peroxiredoxin 6 | 7.59 | 0.12 | 0.11 | 0.7360 | 0.9844 |
| Q3SZ62 | *PGAM1* | phosphoglycerate mutase 1 | 6.23 | 0.15 | 0.11 | 0.7378 | 0.9844 |
| Q32L48 | *H2BC15* | NA | 5.71 | 0.20 | 0.11 | 0.7382 | 0.9844 |
| Q2TBH2 | *RRAS* | RAS related | 6.57 | 0.13 | 0.11 | 0.7385 | 0.9844 |
| Q3MHL7 | *CCT6A* | chaperonin containing TCP1 subunit 6A | 3.47 | 0.55 | 0.11 | 0.7414 | 0.9844 |
| Q0VCM0 | *S100A16* | S100 calcium binding protein A16 | 2.53 | -0.31 | 0.11 | 0.7415 | 0.9844 |
| F6RM11 | *NPTN* | neuroplastin | 4.48 | 0.17 | 0.11 | 0.7421 | 0.9844 |
| A0A3Q1M5A2 | *SRI* | sorcin | 7.02 | 0.17 | 0.11 | 0.7427 | 0.9844 |
| Q3ZBV6 | *LOC777594* | NA | 3.36 | -0.58 | 0.11 | 0.7428 | 0.9844 |
| A0AAA9SVV9 | *PTPA* | protein phosphatase 2 phosphatase activator | 2.55 | 0.55 | 0.11 | 0.7429 | 0.9844 |
| P61420 | *ATP6V0D1* | NA | 4.66 | 0.16 | 0.11 | 0.7441 | 0.9844 |
| A5D7H9 | *SLC6A14* | solute carrier family 6 member 14 | 9.38 | -0.12 | 0.11 | 0.7442 | 0.9844 |
| P61585 | *RHOA* | ras homolog family member A | 7.81 | 0.13 | 0.11 | 0.7445 | 0.9844 |
| P60661 | *MYL6* | myosin light chain 6 | 4.55 | 0.17 | 0.11 | 0.7446 | 0.9844 |
| F1N7I6 | *GCA* | grancalcin | 5.03 | -0.19 | 0.10 | 0.7460 | 0.9844 |
| P31404 | *ATP6V1A* | NA | 4.89 | 0.15 | 0.10 | 0.7464 | 0.9844 |
| Q58DU5 | *PSMA3* | proteasome 20S subunit alpha 3 | 4.16 | 0.21 | 0.10 | 0.7471 | 0.9844 |
| Q3ZBV1 | *IST1* | NA | 10.35 | 0.21 | 0.10 | 0.7480 | 0.9844 |
| A0AAF6YKW8 | *ESD* | NA | 3.70 | 0.19 | 0.10 | 0.7484 | 0.9844 |
| A0AAA9SYX9 | *UBE2N* | ubiquitin conjugating enzyme E2 N | 5.65 | 0.16 | 0.10 | 0.7507 | 0.9844 |
| A0AAF7AAG5 | *ALPL* | NA | 5.46 | 0.20 | 0.10 | 0.7508 | 0.9844 |
| A0AAA9SWE4 | *ANXA11* | annexin A11 | 8.84 | -0.13 | 0.10 | 0.7518 | 0.9844 |
| A0A3Q1MNQ1 | *TUT1* | terminal uridylyl transferase 1, U6 snRNA-specific | 2.24 | 0.25 | 0.10 | 0.7530 | 0.9844 |
| A0A452DIU9 | *SEC23B* | SEC23 homolog B, COPII coat complex component | 2.31 | 0.55 | 0.10 | 0.7543 | 0.9844 |
| A0A3Q1NMD6 | *NA* | NA | 2.72 | -0.28 | 0.10 | 0.7545 | 0.9844 |
| A0A3Q1NEI6 | *EIF4E* | eukaryotic translation initiation factor 4E | 1.65 | 0.53 | 0.10 | 0.7552 | 0.9844 |
| F1MGR1 | *SLC28A3* | solute carrier family 28 member 3 | 12.67 | 0.12 | 0.10 | 0.7553 | 0.9844 |
| A0A3Q1N802 | *RASAL1* | RAS protein activator like 1 | 5.58 | 0.12 | 0.10 | 0.7558 | 0.9844 |
| P28782 | *S100A8* | NA | 3.01 | 0.33 | 0.10 | 0.7575 | 0.9844 |
| Q2KIP5 | *PDZK1IP1* | NA | 4.50 | 0.17 | 0.09 | 0.7579 | 0.9844 |
| E1B9L0 | *FCGR2B* | Fc gamma receptor IIb | 12.84 | -0.17 | 0.09 | 0.7584 | 0.9844 |
| Q3SZI4 | *YWHAQ* | tyrosine 3-monooxygenase/tryptophan 5-monooxygenase activation protein theta | 8.66 | 0.12 | 0.09 | 0.7585 | 0.9844 |
| A0A3Q1MA33 | *NCAM2* | neural cell adhesion molecule 2 | 4.42 | -0.23 | 0.09 | 0.7588 | 0.9844 |
| A0A3Q1MEI7 | *PSEN1* | presenilin 1 | 4.85 | -0.13 | 0.09 | 0.7598 | 0.9844 |
| A0A3Q1MEL2 | *NA* | NA | 3.78 | 0.17 | 0.09 | 0.7600 | 0.9844 |
| A0A3Q1M3I6 | *PPP2R2A* | protein phosphatase 2 regulatory subunit Balpha | 0.29 | 0.53 | 0.09 | 0.7601 | 0.9844 |
| Q3T0D5 | *RPL30* | ribosomal protein L30 | 2.33 | -0.29 | 0.09 | 0.7604 | 0.9844 |
| A0A3Q1MM35 | *NA* | NA | 3.40 | -0.54 | 0.09 | 0.7610 | 0.9844 |
| A0AAF6Z6Y5 | *CLDN7* | NA | 4.80 | -0.22 | 0.09 | 0.7614 | 0.9844 |
| Q0P5B0 | *ARRDC1* | arrestin domain containing 1 | 8.35 | -0.15 | 0.09 | 0.7617 | 0.9844 |
| O97764 | *CRYZ* | crystallin zeta | 5.46 | -0.13 | 0.09 | 0.7619 | 0.9844 |
| A0A3Q1M0D7 | *VTA1* | vesicle trafficking 1 | 7.90 | -0.16 | 0.09 | 0.7621 | 0.9844 |
| A0A3Q1MR39 | *FLOT2* | flotillin 2 | 5.97 | -0.12 | 0.09 | 0.7634 | 0.9844 |
| Q3T0T1 | *PSMB10* | proteasome 20S subunit beta 10 | 3.37 | 0.20 | 0.09 | 0.7650 | 0.9844 |
| A0A452DIX8 | *CMPK1* | cytidine/uridine monophosphate kinase 1 | 5.45 | -0.14 | 0.09 | 0.7662 | 0.9844 |
| Q148J6 | *ARPC4* | actin related protein 2/3 complex subunit 4 | 7.05 | 0.14 | 0.09 | 0.7663 | 0.9844 |
| P80311 | *PPIB* | peptidylprolyl isomerase B | 4.43 | -0.22 | 0.09 | 0.7668 | 0.9844 |
| A0AAA9TUV1 | *ATRN* | attractin | 3.78 | -0.51 | 0.09 | 0.7671 | 0.9844 |
| A0A3Q1M3K7 | *RAB7A* | RAB7A, member RAS onco family | 9.48 | 0.11 | 0.09 | 0.7693 | 0.9844 |
| A0AAA9TKS4 | *NSFL1C* | NSFL1 cofactor | 3.34 | 0.18 | 0.09 | 0.7694 | 0.9844 |
| Q3ZBD7 | *GPI* | NA | 6.24 | 0.12 | 0.08 | 0.7717 | 0.9844 |
| Q765N9 | *CLDN3* | claudin 3 | 7.73 | 0.20 | 0.08 | 0.7722 | 0.9844 |
| Q0VCX5 | *ETF1* | eukaryotic translation termination factor 1 | 2.76 | 0.48 | 0.08 | 0.7739 | 0.9844 |
| A0A3Q1M481 | *AP1B1* | adaptor related protein complex 1 subunit beta 1 | 2.82 | 0.21 | 0.08 | 0.7785 | 0.9844 |
| P52175 | *NME1-2* | NA | 4.02 | -0.19 | 0.08 | 0.7787 | 0.9844 |
| A1A4I4 | *PKN1* | NA | 2.07 | 0.24 | 0.08 | 0.7816 | 0.9844 |
| P18892 | *BTN1A1* | butyrophilin subfamily 1 member A1 | 9.56 | 0.36 | 0.08 | 0.7831 | 0.9844 |
| A0A3Q1LIW3 | *AP2A2* | adaptor related protein complex 2 subunit alpha 2 | 4.81 | 0.20 | 0.08 | 0.7835 | 0.9844 |
| A7MBI9 | *SLC11A2* | solute carrier family 11 member 2 | 5.70 | 0.25 | 0.08 | 0.7838 | 0.9844 |
| A0AAA9TNL8 | *TMEM67* | transmembrane protein 67 | 2.32 | -0.66 | 0.08 | 0.7838 | 0.9844 |
| A0A3Q1N9V7 | *WASF2* | WASP family member 2 | 3.96 | 0.17 | 0.07 | 0.7876 | 0.9844 |
| A0A3Q1MU35 | *TBC1D24* | TBC1 domain family member 24 | 1.55 | 0.46 | 0.07 | 0.7893 | 0.9844 |
| A0AAA9SL89 | *CEMIP2* | cell migration inducing hyaluronidase 2 | 4.12 | 0.16 | 0.07 | 0.7928 | 0.9844 |
| A0AAA9U1I4 | *CYFIP2* | cytoplasmic FMR1 interacting protein 2 | 3.89 | 0.17 | 0.07 | 0.7955 | 0.9844 |
| Q3MHW6 | *SLC16A1* | solute carrier family 16 member 1 | 9.59 | 0.16 | 0.07 | 0.7977 | 0.9844 |
| Q29RR7 | *VPS37A* | VPS37A subunit of ESCRT-I | 2.34 | -0.45 | 0.06 | 0.7995 | 0.9844 |
| A0AAA9T4S7 | *CCT3* | chaperonin containing TCP1 subunit 3 | 2.92 | 0.20 | 0.06 | 0.8009 | 0.9844 |
| A0AAA9SIZ7 | *RFFL* | ring finger and FYVE like domain containing E3 ubiquitin protein ligase | 5.15 | -0.15 | 0.06 | 0.8010 | 0.9844 |
| F1N0J3 | *ATP11A* | ATPase phospholipid transporting 11A | 5.42 | -0.10 | 0.06 | 0.8050 | 0.9844 |
| A0AAF6YM87 | *NUDC* | NA | 2.91 | -0.45 | 0.06 | 0.8052 | 0.9844 |
| A5D7L5 | *SLC39A14* | NA | 2.71 | -0.45 | 0.06 | 0.8055 | 0.9844 |
| F1MLA4 | *BDH2* | 3-hydroxybutyrate dehydrogenase 2 | 4.16 | 0.13 | 0.06 | 0.8065 | 0.9844 |
| E1BG26 | *ATP13A3* | ATPase 13A3 | 3.49 | 0.22 | 0.06 | 0.8080 | 0.9844 |
| P19120 | *HSPA8* | NA | 13.09 | 0.11 | 0.06 | 0.8082 | 0.9844 |
| A0AAA9SPT2 | *HRG* | histidine rich glycoprotein | 5.94 | 0.15 | 0.06 | 0.8083 | 0.9844 |
| A0A3Q1LRX3 | *SIRPA* | signal regulatory protein alpha | 3.82 | -0.59 | 0.06 | 0.8090 | 0.9844 |
| A0AAA9SA40 | *GK5* | glycerol kinase 5 | 2.78 | 0.18 | 0.06 | 0.8091 | 0.9844 |
| A0A3Q1M4H2 | *ADAM9* | ADAM metallopeptidase domain 9 | 4.60 | 0.13 | 0.06 | 0.8093 | 0.9844 |
| A0A3Q1LXR2 | *RAC1* | Rac family small GTPase 1 | 9.99 | 0.11 | 0.06 | 0.8117 | 0.9844 |
| Q3T0L5 | *EPCAM* | NA | 7.40 | 0.15 | 0.06 | 0.8128 | 0.9844 |
| F6RR15 | *LSR* | lipolysis stimulated lipoprotein receptor | 3.43 | -0.42 | 0.06 | 0.8135 | 0.9844 |
| E1BPI3 | *FRK* | fyn related Src family tyrosine kinase | 4.03 | -0.17 | 0.06 | 0.8145 | 0.9844 |
| Q58DK5 | *ALAD* | aminolevulinate dehydratase | 3.60 | 0.14 | 0.05 | 0.8151 | 0.9844 |
| F6QEU6 | *PGM2* | phosphoglucomutase 2 | 6.57 | -0.09 | 0.05 | 0.8152 | 0.9844 |
| A6H7I5 | *DNM2* | NA | 3.32 | -0.18 | 0.05 | 0.8154 | 0.9844 |
| A0A3Q1MRU9 | *ILDR1* | immunoglobulin like domain containing receptor 1 | 3.71 | 0.15 | 0.05 | 0.8165 | 0.9844 |
| E1BG25 | *MELTF* | melanotransferrin | 6.59 | -0.22 | 0.05 | 0.8169 | 0.9844 |
| Q5E994 | *CHMP1B* | NA | 7.29 | 0.42 | 0.05 | 0.8172 | 0.9844 |
| P68401 | *PAFAH1B2P68402* | NA | 2.15 | 0.39 | 0.05 | 0.8178 | 0.9844 |
| Q1RMX7 | *NANS* | N-acetylneuraminate synthase | 3.88 | 0.14 | 0.05 | 0.8185 | 0.9844 |
| A0AAA9RWU4 | *NA* | NA | 2.16 | 0.21 | 0.05 | 0.8193 | 0.9844 |
| A0AAF6YQI1 | *FDPS* | NA | 2.33 | 0.18 | 0.05 | 0.8203 | 0.9844 |
| Q2HJG5 | *VPS35* | VPS35 retromer complex component | 2.29 | 0.40 | 0.05 | 0.8211 | 0.9844 |
| Q3MHR3 | *DYNLL2* | dynein light chain LC8-type 2 | 4.51 | -0.10 | 0.05 | 0.8217 | 0.9844 |
| F1N332 | *ULK3* | unc-51 like kinase 3 | 3.65 | -0.18 | 0.05 | 0.8218 | 0.9844 |
| Q5E9K0 | *PSMB2* | proteasome 20S subunit beta 2 | 3.87 | 0.12 | 0.05 | 0.8221 | 0.9844 |
| Q58DG6 | *FBXL20* | F-box and leucine rich repeat protein 20 | 4.80 | 0.12 | 0.05 | 0.8238 | 0.9844 |
| Q9TU47 | *EIF6* | eukaryotic translation initiation factor 6 | 2.85 | 0.17 | 0.05 | 0.8243 | 0.9844 |
| Q2TA46 | *UBL3* | NA | 3.83 | -0.14 | 0.05 | 0.8250 | 0.9844 |
| F6QZ79 | *NA* | NA | 2.96 | 0.18 | 0.05 | 0.8251 | 0.9844 |
| A4IFM4 | *CLDN8* | claudin 8 | 6.90 | -0.09 | 0.05 | 0.8258 | 0.9844 |
| F6RFP6 | *BSG* | basigin | 9.69 | -0.11 | 0.05 | 0.8263 | 0.9844 |
| A0A3S5ZPB0 | *ALDOC* | aldolase, fructose-bisphosphate C | 9.10 | 0.09 | 0.05 | 0.8272 | 0.9844 |
| Q2KJH9 | *ALDH9A1* | NA | 6.00 | -0.10 | 0.05 | 0.8273 | 0.9844 |
| A0A3Q1LT19 | *NA* | NA | 8.48 | -0.13 | 0.05 | 0.8280 | 0.9844 |
| Q0V8S0 | *HGS* | NA | 3.25 | -0.17 | 0.05 | 0.8280 | 0.9844 |
| Q58DD3 | *DNAJB2* | DnaJ heat shock protein family (Hsp40) member B2 | 3.28 | -0.14 | 0.05 | 0.8288 | 0.9844 |
| A0AAA9TIS4 | *MSN* | moesin | 10.35 | 0.08 | 0.05 | 0.8290 | 0.9844 |
| A0A3Q1N2S6 | *PEF1* | penta-EF-hand domain containing 1 | 3.40 | -0.14 | 0.05 | 0.8297 | 0.9844 |
| A0AAF6DMC2 | *NPC1* | NPC intracellular cholesterol transporter 1 | 6.26 | 0.10 | 0.05 | 0.8309 | 0.9844 |
| Q0VD53 | *VPS26A* | VPS26, retromer complex component A | 4.39 | 0.12 | 0.04 | 0.8331 | 0.9844 |
| G3N072 | *UMAD1* | UBAP1-MVB12-associated (UMA) domain containing 1 | 0.16 | 0.76 | 0.04 | 0.8350 | 0.9844 |
| A0AAA9S4E1 | *ARHGAP1* | Rho GTPase activating protein 1 | 1.13 | 0.49 | 0.04 | 0.8354 | 0.9844 |
| Q0IIG7 | *RAB5A* | NA | 6.60 | 0.08 | 0.04 | 0.8364 | 0.9844 |
| A0A3S5ZPA0 | *EXT2* | exostosin glycosyltransferase 2 | -0.37 | -0.61 | 0.04 | 0.8364 | 0.9844 |
| A0AAA9TQM2 | *SNX33* | sorting nexin 33 | 4.13 | 0.35 | 0.04 | 0.8365 | 0.9844 |
| Q2KJD0 | *TUBB5* | NA | 7.26 | 0.09 | 0.04 | 0.8369 | 0.9844 |
| A2VDX2 | *BOLA-DQB* | major histocompatibility complex, class II, DQ beta | 3.94 | 0.50 | 0.04 | 0.8382 | 0.9844 |
| P62935 | *PPIA* | peptidylprolyl isomerase A | 9.31 | 0.08 | 0.04 | 0.8383 | 0.9844 |
| A0A3Q1MA07 | *SERPINB5* | serpin family B member 5 | 4.83 | 0.10 | 0.04 | 0.8387 | 0.9844 |
| Q76LV2 | *HSP90AA1* | NA | 8.60 | 0.08 | 0.04 | 0.8394 | 0.9844 |
| Q9BGI3 | *PRDX2* | NA | 5.77 | 0.09 | 0.04 | 0.8407 | 0.9844 |
| A0A452DI02 | *LYPLA1* | NA | 3.53 | 0.12 | 0.04 | 0.8409 | 0.9844 |
| Q32PI5 | *PPP2R1A* | NA | 2.44 | 0.17 | 0.04 | 0.8416 | 0.9844 |
| A0AAA9T2Y5 | *LDHA* | NA | 5.56 | 0.14 | 0.04 | 0.8421 | 0.9844 |
| A0A3Q1LLH4 | *CTNND1* | catenin delta 1 | 2.05 | 0.33 | 0.04 | 0.8436 | 0.9844 |
| P08728 | *KRT19* | NA | 5.72 | -0.09 | 0.04 | 0.8447 | 0.9844 |
| F6PZI3 | *SLC29A1* | solute carrier family 29 member 1 | 8.89 | -0.12 | 0.04 | 0.8458 | 0.9844 |
| P00442 | *SOD1* | NA | 5.99 | 0.11 | 0.04 | 0.8464 | 0.9844 |
| F1MBB7 | *TGM1* | transglutaminase 1 | 3.72 | 0.14 | 0.04 | 0.8468 | 0.9844 |
| A0A452DJE7 | *NA* | NA | 4.00 | 0.11 | 0.04 | 0.8468 | 0.9844 |
| A0AAA9SSI8 | *ARL15* | ADP ribosylation factor like GTPase 15 | 4.97 | 0.11 | 0.04 | 0.8491 | 0.9844 |
| F1MJP7 | *UAP1* | UDP-N-acetylglucosamine pyrophosphorylase 1 | 4.01 | 0.13 | 0.04 | 0.8496 | 0.9844 |
| A0A3Q1LJY8 | *CRB3* | crumbs cell polarity complex component 3 | 3.73 | 0.13 | 0.04 | 0.8496 | 0.9844 |
| F1MGC2 | *LOC112448555* | NA | 4.57 | -0.33 | 0.04 | 0.8509 | 0.9844 |
| A0A3Q1LPQ0 | *P4HB* | prolyl 4-hydroxylase subunit beta | 5.28 | -0.13 | 0.04 | 0.8510 | 0.9844 |
| Q5E954 | *DNAJA1* | DnaJ heat shock protein family (Hsp40) member A1 | 6.93 | 0.08 | 0.04 | 0.8510 | 0.9844 |
| A0A3Q1M903 | *MPO* | myeloperoxidase | 3.40 | 0.33 | 0.03 | 0.8527 | 0.9844 |
| A0AAA9TR35 | *ITCH* | itchy E3 ubiquitin protein ligase | 4.59 | 0.13 | 0.03 | 0.8542 | 0.9844 |
| Q5EA79 | *GALM* | galactose mutarotase | 8.58 | -0.07 | 0.03 | 0.8543 | 0.9844 |
| A0A3Q1LRM2 | *RNF130* | ring finger protein 130 | 1.49 | -0.32 | 0.03 | 0.8557 | 0.9844 |
| G3MWU7 | *TPRN* | taperin | -0.64 | 0.80 | 0.03 | 0.8564 | 0.9844 |
| A7YY28 | *ABHD14B* | abhydrolase domain containing 14B | 7.91 | 0.06 | 0.03 | 0.8568 | 0.9844 |
| A0A3Q1LY00 | *PALS1* | protein associated with LIN7 1, MAGUK p55 family member | 6.70 | 0.09 | 0.03 | 0.8569 | 0.9844 |
| A0AAA9TDP3 | *MAL2* | mal, T cell differentiation protein 2 | 5.88 | 0.07 | 0.03 | 0.8582 | 0.9844 |
| Q2T9X2 | *CCT4* | NA | 2.78 | 0.16 | 0.03 | 0.8584 | 0.9844 |
| P04272 | *ANXA2* | annexin A2 | 11.53 | -0.08 | 0.03 | 0.8590 | 0.9844 |
| Q08E32 | *CHMP4B* | charged multivesicular body protein 4B | 7.84 | -0.10 | 0.03 | 0.8604 | 0.9844 |
| A0A3Q1MEM9 | *MUC1* | mucin 1, cell surface associated | 10.01 | 0.12 | 0.03 | 0.8610 | 0.9844 |
| A0AAA9TCB8 | *HSPG2* | heparan sulfate proteoglycan 2 | 3.31 | 0.15 | 0.03 | 0.8614 | 0.9844 |
| F1MZ71 | *ADGRL2* | adhesion G protein-coupled receptor L2 | 5.25 | -0.11 | 0.03 | 0.8628 | 0.9844 |
| A0A3S5ZPC1 | *SYPL1* | synaptophysin like 1 | 4.24 | 0.09 | 0.03 | 0.8630 | 0.9844 |
| E1BDE3 | *CTDSP1* | CTD small phosphatase 1 | 3.35 | 0.13 | 0.03 | 0.8633 | 0.9844 |
| A1A4J1 | *PFKL* | phosphofructokinase, liver type | 4.97 | -0.09 | 0.03 | 0.8642 | 0.9844 |
| F1MHP4 | *RAB22A* | RAB22A, member RAS onco family | 4.67 | 0.08 | 0.03 | 0.8651 | 0.9844 |
| P48644 | *ALDH1A1* | aldehyde dehydrogenase 1 family member A1 | 6.94 | -0.12 | 0.03 | 0.8655 | 0.9844 |
| P00515 | *PRKAR2A* | NA | 4.67 | 0.08 | 0.03 | 0.8659 | 0.9844 |
| Q28046 | *SCIN* | NA | 3.99 | 0.30 | 0.03 | 0.8661 | 0.9844 |
| E1BK52 | *STK10* | NA | 3.46 | 0.11 | 0.03 | 0.8688 | 0.9848 |
| A0AAA9T640 | *ATP6V1E1* | ATPase H+ transporting V1 subunit E1 | 2.81 | 0.28 | 0.03 | 0.8698 | 0.9848 |
| Q0P5F3 | *LIN7C* | NA | 6.01 | 0.08 | 0.03 | 0.8713 | 0.9848 |
| Q3ZBL4 | *LZTFL1* | leucine zipper transcription factor like 1 | 2.15 | 0.12 | 0.03 | 0.8721 | 0.9848 |
| G3N0V2 | *KRT1* | keratin 1 | 9.62 | 0.08 | 0.03 | 0.8731 | 0.9848 |
| E1B9H5 | *TGFBR3* | transforming growth factor beta receptor 3 | 6.41 | -0.10 | 0.03 | 0.8738 | 0.9848 |
| A0AAA9S8V6 | *SLC22A23* | solute carrier family 22 member 23 | 6.36 | -0.07 | 0.02 | 0.8764 | 0.9848 |
| F1MD94 | *MATN3* | matrilin 3 | 5.75 | 0.09 | 0.02 | 0.8765 | 0.9848 |
| A0A3Q1LZ40 | *NA* | NA | 4.29 | -0.38 | 0.02 | 0.8765 | 0.9848 |
| A0A3Q1M130 | *SLC5A9* | solute carrier family 5 member 9 | 5.10 | -0.09 | 0.02 | 0.8772 | 0.9848 |
| G5E589 | *PSMB1* | proteasome 20S subunit beta 1 | 4.69 | -0.10 | 0.02 | 0.8787 | 0.9848 |
| F1N412 | *AACS* | acetoacetyl-CoA synthetase | 3.91 | 0.10 | 0.02 | 0.8807 | 0.9848 |
| A0AAF7ABE8 | *PAH* | NA | 2.18 | 0.16 | 0.02 | 0.8816 | 0.9848 |
| A0AAF6YW37 | *PYGL* | NA | 6.45 | -0.06 | 0.02 | 0.8836 | 0.9848 |
| A0AAA9SVD1 | *PDIA3* | protein disulfide isomerase family A member 3 | 5.73 | 0.10 | 0.02 | 0.8839 | 0.9848 |
| A0A3Q1MT60 | *MIF* | macrophage migration inhibitory factor | 5.90 | -0.06 | 0.02 | 0.8852 | 0.9848 |
| A0AAA9RZ54 | *CMTM7* | CKLF like MARVEL transmembrane domain containing 7 | 4.88 | -0.07 | 0.02 | 0.8858 | 0.9848 |
| P26201 | *CD36* | CD36 molecule (CD36 blood group) | 15.18 | -0.07 | 0.02 | 0.8859 | 0.9848 |
| F1MYQ3 | *RIPOR1* | RHO family interacting cell polarization regulator 1 | 2.45 | 0.14 | 0.02 | 0.8865 | 0.9848 |
| A0A3Q1NJ45 | *ZDHHC20* | zinc finger DHHC-type palmitoyltransferase 20 | 4.21 | 0.07 | 0.02 | 0.8883 | 0.9848 |
| Q29S21 | *KRT7* | NA | 4.41 | -0.06 | 0.02 | 0.8888 | 0.9848 |
| A0A3Q1N2F2 | *LOC107131247* | NA | 2.23 | -0.34 | 0.02 | 0.8895 | 0.9848 |
| A0A3Q1N552 | *LOC112441545* | NA | 2.16 | 0.13 | 0.02 | 0.8896 | 0.9848 |
| A1A4R1 | *H2AC20* | H2A clustered histone 20 | 5.05 | 0.08 | 0.02 | 0.8910 | 0.9848 |
| F1N6D4 | *SLC34A2* | solute carrier family 34 member 2 | 12.18 | 0.08 | 0.02 | 0.8912 | 0.9848 |
| P63009 | *AP2B1* | adaptor related protein complex 2 subunit beta 1 | 4.38 | 0.07 | 0.02 | 0.8914 | 0.9848 |
| A0A3Q1MDT7 | *LOC132344729* | NA | 6.11 | 0.09 | 0.02 | 0.8921 | 0.9848 |
| Q3T0Y8 | *VAMP8* | vesicle associated membrane protein 8 | 6.14 | -0.07 | 0.02 | 0.8935 | 0.9848 |
| A0AAF6YRV5 | *SLC1A4* | NA | 1.85 | 0.23 | 0.02 | 0.8937 | 0.9848 |
| Q3T035 | *ARPC3* | actin related protein 2/3 complex subunit 3 | 4.20 | -0.14 | 0.02 | 0.8938 | 0.9848 |
| P0CH28 | *UBC* | ubiquitin C | 12.74 | -0.07 | 0.02 | 0.8949 | 0.9850 |
| A0A3Q1ML89 | *LOC100299180* | NA | 6.31 | -0.08 | 0.02 | 0.8967 | 0.9856 |
| A0AAA9RUL8 | *MFGE8* | milk fat globule EGF and factor V/VIII domain containing | 17.11 | -0.06 | 0.02 | 0.8973 | 0.9856 |
| Q3ZBW5 | *RHOB* | ras homolog family member B | 2.03 | -0.22 | 0.02 | 0.8990 | 0.9857 |
| A0A3Q1LUZ7 | *RHPN2* | rhophilin Rho GTPase binding protein 2 | 2.14 | -0.22 | 0.02 | 0.8994 | 0.9857 |
| Q3ZCL0 | *CRISP3* | cysteine-rich secretory protein 3 | 2.96 | -0.39 | 0.02 | 0.9001 | 0.9857 |
| A0AAF6ZJE9 | *DPP4* | NA | 3.84 | 0.09 | 0.01 | 0.9026 | 0.9874 |
| G3MWP1 | *ELANE* | elastase, neutrophil expressed | 3.68 | 0.08 | 0.01 | 0.9036 | 0.9876 |
| Q3SZA1 | *RHOF* | ras homolog family member F, filopodia associated | 5.50 | 0.08 | 0.01 | 0.9048 | 0.9879 |
| A0AAA9SYC7 | *MTHFD1* | methylenetetrahydrofolate dehydrogenase, cyclohydrolase and formyltetrahydrofolate synthetase 1 | 3.56 | -0.08 | 0.01 | 0.9079 | 0.9903 |
| A0A3Q1M5R4 | *LDHB* | lactate dehydrogenase B | 9.25 | -0.05 | 0.01 | 0.9096 | 0.9905 |
| Q3T0Q2 | *TMEM59* | NA | 5.60 | -0.06 | 0.01 | 0.9104 | 0.9905 |
| A0AAA9RVE1 | *CRKL* | CRK like proto-oncogene, adaptor protein | 2.58 | -0.08 | 0.01 | 0.9113 | 0.9905 |
| G5E531 | *TCP1* | t-complex 1 | 2.17 | 0.19 | 0.01 | 0.9140 | 0.9905 |
| Q9XT56 | *F11R* | F11 receptor | 5.13 | -0.05 | 0.01 | 0.9146 | 0.9905 |
| A0AAA9TEG9 | *MFSD4A* | major facilitator superfamily domain containing 4A | 2.26 | -0.09 | 0.01 | 0.9154 | 0.9905 |
| A0AAA9S7I0 | *FH* | fumarate hydratase | 1.50 | -0.18 | 0.01 | 0.9181 | 0.9905 |
| Q95107 | *WASL* | WASP like actin nucleation promoting factor | 2.52 | 0.18 | 0.01 | 0.9189 | 0.9905 |
| E1BEN2 | *PRKACB* | protein kinase cAMP-activated catalytic subunit beta | 1.35 | 0.18 | 0.01 | 0.9194 | 0.9905 |
| E1BKM4 | *PDCD6IP* | programmed cell death 6 interacting protein | 12.23 | 0.05 | 0.01 | 0.9204 | 0.9905 |
| A0AAF6YHR8 | *ANXA4* | NA | 8.90 | -0.04 | 0.01 | 0.9208 | 0.9905 |
| F1MTR4 | *IGSF8* | immunoglobulin superfamily member 8 | 5.36 | 0.08 | 0.01 | 0.9209 | 0.9905 |
| A0AAA9SPQ2 | *ARFIP1* | ADP ribosylation factor interacting protein 1 | 3.66 | -0.06 | 0.01 | 0.9220 | 0.9905 |
| A0A3Q1MUN1 | *ANO6* | anoctamin 6 | 4.87 | 0.05 | 0.01 | 0.9227 | 0.9905 |
| F1MWQ2 | *ANXA3* | annexin A3 | 4.10 | 0.10 | 0.01 | 0.9231 | 0.9905 |
| Q3T0Q4 | *NME2* | NME/NM23 nucleoside diphosphate kinase 2 | 9.05 | -0.04 | 0.01 | 0.9231 | 0.9905 |
| A0A3Q1LRW6 | *NA* | NA | 5.16 | -0.09 | 0.01 | 0.9269 | 0.9905 |
| Q3SYX9 | *ARPC5* | NA | 4.47 | 0.04 | 0.01 | 0.9270 | 0.9905 |
| P00829 | *ATP5F1B* | NA | 5.30 | 0.07 | 0.01 | 0.9271 | 0.9905 |
| A0A3Q1M4F5 | *ANXA7* | annexin A7 | 8.15 | -0.04 | 0.01 | 0.9273 | 0.9905 |
| A0AAA9SDR6 | *SLC15A2* | solute carrier family 15 member 2 | 7.21 | 0.04 | 0.01 | 0.9273 | 0.9905 |
| F1MS05 | *ACO1* | aconitase 1 | 8.24 | 0.03 | 0.01 | 0.9287 | 0.9910 |
| A0AAA9SYV6 | *CALR* | calreticulin | 5.66 | -0.06 | 0.01 | 0.9308 | 0.9910 |
| A0A3Q1LVC7 | *EZR* | ezrin | 10.30 | -0.04 | 0.01 | 0.9309 | 0.9910 |
| A0A3Q1MT05 | *UGP2* | UDP-glucose pyrophosphorylase 2 | 7.20 | 0.04 | 0.01 | 0.9315 | 0.9910 |
| F1N261 | *LYN* | LYN proto-onco, Src family tyrosine kinase | 7.20 | -0.05 | 0.01 | 0.9329 | 0.9916 |
| A0AAA9RZL9 | *YES1* | YES proto-onco 1, Src family tyrosine kinase | 5.65 | 0.04 | 0.01 | 0.9345 | 0.9918 |
| Q5E9D5 | *DSTN* | destrin, actin depolymerizing factor | 5.32 | -0.03 | 0.01 | 0.9360 | 0.9918 |
| E1BFG0 | *ALDH7A1* | aldehyde dehydrogenase 7 family member A1 | 4.32 | -0.04 | 0.01 | 0.9362 | 0.9918 |
| A0A3Q1MFR4 | *APOB* | apolipoprotein B | 1.85 | 0.14 | 0.01 | 0.9368 | 0.9918 |
| P62157 | *CALM* | NA | 6.64 | 0.03 | 0.01 | 0.9385 | 0.9921 |
| F1MLI8 | *PODXL* | podocalyxin like | 7.39 | -0.04 | 0.01 | 0.9392 | 0.9921 |
| A0AAF6Z6R8 | *RACK1* | NA | 5.31 | 0.04 | 0.01 | 0.9399 | 0.9921 |
| Q3T114 | *RIDA* | reactive intermediate imine deaminase A homolog | 4.31 | -0.04 | 0.01 | 0.9421 | 0.9924 |
| A0AAA9SAG2 | *BAIAP2L1* | BAR/IMD domain containing adaptor protein 2 like 1 | 1.77 | 0.13 | 0.01 | 0.9422 | 0.9924 |
| F1MJ96 | *ARHGEF37* | Rho guanine nucleotide exchange factor 37 | 1.40 | 0.12 | 0.00 | 0.9441 | 0.9924 |
| G3X696 | *SLK* | STE20 like kinase | 2.36 | -0.12 | 0.00 | 0.9450 | 0.9924 |
| E1BI14 | *TECTB* | tectorin beta | 2.66 | -0.12 | 0.00 | 0.9473 | 0.9924 |
| F1N647 | *FASN* | fatty acid synthase | 7.12 | 0.04 | 0.00 | 0.9504 | 0.9924 |
| P33672 | *PSMB3* | proteasome 20S subunit beta 3 | 3.46 | -0.04 | 0.00 | 0.9509 | 0.9924 |
| A0A3Q1MFV1 | *CD36* | CD36 molecule (CD36 blood group) | 12.47 | 0.03 | 0.00 | 0.9515 | 0.9924 |
| A0AAF7AP66 | *LRRC8C* | NA | 3.36 | 0.04 | 0.00 | 0.9515 | 0.9924 |
| F1MMV6 | *CAB39L* | calcium binding protein 39 like | 3.17 | -0.04 | 0.00 | 0.9520 | 0.9924 |
| A0AAF7AGM7 | *TAGLN2* | NA | 4.28 | 0.03 | 0.00 | 0.9531 | 0.9924 |
| A0AAA9RU90 | *AZU1* | azurocidin 1 | 6.01 | -0.05 | 0.00 | 0.9534 | 0.9924 |
| P81947 | *NA* | NA | 7.39 | -0.02 | 0.00 | 0.9535 | 0.9924 |
| A0AAA9SNN4 | *PGK1* | phosphoglycerate kinase 1 | 7.05 | -0.02 | 0.00 | 0.9583 | 0.9924 |
| A0A3Q1MDC6 | *NCSTN* | nicastrin | 7.07 | -0.02 | 0.00 | 0.9584 | 0.9924 |
| A0AAA9TAG5 | *SELENBP1* | selenium binding protein 1 | 8.71 | 0.02 | 0.00 | 0.9588 | 0.9924 |
| G3N342 | *NA* | NA | 5.63 | -0.03 | 0.00 | 0.9593 | 0.9924 |
| F1MY58 | *TOM1L1* | target of myb1 like 1 membrane trafficking protein | 6.48 | 0.02 | 0.00 | 0.9598 | 0.9924 |
| A0AAA9TT41 | *ARF6* | ADP ribosylation factor 6 | 6.45 | 0.02 | 0.00 | 0.9599 | 0.9924 |
| A0A3Q1LWR9 | *UBE2V2* | ubiquitin conjugating enzyme E2 V2 | 5.11 | -0.02 | 0.00 | 0.9599 | 0.9924 |
| A0AAA9RWM9 | *DHRS3* | dehydrogenase/reductase 3 | 3.56 | 0.04 | 0.00 | 0.9601 | 0.9924 |
| A0AAA9SMR2 | *ECHDC1* | ethylmalonyl-CoA decarboxylase 1 | 8.06 | 0.02 | 0.00 | 0.9606 | 0.9924 |
| A5D7D1 | *ACTN4* | actinin alpha 4 | 6.67 | 0.02 | 0.00 | 0.9612 | 0.9924 |
| A0A3Q1MPS4 | *ACTN1* | actinin alpha 1 | 2.18 | 0.08 | 0.00 | 0.9657 | 0.9942 |
| A0A3Q1LSV1 | *NA* | NA | 8.05 | 0.02 | 0.00 | 0.9677 | 0.9942 |
| G5E641 | *NA* | NA | 5.79 | -0.03 | 0.00 | 0.9699 | 0.9942 |
| Q9GMB8 | *SARS1* | seryl-tRNA synthetase 1 | 5.04 | 0.02 | 0.00 | 0.9702 | 0.9942 |
| E1BCI3 | *SLC7A4* | solute carrier family 7 member 4 | 7.18 | 0.03 | 0.00 | 0.9705 | 0.9942 |
| A0AAA9TPT4 | *ANXA6* | annexin A6 | 3.62 | 0.06 | 0.00 | 0.9706 | 0.9942 |
| A0A3Q1NA59 | *CD47* | CD47 molecule | 9.12 | 0.01 | 0.00 | 0.9731 | 0.9942 |
| A0A3Q1M6J2 | *TSPAN15* | tetraspanin 15 | 2.95 | 0.03 | 0.00 | 0.9741 | 0.9942 |
| A0AAF6DLS8 | *RAB35* | RAB35, member RAS onco family | 6.36 | -0.01 | 0.00 | 0.9753 | 0.9942 |
| E1BGB1 | *VPS9D1* | VPS9 domain containing 1 | 1.56 | 0.07 | 0.00 | 0.9771 | 0.9942 |
| P81287 | *ANXA5* | NA | 11.90 | 0.01 | 0.00 | 0.9789 | 0.9942 |
| Q08E11 | *PPIC* | peptidylprolyl isomerase C | 4.84 | 0.02 | 0.00 | 0.9791 | 0.9942 |
| O97680 | *TXN* | NA | 6.20 | -0.01 | 0.00 | 0.9792 | 0.9942 |
| E1BQ32 | *CRK* | CRK proto-onco, adaptor protein | 3.97 | 0.02 | 0.00 | 0.9792 | 0.9942 |
| A0A3Q1MBS7 | *RAB6A* | RAB6A, member RAS onco family | 5.69 | 0.01 | 0.00 | 0.9799 | 0.9942 |
| Q0II59 | *PDXK* | pyridoxal kinase | 8.27 | 0.01 | 0.00 | 0.9812 | 0.9942 |
| A0AAF6ZJM9 | *QDPR* | NA | 4.05 | -0.01 | 0.00 | 0.9815 | 0.9942 |
| A0AAA9RZL3 | *SCAMP2* | secretory carrier membrane protein 2 | 5.32 | -0.01 | 0.00 | 0.9821 | 0.9942 |
| A0A3Q1MUU9 | *CCT7* | chaperonin containing TCP1 subunit 7 | 1.70 | 0.02 | 0.00 | 0.9824 | 0.9942 |
| A6QQQ3 | *PRELP* | proline and arginine rich end leucine rich repeat protein | 3.26 | -0.02 | 0.00 | 0.9828 | 0.9942 |
| F1MW13 | *DNASE1L1* | deoxyribonuclease 1 like 1 | 5.80 | 0.01 | 0.00 | 0.9831 | 0.9942 |
| F1MSN2 | *TACSTD2* | tumor associated calcium signal transducer 2 | 4.43 | -0.01 | 0.00 | 0.9831 | 0.9942 |
| Q3ZBT5 | *STX7* | syntaxin 7 | 4.14 | 0.01 | 0.00 | 0.9853 | 0.9955 |
| A0AAA9TID3 | *COTL1* | coactosin like F-actin binding protein 1 | 3.16 | -0.01 | 0.00 | 0.9883 | 0.9975 |
| A0AAF6YPV7 | *CHMP4A* | NA | 6.78 | 0.01 | 0.00 | 0.9895 | 0.9978 |
| A0A3Q1N4F3 | *GPHN* | gephyrin | 2.84 | -0.01 | 0.00 | 0.9913 | 0.9987 |
| G3X6I7 | *RP2* | RP2 activator of ARL3 GTPase | 5.40 | 0.00 | 0.00 | 0.9948 | 10.000 |
| Q0VD48 | *VPS4B* | vacuolar protein sorting 4 homolog B | 9.47 | 0.00 | 0.00 | 0.9955 | 10.000 |
| Q28024 | *GNG12* | G protein subunit gamma 12 | 4.53 | 0.00 | 0.00 | 0.9971 | 10.000 |
| E1BNC4 | *DIPK2A* | divergent protein kinase domain 2A | 1.62 | 0.01 | 0.00 | 0.9972 | 10.000 |
| A0A3Q1N6S6 | *PDLIM5* | PDZ and LIM domain 5 | 3.67 | 0.00 | 0.00 | 0.9980 | 10.000 |
| F6RS49 | *STAM* | signal transducing adaptor molecule | 2.11 | 0.00 | 0.00 | 0.9993 | 10.000 |
| E1BJQ7 | *GIPC1* | GIPC PDZ domain containing family member 1 | 5.71 | 0.00 | 0.00 | 0.9998 | 10.000 |
| Q5E958 | *RPS8* | NA | 2.88 | 0.69 | -10.38 | 10.000 | 10.000 |

**Supplementary Table 3:** Protein set encrichment analysis. ID = GO, Protein Class and Reactome identification; FDR = False Discovery Rate

| **Term Description (ID)** | **Number of genes** | **Over(+)/Under(-)** | **p-value** | **FDR** |
| --- | --- | --- | --- | --- |
| **GO Biological Process** | | | | |
| actin cytoskeleton organization (GO:0030036) | 37 | + | 1.78E-04 | 1.12E-02 |
| actin filament organization (GO:0007015) | 29 | + | 5.22E-04 | 2.09E-02 |
| actin filament-based process (GO:0030029) | 37 | + | 1.78E-04 | 1.07E-02 |
| activation of immune response (GO:0002253) | 18 | - | 2.57E-05 | 2.27E-03 |
| adaptive immune response (GO:0002250) | 11 | - | 3.04E-04 | 1.44E-02 |
| adaptive immune response based on somatic recombination of immune receptors built from immunoglobulin superfamily domains (GO:0002460) | 11 | - | 3.04E-04 | 1.61E-02 |
| amide biosynthetic process (GO:0043604) | 14 | + | 1.65E-03 | 4.97E-02 |
| aromatic compound catabolic process (GO:0019439) | 16 | + | 8.52E-04 | 2.81E-02 |
| cellular aromatic compound metabolic process (GO:0006725) | 52 | + | 6.13E-05 | 4.77E-03 |
| cellular nitrogen compound biosynthetic process (GO:0044271) | 27 | + | 2.71E-04 | 1.49E-02 |
| cellular nitrogen compound catabolic process (GO:0044270) | 14 | + | 1.52E-03 | 4.78E-02 |
| cellular nitrogen compound metabolic process (GO:0034641) | 58 | + | 5.80E-07 | 2.56E-04 |
| complement activation (GO:0006956) | 13 | - | 1.79E-06 | 5.90E-04 |
| cytoskeleton organization (GO:0007010) | 54 | + | 1.51E-04 | 9.96E-03 |
| exocytosis (GO:0006887) | 13 | + | 2.49E-05 | 2.35E-03 |
| export from cell (GO:0140352) | 15 | + | 2.13E-05 | 2.34E-03 |
| glycoprotein metabolic process (GO:0009100) | 15 | - | 5.46E-04 | 2.06E-02 |
| heterocycle catabolic process (GO:0046700) | 14 | + | 1.52E-03 | 4.67E-02 |
| heterocycle metabolic process (GO:0046483) | 55 | + | 4.21E-04 | 1.74E-02 |
| humoral immune response (GO:0006959) | 18 | - | 1.22E-05 | 1.80E-03 |
| immune effector process (GO:0002252) | 21 | - | 8.65E-09 | 1.14E-05 |
| immune response (GO:0006955) | 42 | - | 8.99E-09 | 5.94E-06 |
| immune system process (GO:0002376) | 52 | - | 2.58E-06 | 6.82E-04 |
| immunoglobulin mediated immune response (GO:0016064) | 11 | - | 3.04E-04 | 1.39E-02 |
| intracellular signal transduction (GO:0035556) | 22 | + | 2.33E-04 | 1.34E-02 |
| leukocyte mediated immunity (GO:0002443) | 11 | - | 3.04E-04 | 1.49E-02 |
| lymphocyte mediated immunity (GO:0002449) | 11 | - | 3.04E-04 | 1.55E-02 |
| nucleobase-containing compound metabolic process (GO:0006139) | 46 | + | 2.40E-05 | 2.44E-03 |
| nucleobase-containing small molecule metabolic process (GO:0055086) | 33 | + | 6.81E-04 | 2.43E-02 |
| nucleoside phosphate metabolic process (GO:0006753) | 27 | + | 6.55E-04 | 2.41E-02 |
| organelle organization (GO:0006996) | 83 | + | 1.47E-04 | 1.02E-02 |
| organic cyclic compound catabolic process (GO:1901361) | 16 | + | 8.52E-04 | 2.75E-02 |
| organic cyclic compound metabolic process (GO:1901360) | 60 | + | 3.63E-04 | 1.55E-02 |
| peptide biosynthetic process (GO:0043043) | 13 | + | 8.18E-04 | 2.77E-02 |
| positive regulation of immune response (GO:0050778) | 21 | - | 9.92E-06 | 1.64E-03 |
| positive regulation of immune system process (GO:0002684) | 26 | - | 5.10E-06 | 1.12E-03 |
| positive regulation of response to stimulus (GO:0048584) | 28 | - | 1.40E-05 | 1.85E-03 |
| regulation of immune response (GO:0050776) | 23 | - | 1.61E-05 | 1.94E-03 |
| regulation of immune system process (GO:0002682) | 29 | - | 6.22E-06 | 1.18E-03 |
| regulation of response to stimulus (GO:0048583) | 50 | - | 6.22E-05 | 4.57E-03 |
| secretion (GO:0046903) | 16 | + | 3.51E-04 | 1.54E-02 |
| secretion by cell (GO:0032940) | 14 | + | 3.91E-05 | 3.23E-03 |
| small molecule metabolic process (GO:0044281) | 68 | + | 5.24E-04 | 2.04E-02 |
| translation (GO:0006412) | 13 | + | 8.18E-04 | 2.84E-02 |
| **GO Molecular Function** | | | | |
| actin binding (GO:0003779) | 38 | + | 1.76E-04 | 9.30E-03 |
| actin filament binding (GO:0051015) | 31 | + | 1.04E-04 | 1.46E-02 |
| anion binding (GO:0043168) | 48 | + | 2.06E-03 | 3.22E-02 |
| antigen binding (GO:0003823) | 9 | - | 1.03E-03 | 2.90E-02 |
| cytoskeletal protein binding (GO:0008092) | 44 | + | 1.09E-03 | 2.89E-02 |
| endopeptidase activity (GO:0004175) | 23 | - | 1.00E-03 | 3.03E-02 |
| glycosyltransferase activity (GO:0016757) | 22 | - | 1.02E-04 | 2.15E-02 |
| GTPase activity (GO:0003924) | 46 | + | 9.66E-04 | 3.14E-02 |
| hexosyltransferase activity (GO:0016758) | 17 | - | 2.08E-06 | 8.78E-04 |
| hydrolase activity, acting on acid anhydrides (GO:0016817) | 52 | + | 1.22E-04 | 8.57E-03 |
| hydrolase activity, acting on acid anhydrides, in phosphorus-containing anhydrides (GO:0016818) | 52 | + | 1.22E-04 | 1.03E-02 |
| hydrolase activity, acting on glycosyl bonds (GO:0016798) | 10 | - | 1.86E-04 | 7.87E-03 |
| hydrolase activity, hydrolyzing O-glycosyl compounds (GO:0004553) | 10 | - | 1.86E-04 | 8.75E-03 |
| ion binding (GO:0043167) | 71 | + | 1.40E-03 | 2.97E-02 |
| molecular transducer activity (GO:0060089) | 20 | - | 2.02E-03 | 3.42E-02 |
| nucleic acid binding (GO:0003676) | 21 | + | 7.55E-04 | 2.66E-02 |
| oxidoreductase activity, acting on CH-OH group of donors (GO:0016614) | 19 | + | 1.61E-03 | 2.95E-02 |
| oxidoreductase activity, acting on the CH-OH group of donors, NAD or NADP as acceptor (GO:0016616) | 19 | + | 1.61E-03 | 3.09E-02 |
| protein-containing complex binding (GO:0044877) | 69 | + | 1.53E-03 | 3.09E-02 |
| pyrophosphatase activity (GO:0016462) | 52 | + | 1.22E-04 | 1.29E-02 |
| receptor ligand activity (GO:0048018) | 5 | - | 1.22E-03 | 2.72E-02 |
| ribonucleoside triphosphate phosphatase activity (GO:0017111) | 50 | + | 4.68E-04 | 1.80E-02 |
| RNA binding (GO:0003723) | 12 | + | 2.02E-03 | 3.55E-02 |
| signaling receptor activator activity (GO:0030546) | 5 | - | 1.22E-03 | 3.04E-02 |
| signaling receptor activity (GO:0038023) | 20 | - | 2.02E-03 | 3.29E-02 |
| signaling receptor regulator activity (GO:0030545) | 5 | - | 1.22E-03 | 2.87E-02 |
| transmembrane signaling receptor activity (GO:0004888) | 17 | - | 2.29E-03 | 3.46E-02 |
| UDP-glycosyltransferase activity (GO:0008194) | 11 | - | 1.36E-04 | 8.21E-03 |
| **GO Cellular Component** | | | | |
| collagen-containing extracellular matrix (GO:0062023) | 17 | - | 4.55E-04 | 1.14E-02 |
| cytoplasm (GO:0005737) | 420 | + | 2.90E-06 | 1.45E-04 |
| cytosol (GO:0005829) | 99 | + | 3.89E-08 | 3.24E-06 |
| endoplasmic reticulum (GO:0005783) | 53 | - | 1.03E-03 | 2.14E-02 |
| external encapsulating structure (GO:0030312) | 20 | - | 6.12E-05 | 2.19E-03 |
| extracellular matrix (GO:0031012) | 20 | - | 6.12E-05 | 2.55E-03 |
| extracellular region (GO:0005576) | 146 | - | 0.00E00 | 0.00E00 |
| extracellular space (GO:0005615) | 140 | - | 0.00E00 | 0.00E00 |
| Golgi membrane (GO:0000139) | 9 | - | 2.52E-03 | 4.20E-02 |
| intracellular anatomical structure (GO:0005622) | 463 | + | 1.40E-06 | 8.75E-05 |
| intracellular non-membrane-bounded organelle (GO:0043232) | 62 | + | 2.27E-04 | 7.10E-03 |
| non-membrane-bounded organelle (GO:0043228) | 62 | + | 2.27E-04 | 6.31E-03 |
| nucleus (GO:0005634) | 75 | + | 6.64E-04 | 1.51E-02 |
| postsynapse (GO:0098794) | 9 | + | 2.25E-03 | 4.01E-02 |
| ribonucleoprotein complex (GO:1990904) | 9 | + | 1.07E-03 | 2.06E-02 |
| **Reactome** | | | | |
| Cell Cycle (R-BTA-1640170) | 47 | + | 3.55E-04 | 2.30E-02 |
| Chondroitin sulfate/dermatan sulfate metabolism (R-BTA-1793185) | 8 | - | 7.99E-05 | 8.79E-03 |
| Complement cascade (R-BTA-166658) | 22 | - | 9.65E-10 | 1.06E-06 |
| Extracellular matrix organization (R-BTA-1474244) | 38 | - | 2.81E-04 | 2.06E-02 |
| Glycosaminoglycan metabolism (R-BTA-1630316) | 17 | - | 1.05E-05 | 2.32E-03 |
| Heparan sulfate/heparin (HS-GAG) metabolism (R-BTA-1638091) | 9 | - | 1.08E-04 | 9.95E-03 |
| Initial triggering of complement (R-BTA-166663) | 8 | - | 2.50E-04 | 1.97E-02 |
| Metabolism of RNA (R-BTA-8953854) | 31 | + | 1.68E-04 | 1.43E-02 |
| Platelet degranulation (R-BTA-114608) | 32 | - | 7.83E-05 | 9.58E-03 |
| Post-translational protein phosphorylation (R-BTA-8957275) | 32 | - | 7.66E-07 | 2.11E-04 |
| Regulation of Complement cascade (R-BTA-977606) | 22 | - | 9.65E-10 | 5.31E-07 |
| Regulation of Insulin-like Growth Factor (IGF) transport and uptake by Insulin-like Growth Factor Binding Proteins (IGFBPs) (R-BTA-381426) | 32 | - | 7.66E-07 | 2.81E-04 |
| Response to elevated platelet cytosolic Ca2+ (R-BTA-76005) | 33 | - | 2.91E-04 | 2.00E-02 |
| Signaling by Rho GTPases (R-BTA-194315) | 75 | + | 3.32E-05 | 4.57E-03 |
| Signaling by Rho GTPases, Miro GTPases and RHOBTB3 (R-BTA-9716542) | 75 | + | 3.32E-05 | 5.22E-03 |
| Terminal pathway of complement (R-BTA-166665) | 8 | - | 2.14E-05 | 3.92E-03 |
| Translation (R-BTA-72766) | 16 | + | 8.10E-05 | 8.10E-03 |
| **Protein Class** | | | | |
| actin or actin-binding cytoskeletal protein (PC00041) | 41 | + | 4.75E-03 | 4.15E-02 |
| complement component (PC00078) | 9 | - | 3.26E-05 | 1.14E-03 |
| defense/immunity protein (PC00090) | 60 | - | 1.27E-07 | 1.77E-05 |
| G-protein (PC00020) | 48 | + | 4.70E-04 | 7.32E-03 |
| glycosidase (PC00110) | 6 | - | 6.01E-03 | 4.95E-02 |
| glycosyltransferase (PC00111) | 15 | - | 4.12E-05 | 1.15E-03 |
| growth factor (PC00112) | 7 | - | 1.21E-03 | 1.69E-02 |
| heterotrimeric G-protein (PC00117) | 10 | + | 3.01E-03 | 3.24E-02 |
| immunoglobulin (PC00123) | 12 | - | 3.23E-05 | 1.51E-03 |
| intercellular signal molecule (PC00207) | 11 | - | 4.47E-05 | 1.04E-03 |
| non-motor actin binding protein (PC00165) | 17 | + | 2.87E-03 | 3.35E-02 |
| non-receptor serine/threonine protein kinase (PC00167) | 12 | + | 3.29E-03 | 3.29E-02 |
| oxidoreductase (PC00176) | 59 | + | 1.40E-03 | 1.78E-02 |
| protease inhibitor (PC00191) | 29 | - | 1.22E-05 | 8.51E-04 |
| translation factor (PC00223) | 7 | + | 4.41E-03 | 4.12E-02 |
| translational protein (PC00263) | 21 | + | 1.53E-04 | 3.06E-03 |
| transmembrane signal receptor (PC00197) | 23 | - | 2.67E-04 | 4.67E-03 |
